# Meta-Analysis of Genome-Wide Association Studies Identifies Six New Loci for Serum Calcium Concentrations

**DOI:** 10.1371/journal.pgen.1003796

**Published:** 2013-09-19

**Authors:** Conall M. O'Seaghdha, Hongsheng Wu, Qiong Yang, Karen Kapur, Idris Guessous, Annie Mercier Zuber, Anna Köttgen, Candice Stoudmann, Alexander Teumer, Zoltán Kutalik, Massimo Mangino, Abbas Dehghan, Weihua Zhang, Gudny Eiriksdottir, Guo Li, Toshiko Tanaka, Laura Portas, Lorna M. Lopez, Caroline Hayward, Kurt Lohman, Koichi Matsuda, Sandosh Padmanabhan, Dmitri Firsov, Rossella Sorice, Sheila Ulivi, A. Catharina Brockhaus, Marcus E. Kleber, Anubha Mahajan, Florian D. Ernst, Vilmundur Gudnason, Lenore J. Launer, Aurelien Mace, Eric Boerwinckle, Dan E. Arking, Chizu Tanikawa, Yusuke Nakamura, Morris J. Brown, Jean-Michel Gaspoz, Jean-Marc Theler, David S. Siscovick, Bruce M. Psaty, Sven Bergmann, Peter Vollenweider, Veronique Vitart, Alan F. Wright, Tatijana Zemunik, Mladen Boban, Ivana Kolcic, Pau Navarro, Edward M. Brown, Karol Estrada, Jingzhong Ding, Tamara B. Harris, Stefania Bandinelli, Dena Hernandez, Andrew B. Singleton, Giorgia Girotto, Daniela Ruggiero, Adamo Pio d'Adamo, Antonietta Robino, Thomas Meitinger, Christa Meisinger, Gail Davies, John M. Starr, John C. Chambers, Bernhard O. Boehm, Bernhard R. Winkelmann, Jie Huang, Federico Murgia, Sarah H. Wild, Harry Campbell, Andrew P. Morris, Oscar H. Franco, Albert Hofman, Andre G. Uitterlinden, Fernando Rivadeneira, Uwe Völker, Anke Hannemann, Reiner Biffar, Wolfgang Hoffmann, So–Youn Shin, Pierre Lescuyer, Hughes Henry, Claudia Schurmann, The SUNLIGHT consortium, The GEFOS consortium, Patricia B. Munroe, Paolo Gasparini, Nicola Pirastu, Marina Ciullo, Christian Gieger, Winfried März, Lars Lind, Tim D. Spector, Albert V. Smith, Igor Rudan, James F. Wilson, Ozren Polasek, Ian J. Deary, Mario Pirastu, Luigi Ferrucci, Yongmei Liu, Bryan Kestenbaum, Jaspal S. Kooner, Jacqueline C. M. Witteman, Matthias Nauck, W. H. Linda Kao, Henri Wallaschofski, Olivier Bonny, Caroline S. Fox, Murielle Bochud

**Affiliations:** 1 National Heart, Lung, and Blood Institute's Framingham Heart Study and Center for Population Studies, Framingham, Massachusetts, United States of America; 2 Renal Division, Massachusetts General Hospital, Boston, Massachusetts, United States of America; 3 Department of Biostatistics, Boston University, Boston, Massachusetts, United States of America; 4 Department of Medical Biology, University of Split, School of Medicine, Split, Croatia; 5 Department of Medical Genetics, University of Lausanne, Lausanne, Switzerland; 6 Institute of Social and Preventive Medicine (IUMSP), Lausanne University Hospital, Lausanne, Switzerland; 7 Unit of Population Epidemiology, Division of Primary Care Medicine, Department of Community Medicine and Primary Care and Emergency Medicine, Geneva University Hospitals, Geneva, Switzerland; 8 Geriatric Unit, Azienda Sanitaria Firenze (ASF), Florence, Italy; 9 Department of Pharmacology and Toxicology, University of Lausanne, Lausanne, Switzerland; 10 Renal Division, Freiburg University Hospital, Freiburg, Germany; 11 Department of Epidemiology, Johns Hopkins Bloomberg School of Public Health, Baltimore, Maryland, United States of America; 12 Interfaculty Institute for Genetics and Functional Genomics, Ernst-Moritz-Arndt-University Greifswald, Greifswald, Germany; 13 Swiss Institute of Bioinformatics, Lausanne, Switzerland; 14 King's College London, St. Thomas' Hospital Campus, London, United Kingdom; 15 Department of Epidemiology, Erasmus Medical Center, Rotterdam, The Netherlands; 16 Catheter Lab, Cardiology, Ealing Hospital, Southall, Middlesex, United Kingdom; 17 Department of Epidemiology and Biostatistics, School of Public Health, Imperial College London, London, United Kingdom; 18 Icelandic Heart Association Research Institute, Kopavogur, Iceland; 19 Cardiovascular Health Research Unit, University of Washington, Seattle, Washington, United States of America; 20 Clinical Research Branch, National Institute on Aging, Baltimore, Maryland, United States of America; 21 Institute of Population Genetics, CNR-Traversa La Crucca, Reg. Baldinca Li Punti, Sassari, Italy; 22 Centre for Cognitive Ageing and Cognitive Epidemiology, The University of Edinburgh, Edinburgh, United Kingdom; 23 MRC Human Genetics Unit, MRC IGMM, University of Edinburgh, Edinburgh, United Kingdom; 24 Cardiology Group, ClinPhenomics GmbH&Co KG, Frankfurt-Sachsenhausen, Germany; 25 Laboratory of Molecular Medicine, Human Genome Center, Institute of Medical Science, University of Tokyo, Tokyo, Japan; 26 BHF Glasgow Cardiovascular Research Centre, Division of Cardiovascular and Medical Sciences, University of Glasgow, Glasgow, Scotland; 27 Institute of Genetics and Biophysics ‘Adriano-Buzzati Traverso’, CNR, Napoli, Italy; 28 Institute for Maternal and Child Health - IRCCS “Burlo Garofolo”, Trieste, Italy; 29 Institute of Genetic Epidemiology, Helmholtz Zentrum München - German Research Center for Environmental Health, Neuherberg, Germany; 30 Department of Medicine I, University Hospital Grosshadern, Ludwig-Maximilians University Munich, Munich, Germany; 31 Department of Internal Medicine II – Cardiology, University of Ulm Medical Centre, Ulm, Germany; 32 Mannheim Institute of Public Health, Social and Preventive Medicine, Medical Faculty Mannheim, University of Heidelberg, Mannheim, Germany; 33 Wellcome Trust Centre for Human Genetics, Roosevelt Drive, Oxford, United Kingdom; 34 University of Iceland, Reykjavik, Iceland; 35 Laboratory of Epidemiology, Demography and Biometry, National Institute on Aging, Bethesda, Maryland, United States of America; 36 University of Texas Health Science Center at Houston, Houston, Texas, United States of America; 37 McKusick-Nathans Institute of Genetic Medicine, Johns Hopkins University School of Medicine, Baltimore, Maryland, United States of America; 38 Cambridge Institute of Medical Research, University of Cambridge, Cambridge, United Kingdom; 39 Department of Internal Medicine, Erasmus Medical Center, Rotterdam, The Netherlands; 40 Departments of Medicine and Epidemiology, University of Washington, Seattle, Washington, United States of America; 41 Group Health Research Institute, Group Health Cooperative, Seattle, Washington, United States of America; 42 Departments of Medicine, Epidemiology and Health Services, University of Washington, Seattle, Washington, United States of America; 43 Department of Medicine, Internal Medicine, Lausanne University Hospital, Lausanne, Switzerland; 44 Faculty of Medicine, University of Split, Split, Croatia; 45 Department of Pharmacology, Faculty of Medicine, University of Split, Split, Croatia; 46 Division of Laboratory Medicine, Geneva University Hospitals, Geneva, Switzerland; 47 Department of Internal Medicine, Wake Forest School of Medicine, Winston-Salem, North Carolina, United States of America; 48 Department of Human Genetics, Wellcome Trust Sanger Institute, Hinxton, Cambridge, United Kingdom; 49 Molecular Genetics Section, Laboratory of Neurogenetics, National Institute on Aging, National Institutes of Health, Bethesda, Maryland, United States of America; 50 Institute of Human Genetics, Helmholtz Zentrum München - German Research Center for Environmental Health, Neuherberg, Germany; 51 Department of Computer Science and Networking, Wentworth Institute of Technology, Boston, Massachusetts, United States of America; 52 Institute of Epidemiology II, Helmholtz Zentrum München - German Research Center for Environmental Health, Neuherberg, Germany; 53 Epidemiology and Biostatistics, Imperial College London, Norfolk Place, London, United Kingdom; 54 Ulm University Medical Centre, Department of Internal Medicine I, Ulm University, Ulm, Germany; 55 LKC School of Medicine, Imperial College London and Nanyang Technological University, Singapore, Singapore; 56 Department of Epidemiology, Rollins School of Public Health, Emory University, Atlanta, Georgia, United States of America; 57 Division of Primary Care Medicine, Department of Community Medicine and Primary Care and Emergency Medicine , Geneva University Hospitals, Geneva, Switzerland; 58 Centre for Population Health Sciences, The University of Edinburgh Medical School, Edinburgh, Scotland, United Kingdom; 59 Institute of Clinical Chemistry and Laboratory Medicine, University Medicine Greifswald, Ernst-Moritz-Arndt University Greifswald, Greifswald, Germany; 60 Department of Prosthetic Dentistry, Gerostomatology and Dental Materials, University Medicine Greifswald, Greifswald, Germany; 61 Institute for Community Medicine, University Medicine Greifswald, Greifswald, Germany; 62 Human Genetics, Wellcome Trust Sanger Institute, Hinxton, United Kingdom; 63 Department of Biostatistical Sciences, Division of Public Health Sciences, Wake Forest School of Medicine, Winston-Salem, North Carolina, United States of America; 64 Clinical Chemistry Laboratory, Lausanne University Hospital, Lausanne, Switzerland; 65 William Harvey Research Institute, Barts and The London School of Medicine and Dentistry, Queen Mary University of London, London, United Kingdom; 66 Synlab Centre of Laboratory Diagnostics, Heidelberg, Germany; 67 Institute of Medical Sciences, Uppsala University Hospital, Uppsala, Sweden; 68 Department of Epidemiology and Prevention, Division of Public Health Sciences, Wake Forest School of Medicine, Winston-Salem, North Carolina, United States of America; 69 Department of Medicine, Division of Nephrology, University of Washington, Seattle, Washington, United States of America; 70 Faculty of Medicine, National Heart & Lung Institute, Cardiovascular Science, Hammersmith Hospital, Hammersmith Campus, Imperial College London, London, United Kingdom; 71 Imperial College Healthcare NHS Trust, London, United Kingdom; 72 Welch Center for Prevention, Epidemiology and Clinical Research, John Hopkins University, Baltimore, Maryland, United States of America; 73 Service of Nephrology, Lausanne University Hospital, Lausanne, Switzerland; 74 Division of Endocrinology, Brigham and Women's Hospital and Harvard Medical School, Boston, Massachusetts, United States of America; University of Michigan, United States of America

## Abstract

Calcium is vital to the normal functioning of multiple organ systems and its serum concentration is tightly regulated. Apart from *CASR*, the genes associated with serum calcium are largely unknown. We conducted a genome-wide association meta-analysis of 39,400 individuals from 17 population-based cohorts and investigated the 14 most strongly associated loci in ≤21,679 additional individuals. Seven loci (six new regions) in association with serum calcium were identified and replicated. Rs1570669 near *CYP24A1* (*P* = 9.1E-12), rs10491003 upstream of *GATA3* (*P* = 4.8E-09) and rs7481584 in *CARS* (*P* = 1.2E-10) implicate regions involved in Mendelian calcemic disorders: Rs1550532 in *DGKD* (*P* = 8.2E-11), also associated with bone density, and rs7336933 near *DGKH/KIAA0564* (*P* = 9.1E-10) are near genes that encode distinct isoforms of diacylglycerol kinase. Rs780094 is in *GCKR*. We characterized the expression of these genes in gut, kidney, and bone, and demonstrate modulation of gene expression in bone in response to dietary calcium in mice. Our results shed new light on the genetics of calcium homeostasis.

## Author Summary

Calcium is vital to many biological processes and its serum concentration is tightly regulated. Family studies have shown that serum calcium is under strong genetic control. Apart from *CASR*, the genes associated with serum calcium are largely unknown. We conducted a genome-wide association meta-analysis of 39,400 individuals from 17 population-based cohorts and investigated the 14 most strongly associated loci in ≤21,679 additional individuals. We identified seven loci (six new regions) as being robustly associated with serum calcium. Three loci implicate regions involved in rare monogenic diseases including disturbances of serum calcium levels. Several of the newly identified loci harbor genes linked to the hormonal control of serum calcium. In mice experiments, we characterized the expression of these genes in gut, kidney, and bone, and explored the influence of dietary calcium intake on the expression of these genes in these organs. Our results shed new light on the genetics of calcium homeostasis and suggest a role for dietary calcium intake in bone-specific gene expression.

## Introduction

Normal calcium homeostasis is regulated by three major hormones acting on their corresponding receptors in gut, kidney, and bone: parathyroid hormone (PTH) release governed by the calcium-sensing receptor (CASR), calcitonin, and the active metabolite of vitamin D, 1,25(OH)_2_-D. Despite heritability estimates of 33–78%, the genetic determinants of serum calcium are poorly understood [1], [2], [3]. We have previously reported a variant in *CASR* associated with calcium concentrations in European-ancestry individuals [4], [5]. To detect additional loci, we conducted a two-stage genome-wide association meta-analysis of serum calcium and studied expression of identified genes in key calcium homeostatic organs in the mouse under various calcium diets.

## Results

### Genome-wide association meta-analysis in Europeans

The discovery analysis consisted of 39,400 individuals from 17 population-based cohorts of European descent (**Table 1** and **Table S1**). There was little evidence for population stratification at study level (median genomic inflation factor, λ = 1.006) or meta-analysis level (λ = 1.03), and we detected an excess of association signals beyond those expected by chance (**Figure S1**).

**Table 1:** Genome-wide significant and replicated loci for serum calcium in Europeans.

						Discovery analysis					Replication analysis					Meta-analysis				
Markers*	chr	Position	Nearby Genes	A1	A2	N	Freq A1	Effect A1	SE	*P value*	N	Freq A1	Effect A1	SE	*P value* *	N	Freq A1	Effect A1	SE	*P value*
**Known locus**																				
rs1801725	3	123486447	*CASR*	t	g	39400	0.15	0.069	0.004	6.5E-59	21654	0.15	0.076	0.007	3.6E-30	61054	0.15	0.071	0.004	8.9E-86
**Novel loci**																				
rs1550532	2	233929587	*DGKD*	c	g	39400	0.31	0.018	0.003	4.6E-08	21598	0.31	0.019	0.005	0.0002	60998	0.31	0.018	0.003	8.2E-11
rs780094	2	27594741	*GCKR*	t	c	39400	0.41	0.020	0.003	3.7E-11	21558	0.42	0.008	0.005	0.049	60958	0.42	0.017	0.003	1.3E-10
rs10491003	10	9368657	*GATA3*	t	c	38361	0.09	0.027	0.006	1.6E-06	21679	0.10	0.028	0.008	0.0003	60040	0.09	0.027	0.005	4.8E-09
rs7481584	11	2985665	*CARS*	a	g	39400	0.29	−0.021	0.003	9.2E-10	21611	0.30	−0.013	0.005	0.008	61011	0.30	−0.018	0.003	1.2E-10
rs7336933	13	41457076	*DGKH; KIAA0564*	a	g	39400	0.15	−0.023	0.004	1.6E-07	21528	0.14	−0.022	0.007	0.0009	60928	0.15	−0.022	0.004	9.1E-10
rs1570669	20	52207834	*CYP24A1*	a	g	39400	0.66	−0.018	0.003	4.0 E-08	21566	0.66	−0.020	0.005	4.5E-05	60966	0.66	−0.018	0.003	9.1E-12

P values are corrected for inflation using genomic control. Replication criteria: overall genome-wide significance (*P*<5E-8) and one-sided replication *P*<0.05. I^2^ was zero for rs1801725, rs1550532, rs10491003, rs7336933 and rs1570669 (I^2^ *P*>0.20). For rs780094 and rs7481584, I^2^ were 0.79 and 0.43 with I^2^ *P* 0.03 and 0.19, respectively. For these latter SNPs, sample size weighted meta-analysis P values were 2.93E-10 and 2.03E-10, respectively.

Chr, chromosome. Effect A1 = beta regression coefficient for allele A1; SE, standard error.

* one-sided P values.

**Figure 1:**
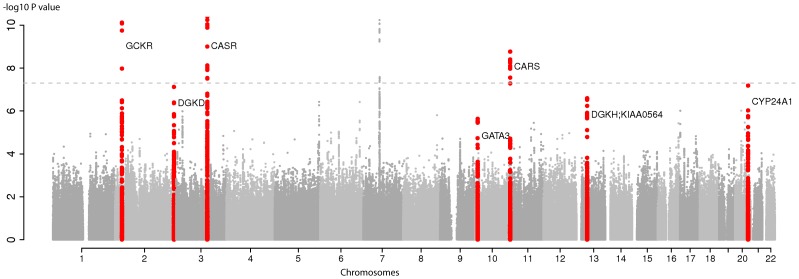
Genome-wide association for serum calcium in discovery analysis in Europeans. Manhattan plot showing −log_10_(P values) for all SNPs in the discovery GWAS for uncorrected serum calcium in Europeans (N = 39,400), ordered by chromosomal position. The plot is truncated at −log10 P values of 10 (truncated −log_10_P values for GCKR and CASR). The values correspond to the association of uncorrected serum calcium, including age and sex as covariates in the model as well as study-specific covariates if needed. The gene closest to the SNP with the lowest P value is listed at each locus. Six loci reached genome-wide significance (*P*<5E-08) at discovery analysis (*GCKR*, *DGKD*, *CASR*, *VKORC1L1* (in grey on chromosome 7), *CARS* and *CYP24A1*. The seven loci that reached genome-wide significance at the combined analysis following replication are highlighted in red (*GCKR*, *DGKD*, CASR, *GATA3*, *CARS*, *DGKH*-KIAA0564 and *CYP24A1*).

The *CASR* locus, previously identified in Europeans, was confirmed in our meta-analysis (*P* = 6.5E-59, **Figure S2**) [4], [5]. In addition, SNPs from five independent regions reached genome-wide significance (*P*<5E-08) in the overall discovery meta-analysis (**Figure 1** **,** **Table 1** **, Table S2**): rs1550532 (in *DGKD*, *P* = 4.60E-08), rs780094 (in *GCKR*; *P* = 3.69E-11), rs17711722 (near *VKORC1L1*, *P* = 2.78E-11), rs7481584 (in *CARS*, *P* = 9.21E-10) and rs1570669 (near *CYP24A1*; *P* = 3.98E-08).

Fourteen SNPs from Stage 1 were sent for Stage 2 validation in ≤21,679 additional Europeans: the twelve independent (≥1 Mb apart) SNPs with lowest P values (6.5E-59 to 8.1E-06) in Europeans and two additional genome-wide significant loci (rs9447004 and rs10491003) from a combined sample including 8318 Indian-Asians (**Table 1**). Of the fourteen SNPs, seven were considered successfully replicated (i.e. were in the same direction of effect as the discovery meta-analysis, had a one-side replication *P*<0.05 and were genome-wide significant (*P*<5E-8) in combined meta-analysis of discovery and replication sets). These were rs1801725 in *CASR*, rs1550532 in *DGKD*, rs780094 in *GCKR*, rs7336933 near *KIAA0564* and *DGKH*, rs10491003 (closest gene *GATA3*), rs7481584 in *CARS* and rs1570669 near *CYP24A1* (**Table 1**). Regional association plots are presented in **Figure S3**. Details on the seven SNPs that did not replicate are presented in **Table S2**. Association results for serum calcium in Caucasians for all SNPs with P value<5*E-5 are listed in **Table S3**. In a secondary analysis, all SNPs identified in the primary analysis showed consistent and significant association with serum calcium adjusted for serum albumin (**Table S4, Figure S4**), as well as an excess of association signals beyond those expected by chance (**Figure S5**); no additional locus was identified using albumin-corrected serum calcium (**Table S5**).

### Copy number variations (CNVs) and eQTL analyses

We found no significant association of the 7 replicated SNPs known to provide reliable tags for copy number variations (CNVs) in people of European-descent from the Hypergene dataset. For all the SNPs, the calculated correlation was below 0.002. We also explored a list of SNPs tagging CNVs from the GIANT consortium. Out the 7 SNPs tested, only the rs1570669 was in slight linkage disequilibrium (r^2^ = 0.54) with one SNP of the WTCCC2 list (rs927651). The corresponding SNP tags the CNVR7875.1 CNV located 455b from the SNP of interest.

For each of the 7 replicated SNPs, we identified all proxy SNPs with r^2^>0.8 in HapMap CEU (releases 21, 22, and HapMap 3 version 2) using the online SNAP database (http://www.broadinstitute.org/mpg/snap/). This led to the identification of 40 SNPs. We then queried each of these SNPs in the eQTL database of the University of Chicago (http://eqtl.uchicago.edu/cgi-bin/gbrowse/eqtl/). Three of the seven SNPs are in strong linkage disequilibrium with an eQTL, as illustrated in **Table S6**.

### Information on genes mapping into the replicated genomic regions

Proposed functions of the genes mapping into the associated intervals (±250 kb) are in **Box 1** **and** in **Table S7** for the gene-rich *GCKR* region. We report in **Table S8** the mechanism and/or location of all available biological processes, cellular components and molecular functions related to the genes mapping into the associated intervals from the AmiGo 1.8 gene ontology database. We also queried the OMIM database for each genes located within ±250 kb of the replicated loci (**Table S9**)

**Box 1. Genes Located within Replicated Loci for Serum Calcium**

We here summarize the information on genes located within ±250 kb from the top SNP at each locus. Because it is a gene dense region, details of genes located in the *GCKR* genomic region are presented in **Table S4**.

**Chromosome 2, locus rs1550532**

***DGKD*** rs1550532 is an intronic SNP located near the 5′UTR region of *DGKD*. DGKD encodes diacylglycerol kinase delta, a member of the diacylglycerol kinase (DGK) enzyme family. Alternative splicing of the DGKD gene results in two isoforms, which differ in their expression profiles and regulatory mechanisms [24]. DGKs play an important role in signal transduction by modulating the balance between the diacylglycerol (DAG) and phosphatidic acid (PA), important second messengers in signaling cascades. Recent findings suggest that DAG is involved in calcium signaling in parathyroid cells [25]. CASR signaling influences intracellular DAG levels in cardiomyocytes [26].

***SAG*** encodes S-antigen (also called arrestin), a soluble photoreceptor protein expressed in the retina and pineal gland. Mutations in this gene are associated with Oguchi disease (OMIM#258100), a rare autosomal recessive form of night blindness. Arrestin is a calcium-binding protein that plays an important role in phototransduction.

***ATG16L1*** encodes autophagy related 16-like 1 protein, part of a complex involved in autophagia. Mutations in this gene are responsible for inflammatory bowel disease 10 (OMIM # 611081). There is no known direct link with calcium signaling.

***SCARNA5 and SCARNA6*** encode small Cajal body-specific RNAs 5 and 6, which are small nuclear RNAs, belonging to non-coding RNAs involved in the RNA-processing machinery. There is no known direct link with calcium signaling.

***USP40*** encodes ubiquitin specific peptidase 40. USP40 functions as a deubiquinating enzyme involved in the degradation of unwanted intracellular proteins in eukaryocytic cells. There is no known direct link with calcium signaling.

***INPP5D*** encode inositol polyphosphate-5-phosphatase, expressed in hematopoietic cells. This protein regulates myeloid cell proliferation. The presence of a recombination peak between this gene and rs1550532 makes it an unlikely candidate for this signal.

**Chromosome 10, locus rs10491003**

rs10491003, located within a long non-coding RNA with *GATA3* as its nearest gene may influence the expression of *GATA3* [27].

***GATA3***: *GATA3* encodes a GATA transcription factor involved in T cell lymphopoiesis [28], renal and vestibular morphogenesis [29], [30], and parathyroid gland development [31]. *GATA3* haploinsufficiency causes hypoparathyroidism and hypocalcemia in the autosomal dominant HDR syndrome (hypoparathyroidism, sensorineural deafness and renal dysplasia) (OMIM#146255) [32], [33]. Although *GATA3* is the closest gene to rs10491003, this variant lies 1.2 Mbp downstream from that gene. However, *GATA3* has a very large flanking regulatory region - greater than 450 kbp - [34] and mammalian enhancers may lie more than 1 Mbp away from the gene they regulate [35]. *GATA3* may play a role in preserving high degree of differentiation of parathyroid gland and of calcium transporting epithelia [36].

**Chromosome 11, locus rs7481584**

This region is located in the imprinted gene domain of 11p15.5, an important tumor suppressor gene region [37].

***CARS*** *:* rs7481584 is an intronic SNP of *CARS*. *CARS* encodes a cysteinyl-tRNA synthetase and is located within the imprinted gene domain of 11p15.5. This region is linked to Beckwith-Wiedemann syndrome, which is associated with hypocalcemia and hypercalciuria.

***NAP1L4*** encodes nucleosome assembly protein 1-like 4, a member of the nucleosome assembly protein, potentially involved in histone chaperoning and ubiquitously expressed. NAP1L1 and NAP1L4 have been recently identified as being involved in the regulation of DGKH nucleocytoplasmic shuttling [38]. A link with calcium homeostasis could be possible via the DGKs pathway.

***PHLDA2*** encodes pleckstrin homology-like domain, family A, member 2. This gene has been recently highlighted as potentially relevant for osteoporosis on the basis of a bioinformatics pathway analysis approach [39]. Imprinting of this gene appears to play a role in fetal growth, including fetal bone growth, birth weight and bone mass in childhood.[40], [41], [42], [43] In cancer, PHLDA2 is activated by parathyroid hormone-like hormone (PTHLH) [44]. PTHLH is associated with malignancy-related hypercalcemia [45], lactation [46], the expression of PHLDA2 is upregulated in osteosarcoma progression [47].

***OSBPL5*** encodes oxysterol binding protein-like 5, an intracellular lipid receptor involved in cholesterol balance. There is no known direct link with calcium homeostasis.

***MRGPRE and MRGPRG*** encode MAS-related G-protein-coupled receptors, member E and G. This family of receptors is expressed in nociceptive sensory neurons. There is no known direct link with calcium homeostasis.

***C11orf36*** encodes MRGPRG antisense RNA 1. Little is known about this gene.

***SNORA54*** encodes small nucleolar RNA, H/ACA box. The gene product belongs to non-coding RNAs involved in the RNA-processing machinery. There is no known direct link with calcium homeostasis.

***SLC22A18 and SLC22A18AS*** encode solute carrier family 22, member 1 and solute carrier family 22, member 1 antisense. SLC22A18 is an organic cation transporter. Mutations in *SLC22A18* have been found in several cancers. There is no known direct link with calcium homeostasis.

***CDKN1C*** encodes cyclin-dependent kinase inhibitor 1C (p57, Kip2), a protein involved in cell-cycle progression. This imprinted gene is responsible for the IMAGe syndrome (OMIM#300290) characterized by intrauterine growth restriction, metaphyseal dysplasia, delayed bone aging, adrenal hypoplasia congenital, genital anomalies, and sometimes hypercalciuria [48].

***KCNQ1*** encode potassium voltage-gated channel, KQT-like subfamily, member 1. ***KCNQ1OT1*** represents KCNQ1 opposite strand transcript 1 and is an unspliced long non-coding RNA, which regulates the transcription of many target genes. Mutations in *KCNQ1* are associated with hereditary long and short QT syndromes (OMIM#192500 & 609621), Jervell and Lange-Nielsen syndrome (OMIM#220400), familial atrial fibrillation (OMIM#607554), type 2 diabetes. *KCNQ1* is also imprinted in a tissue-specific manner. There is no known direct link with calcium homeostasis.

**Chromosome 13, locus rs7336933**

***DGKH*** encodes diacylglycerol kinase eta, a member of the diacylglycerol kinase (DGK) enzyme family. See *DGKD* (above) for discussion.

***KIAA0564***: this gene encodes a large uncharacterized protein containing a putative ATP-ase domain. The sequence of this gene is conserved across a large array of organisms, from humans to mouse, zebrafish and to *C. elegans*, which suggests an important biological function. Yet, little is known on the nature of the function of this gene so far.

**Chromosome 20, locus rs1570669**

***CYP24A1***: rs1570669 is an intronic SNP of *CYP24A1*. *CYP24A1* encodes a cytochrome P450 enzyme that hydroxylates 1,25-(OH)_2_D, into metabolites targeted for degradation and appears to be one of the central regulator of 1,25-(OH)_2_-D metabolism. CYP24A1 is highly regulated by its own substrate 1,25(OH)_2_-D, as well as by PTH [49], [50], serum phosphate and fibroblast growth factor-23 (FGF-23) [51], [52], [53]. Sequence variants of *CYP24A1* impacting on 1,25(OH)_2_-D metabolism have been described recently and explain the strong heritability of 1,25(OH)_2_-D concentrations.

***BCAS1*** encodes breast carcinoma amplified sequence 1, considered as an oncogene. *BCAS1* is highly differentially expressed in some cancers. However, there is no direct link with calcium homeostasis.

***PFDN4*** encodes prefoldin subunit4. Prefoldin is a chaperone complex involved in polypeptide folding. There is no known link of this gene with calcium homeostasis.

### Validation across ethnicities

In Indian-Asians, all 7 replicated SNPs had beta-coefficients that were direction-consistent with the primary analysis and 3 were statistically significant (*P*<0.05): rs1801725 (*CASR, P* = 1.4E-31), rs1550532 (*DGKD, P* = 0.002) and rs10491003 (*GATA3, P* = 0.009) (**Table S10**). In Japanese, 3 SNPs had betas that were direction-consistent with the primary analysis, but only rs1801725 (*CASR*) was associated with serum calcium (*P* = 0.001) (**Table S10**).

### Associations with related phenotypic traits

We conducted analyses of related bone mineral and endocrine phenotypic traits for the 7 replicated loci (**Table 2**). Several SNPs were associated (*P*<0.05) with bone mineral density (BMD) in the GEFOS consortium [6]: rs1801725 at *CASR* (*P* = 0.025; previously reported [4], [5]) and rs780094 (*GCKR*) at the lumbar spine (*P* = 0.006), rs1570669 at *CYP24A1* at the femoral neck (*P* = 0.04), and rs1550532 at *DGKD* at both the lumbar spine (*P* = 0.003) and the femoral neck (*P* = 0.003). For endocrine phenotypes, rs1570669 at *CYP24A1* was associated with higher PTH concentrations (*P* = 0.0005) and rs1801725 at *CASR* with higher serum PTH concentrations (*P* = 0.028) and lower serum phosphate concentrations, as previously reported [4], [5]. No SNP was associated significantly with circulating 25-OH vitamin D concentrations (all *P*>0.05) in the SUNLIGHT consortium [7].

**Table 2:** Look-ups of serum calcium loci with related phenotypes: bone mineral density in the GEFOS dataset [6] and endocrine phenotypes from the SHIP, SHIP Trend and SUNLIGHT [7] datasets.

			Lumbar bone density				Femoral bone density				Serum phosphorus				25OH Vitamin D			Parathyroid hormone			
Markers	Gene	A1	N	Effect A1	SE	*P value*	N	Effect A1	SE	*P value*	N	Effect A1	SE	*P value*	N	zscore	*P value*	N	Effect A1	SE	*P value*
**rs1801725**	***CASR***	T	**31791**	**−0.029**	**0.013**	**0.03**	32948	−0.011	0.012	0.4	16190	**−0.038**	**0.008**	**3.4E-07**	22537	−0.426	0.7	4181	**0.031**	**0.014**	**0.03**
**rs1550532**	***DGKD***	C	**31681**	**−0.028**	**0.009**	**0.003**	**32845**	**−0.025**	**0.009**	**0.003**	16190	**−0.013**	**0.006**	**0.03**	20371	−0.888	0.4	4181	**0.032**	**0.010**	**0.002**
**rs780094**	***GCKR***	T	**31783**	**−0.024**	**0.009**	**0.006**	32946	−0.009	0.008	0.3	16190	**0.011**	**0.005**	**0.03**	22520	−0.699	0.5	4181	0.0002	0.010	1.0
**rs10491003**	***GATA3***	T	31797	0.007	0.016	0.6	32740	0.015	0.015	0.3	16190	−0.001	0.010	0.9	22543	−1.328	0.2	4181	0.018	0.018	0.3
**rs7481584**	***CARS***	A	31667	0.013	0.009	0.2	32948	0.006	0.009	0.5	16190	0.011	0.006	0.08	20366	−1.630	0.1	4181	−0.006	0.011	0.6
**rs7336933**	***DGKH***	A	30992	−0.006	0.013	0.7	32152	0.000	0.012	1.0	16190	0.0115	0.008	0.1	20437	0.648	0.5	4181	−0.010	0.013	0.4
**rs1570669**	***CYP24A1***	A	31739	−0.004	0.009	0.7	32900	**−0.017**	**0.009**	**0.04**	16190	0.0040	0.006	0.5	20385	0.144	0.9	4181	**0.035**	**0.010**	**0.0005**

NA, not available. P values<0.05 were considered as statistically significant. A1, effect allele. β, regression coefficient for allele A1, SE, standard error. P, two-sided P value. Zscore, z score.

### Animal studies

**Figure 2:**
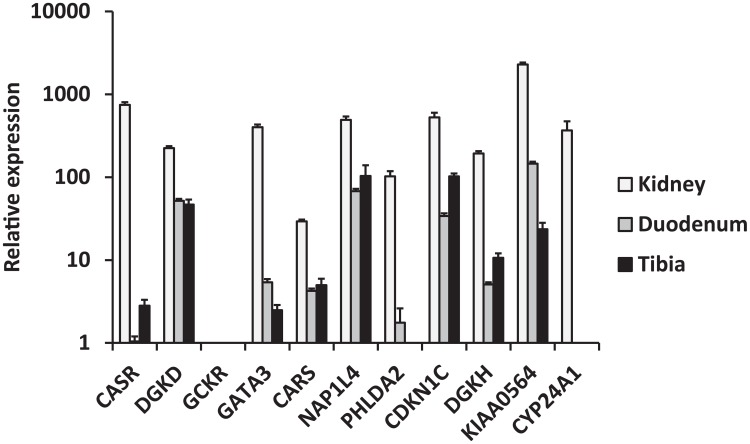
Relative mRNA expression of replicated genes in three calcium-transporting tissues (kidney, duodenum, tibia). The expression (based on delta CT [cycle threshold] normalized to actin) of the selected genes is compared to the expression of the *CASR* gene in the duodenum, thereby providing a relative expression. Cut-off was set at delta CT≤15. Data are means ± standard error of the mean (SEM) of values obtained from 5 mice fed a normal diet. *GCKR* was not expressed.

**Figure 3:**
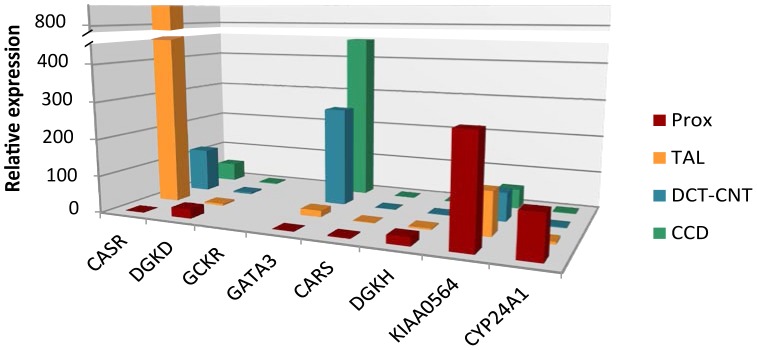
Relative mRNA expression of identified genes in kidney tubule segments. The renal tubular segments analyzed were the proximal tubule (PROX), the thick ascending limb of the loop of Henle (TAL), the distal convoluted tubule and connecting tubule (DCT-CNT), and the cortical collecting duct (CCD). The expression (based on the delta CT [cycle threshold]) of the selected genes is compared to the expression of the *CASR* gene in the PROX, thereby providing a relative expression. Data are means of values obtained from 3 mice fed a normal diet. *GCKR* was not expressed.

We selected biologically plausible gene(s) at each locus for *in vivo* studies in a mouse model as described in **Methods'** section. We first analyzed gene expression in the three primary calcium-handling organs: duodenum, kidney and bone (tibia). *CASR* for the rs1801725 locus, *DGKD* for the rs1550532 locus, *GATA3* for the rs10491003 locus, *CARS*, *NAP1L4* and *CDKN1C* for the rs7481584 locus, *DGKH* and *KIAA0564* for the rs7336933 locus, were expressed in all organs, whereas *CYP24A1* (rs1570669 locus) was solely, and *PHLDA2* (rs7481584 locus) mainly, expressed in the kidney (**Figure 2**). No significant expression of *GCKR* (rs780094 locus) was observed in any organ tested, which is of interest considering the strong attenuation of the association of rs780094 with serum calcium after adjustment for albumin (**Table S4**). In micro-dissection of nephron segments [8], [9], *DGKD*, *DGKH*, *CARS*, *KIAA0564* and *CYP24A1* were primarily transcribed in the proximal tubule, *CASR* in the thick ascending limb, and *GATA3* predominantly in the distal nephron and collecting duct (**Figure 3**).

**Figure 4:**
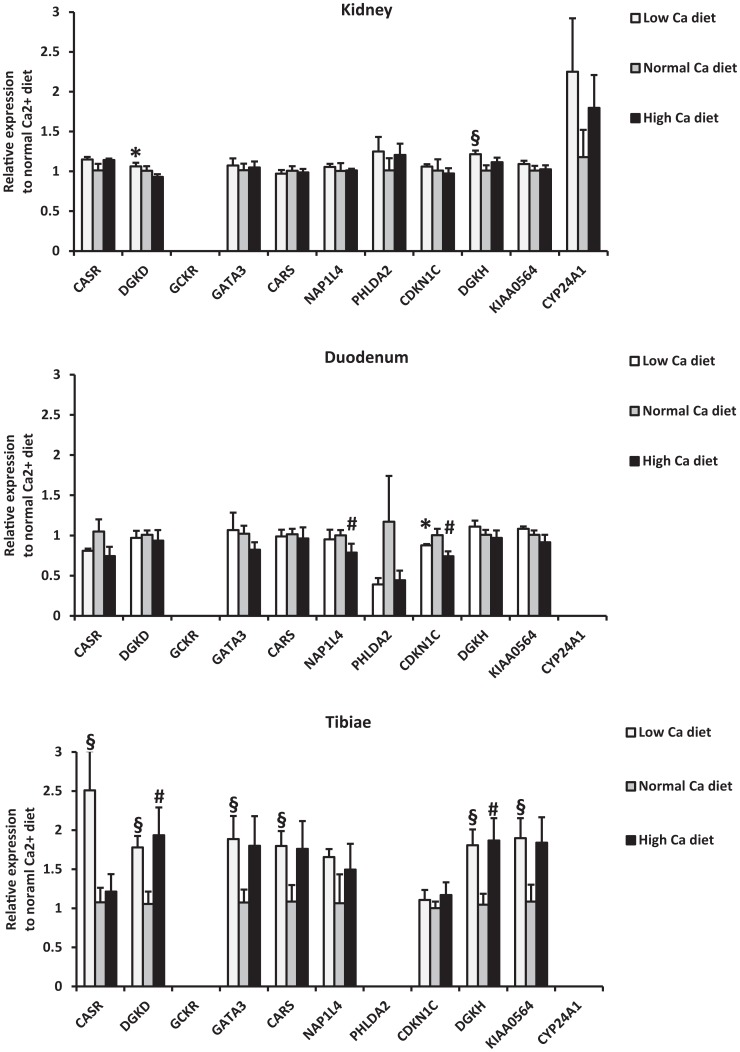
Relative mRNA expression of identified genes from mice fed a low (0.17%) and high (1.69%) calcium diet compared to mice fed a normal calcium diet (0.82%). Data are means± SEM of values obtained from 5 mice for each diet group. Expression levels were normalized to actin. Statistical significance of the difference between diets was calculated using unpaired t-test. *: *P*≤0.05 (low compared to high); §: *P*≤0.05 (low compared to normal); # *P*≤0.05 (high compared to normal).

In order to determine regulation of gene expression by calcium intake, we measured gene expression levels in mice fed low and high calcium diets (0.17% vs. 1.69% calcium) for one week, with normal diet as control (0.82%) (**Figure 4** **and Table S11**). In the kidney, both *DGKD* and *DGKH* were upregulated in response to low calcium diet (*P*≤0.05; **Figure 4**). In the tibia, *CASR* was markedly upregulated in response to low calcium diet (2.5-fold increased expression), as were *GATA3*, *KIAA0564* and *CARS* (*P*≤0.05 for all; **Figure 4**), findings that suggest regulation by 1,25(OH)^2^-D. *DGKD* and *DGKH* were upregulated in the tibia in response to high *and* low calcium diet (*P*≤0.05 for all; **Figure 4**). The expression in duodenum of the majority of genes was not modified by dietary calcium, with the exception of *NAP1L4* and *CDKN1C*.

## Discussion

We have identified and replicated one known and six new loci for serum calcium near genes linked to bone metabolism and endocrine control of calcium. Of these, 4 loci (*DGKD*, *GCKR*, *CASR*, and *CYP24A1*) were nominally associated with BMD in the general population. In supporting mouse studies, we demonstrate expression of several of these genes in tibia, and show regulation of gene expression in response to dietary calcium intake. We also demonstrate expression in nephron segments known to regulate calcium homeostasis. Taken together, these results shed new light on the genetics of calcium balance.

The vast majority of total body calcium is bound in the skeleton as hydroxyapatite and other calcium-phosphate complexes [10]. Apart from providing skeletal strength, bone serves as a calcium reservoir to maintain tightly controlled circulating concentrations vital to cellular signaling, muscle contraction and coagulation [10]. However, the genetic basis of the dynamic cross talk that occurs between these compartments is poorly understood. Our results advance our understanding in this area. Eight genes identified in the GWAS are constitutively expressed in bone and are regulated in response to dietary calcium, in particular low calcium diet, whereas no clear change was observed in kidney or duodenum. This bone reactivity in response to dietary calcium intake is consistent with what was recently reported for *CASR* [11]. Further, of the eight genes expressed in bone and regulated in response to dietary calcium, we show that rs1550532 (*DGKD*) and rs1801725 (*CASR*) are associated with BMD in humans, the primary determinant of fracture risk.

The A allele of rs1570669 (*CYP24A1* locus) was associated with reduced BMD at the femoral neck although *CYP24A1* was not found to be expressed in bone in mice experiment, which suggests an indirect role in bone mineralization. This may occur via its documented role in vitamin D metabolism, discussed below, and/or its association with higher PTH concentrations identified in the present analysis.

We observed specific expression patterns of several genes in the mouse nephron: *DGKD*, *DGKH*, *CARS*, *KIAA0564* and *CYP24A1* were primarily transcribed in the proximal tubule, *CASR* expression was mostly localized to the thick ascending limb, whereas *GATA3* was predominantly found in the distal part of the nephron and the collecting duct. This pattern of expression in segments known to be involved in calcium reabsorption suggests a role in renal calcium handling and is consistent with previous exploratory transcriptome analyses in humans and mice [12], [13]. Both *DGKD* and *DGKH* were significantly upregulated in the kidney in response to low calcium diet, suggesting specific involvement of these genes in renal calcium handling.

Several of the newly identified loci harbor genes linked to the hormonal control of serum calcium. First, the association of *CASR* with PTH concentrations is consistent with its known role in PTH signaling. Second, several lines of evidence implicate rs1570669 (*CYP24A1*) in the vitamin D pathway: its association with serum calcium and PTH concentrations, its selective expression in the proximal tubule where 1,25(OH)2-D metabolism occurs, and that loss-of-function *CYP24A1* mutations cause vitamin D-induced hypercalcemia in children (idiopathic infantile hypercalcemia). Third, we identified variants linked to 2 chromosomally distinct isoforms of diacylglycerol kinase, part of the phosphoinositol second messenger system, that may interact with each other at the protein level [14], [15].

Strengths of this study are the large sample size and consistent mouse studies to support the statistical associations and advance our knowledge of the biology at these loci. Human and mice largely share physiological processes linked to calcium metabolism, including tissue-specific gene expression. Limitations include the lack of a direct marker of bone remodeling and the potential for bias in gene selection for experimental follow-up. Mice may display subtle differences in the regulation of the genes tested compared to humans.

We have identified and replicated one known and six new loci for serum calcium near genes linked to bone metabolism and endocrine control of serum calcium. Supporting experimental mouse studies suggest a role for dietary calcium in bone-specific gene expression. Further work is needed to identify the causal variants and to understand how they influence calcium homeostasis.

## Materials and Methods

### Ethics statement

In each human study, the local institutional review board approved the study and participants signed written informed consent, including for DNA analyses. The experimental protocol in mice was approved by the local veterinarian authorities and fulfilled Swiss federal regulations for experiences with animals.

### Participating studies (human data)

#### Discovery and replication cohorts

A list of all discovery and replication studies, their sample size, mean serum calcium levels, age and serum albumin as well as proportion of women can be found in **Table S1**. We replicated findings using *de novo* genotyping in the Bus Santé Study and *in silico* data in all other cohorts. In most studies, serum calcium was measured using a colorimetric assay. The size of discovery tables varied from 488 to 9,049 for a total of 39,400 participants. A detailed description of the characteristics of discovery and replication cohorts, including laboratory method for serum calcium measurement, can be found in **Table S12**.

### Genotyping

Detailed information on the genotyping plateforms and data cleaning procedures for each discovery and replication cohort can be found in **Table S13**. *De novo* replication genotyping was perfomed in 4670 participants to the Bus Santé Study using KASPar v4.0 after whole genome amplification by primer extension pre-amplification (PEP) using thermostable DNA polymerases.

### Statistical analyses for the genome-wide association meta-analysis

In each discovery study, genotyping was performed using a genome-wide chip and nearly 2.5 million SNPs were genotyped or imputed using the HapMap CEU panels release 22 or 21 as the reference. Each study applied quality control before imputation. Detailed imputation information is provided in **Table S13**. Each SNP was modeled using an additive genetic effect (allele dosage for imputed SNPs), including age and sex as covariates in the model as well as study-specific covariates if needed (e.g. principal components, study center). The primary dependent variable in each discovery study was untransformed and uncorrected serum calcium expressed in mg/dL. Beta regression coefficients and standard errors were used with at least 5 decimal places. For secondary analyses, albumin-corrected serum calcium was computed using the following formula: ([4-plasma albumin in g/dL]×0.8+serum calcium in mg/dL) and the same model as for the primary analyses was used. Each file of genome-wide summary statistics underwent extensive quality control prior to meta-analysis both for primary and secondary analyses, including (1) boxplots of all beta coefficients, as well as all standard errors multiplied by the square-root of the sample size, for each study separately; (2) the range of P values, MAF, imputation qualities, call rates and Hardy-Weinberg equilibrium P values and (3) QQ plots. In addition, we checked the direction and magnitude of effect at the previously reported rs1801725 *CASR* variant. Genome-wide meta-analyses were conducted in duplicate by two independent analysts. For each SNP, we used a fixed effect meta-analysis using inverse-variance weights as implemented in the meta-analysis utility Metal [16]. Results were confirmed by a z-score based meta-analysis. Data were available for 2,612,817 genotyped or imputed autosomal SNPs for the primary and secondary analyses. After the meta-analysis, genomic control correction was applied (λGC was 1.03 for both uncorrected and corrected serum calcium). Our pre-specified criterion to declare genome-wide significance was P value<5E-8 to account for 1 million independent tests according to the Bonferroni correction. We choose to move forward for replication all SNPs with discovery P value<1E-7 in the European sample or genome-wide significant SNP in the overall sample that included Indian Asians. To choose a single SNP per genome-wide associated region for replication, we merged all SNPs within 1 Mb region and selected the lowest P value for each region. Altogether, fourteen SNPs were moved forward for replication. Up to 17,205 participants contributed information to the replication analyses *in silico* and 4,670 participants provided data for *de novo* genotyping. We used fixed-effects inverse-variance weighted meta-analysis to combine discovery and replication meta-analysis results. Replication was considered as present whenever a combined P value<5E-8 together with an effect-concordant one-sided replication P value<0.05 were obtained.

### Data for look-ups of serum calcium loci with related phenotypes

We conducted look-ups for femoral and lumbar bone density in the GEnetic Factors of OSteoporosis (GEFOS) dataset [17]. Bone mineral density (BMD) is used in clinical practice for the diagnosis of osteoporosis and bone density at different skeletal sites is predictive of fracture risk. BMD was measured in all cohorts at the lumbar spine (either at L1–L4 or L2–L4) and femoral neck using dual-energy X-ray absorptiometry following standard manufacturer protocols [17]. Serum phosphorus was looked up from a previously published GWAS meta-analysis, including 16,264 participants of European ancestry [18]. Serum phosphorus concentrations were quantified using an automated platform in which inorganic phosphorus reacts with ammonium molybdate in an acidic solution to form a colored phosphomolybdate complex [18]. The 25-hydroxyvitamin D was looked-up in the SUNLIGHT consortium [7], which includes data from 33,996 individuals of European descent from 15 cohorts. 25-hydroxyvitamin D concentrations were measured by radioimmunoassay, chemiluminescent assay, ELISA, or mass spectrometry [7]. PTH was looked-up in the SHIP and SHIP-Trend studies. The serum parathyroid hormone concentration was measured on the IDS-iSYS Multi-Discipline Automated Analyser with the IDS-iSYS Intact PTH assay (Immunodiagnostic Systems Limited, Frankfurt am Main, Germany) according to the instructions for use. This chemiluminescence immunoassay detects the full-length parathyroid hormone (amino acids 1–84) and the large parathyroid hormone fragment (amino acids 7–84). The measurement range of the assay was 5–5000 pg/mL. The limits of blank, detection and quantitation were 1.3 pg/mL, 1.4 pg/mL, and 3.6 pg/mL, respectively. As recommended by the manufacturer, three levels of control material were measured in order to verify a decent working mode. During the course of the study, the coefficients of variation were 14.02% at low, 6.64% at medium, and 6.84% at high serum parathyroid hormone concentrations in the control material in SHIP and the corresponding percentages were 16.8% at low, 10.7% at medium, and 9.0% at high serum parathyroid hormone concentrations in the control material in SHIP-Trend.

### Copy Number Variation (CNV) analysis

The Hypergene dataset (a 4206 samples case-control study concerning hypertension genotyped using the Illumina 1M chip) has been used to call CNVs and to check their correlation with the SNPs of interest. The CNVs calls have been done using pennCNV software [19]. A SNP by sample matrix with the copy number status was created. Then the square correlation (Pearson correlation) between value of each SNP of interest and the SNPs copy number status in a +/−2 Mb region was calculated. The SNPs of interest for which no correspondence has been found in the Hypergene dataset have been replaced by the closest SNPs in high linkage disequilibrium (LD) and present in the Hypergene dataset. LD between the SNPs of interest and a list of SNPs tagging CNVs from the GIANT consortium has also been calculated. The SNPs from the GIANT list are in LD higher than 0.8 with their corresponding CNV.

### Gene ontology classification analysis

We queried the AmiGo 1.8 gene ontology database for each gene located within ±250 kb of the seven replicated SNPs, including rs1801725 (*CASR*). (http://amigo.geneontology.org/cgi-bin/amigo/go.cgi, last accessed November 6, 2012). We used *Homo sapiens* as a filter for species.

### Expression quantitative trait locus (eQTL) Analyses

For each of the 7 replicated SNPs, we identified all proxy SNPs with r^2^>0.8 in HapMap CEU (releases 21, 22, and HapMap 3 vers. 2) using the online SNAP database (http://www.broadinstitute.org/mpg/snap/). We then queried each of these 40 SNPs in the eQTL database of the University of Chicago (http://eqtl.uchicago.edu/cgi-bin/gbrowse/eqtl/).

### Rationale for gene selection for experimental analyses in mouse

The rs1801725 SNP encodes a missense variant in exon 7 of the CASR gene leading to an alanine to serine substitution (A986S). Given the key physiological role of CASR in calcium homeostasis (monogenic disorders of calcium balance), this gene was the logical candidate for analysis in mouse at this previously identified locus.

For the 6 newly identified loci, the precise rationale for gene selection varied from one locus to the other, but the main criteria was to focus on the most biologically relevant gene. Rs1550532 on chromosome 2 is an intronic SNP of *DGKD*, which was the most likely biological candidate for this locus and was therefore selected for analysis in mouse. None of the other genes located in this region (±250 Kb) has a known link with calcium homeostasis (**Box 1**) and rs1550532 is not in strong linkage disequilibrium with an eQTL (**Table S6**). We also took into account the fact that another member of the DGK family, namely DGKH was located near one of the other replicated loci, on chromosome 13.

Rs780094, on chromosome 2, is located in intro 16 of *GCKR* and is in strong linkage disequilibrium (r^2^ = 0.93) in Caucasians [20], with a common non-synonymous SNP (P446L, rs1260326) associated with glucokinase activity *in vitro* [20], [21]. This SNP has been associated with multiple other phenotypes in previous GWAS and it is in strong linkage disequilibrium with an eQTL (**Table S6**). Previous fine mapping analysis of this locus has attributed the signal from rs780094 to the functional rs1260326 variant [20]. *The GCKR* locus may indirectly influence calcium concentrations via its association with albumin levels [22]. In line with this, we observed an attenuation of the association of rs780094 with albumin-corrected serum calcium compared to the association with uncorrected serum calcium and we found *GCKR* not to be expressed in any of the key organs involved in calcium homeostasis that we tested in mice. We selected *GCKR* for analysis in mouse at this locus.

Rs10491003 on chromosome 10 is located within a long non-coding RNA. For this locus, we selected *GATA3*, the nearest and only gene located within this region, for analysis in mouse. GATA3 is implicated in monogenic disorders of calcium balance.

Rs7481584 is located within *CARS* (intronic SNP) in an imprinted region known to play a role in multiple cancers, which makes this locus a plausible candidate for malignancy-related hypercalcemia. Other plausible biological candidates in this locus are *NAP1L4, PHLDA2 and CKDN1C* (**Box 1**). Rs7481584 is in strong LD with 2 eQTLs, one associated with the expression of *NAP1L4* (rs2583435) and the other one associated with the expressions of *SLC22A18* and *SLC22A18AS*. We selected *CARS*, *NAP1L4, PHLDA2 and CKDN1C* for analyses in mouse.

For rs7336933, we selected the two only genes (*DGKH* and *KIAA0564*) located under this association peak on chromosome 13 for analyses in mouse.

Finally, rs1570669 is an intronic SNP of *CYP24A1*, a strong biological candidate implicated in monogenic disorders of calcium balance. The two other genes of this region (*BCAS1* and *PFDN4*) have no known link with calcium homeostasis. Furthermore, rs1570669 and *PFDN4* are separated by a recombination hot spot. We selected *CYP24A1* for analysis in mouse.

As animal experiments started while the replication process was underway, we had also initially selected the following genes for analysis in mouse: RSG14 and *SLC34A1* at locus rs4074995 (discovery P value = 2.4E-07), *VKORC1L1* at locus rs17711722 (discovery P value = 2.8E-11), *PYGB* at locus rs2281558 (discovery P value = 6.4E-07), *CD109* at locus rs9447004 (discovery P value = 8.1E-06). No gene was selected for the rs2885836 and rs11967485 and rs12150338 loci in the absence of obvious candidate. Results for these unreplicated loci can be found in **Figures S6, S7 and S8**. We present these results for quality control purposes: *SLC34A1* (also known as NAPI-3 or NPT2), which encodes solute carrier family 34 (sodium phosphate), member 1, was expressed in the kidney, but neither in duodenum nor in bone, as expected based on current knowledge on this phosphate transporter. In the kidney *SLC34A1* was mainly expressed proximally and *SLC34A1* expression was upregulated under low calcium diet, which is in line with the known function of this gene.

### Mouse experiments

Five C57bl/6 mice (Janvier) per group were fed, for one week, three different diets in which the percentage of calcium were 0.17% (low calcium diet), 0.82% (normal calcium diet) and 1.69% (high calcium diet) and had free access to water. 12∶12 hours light/dark alternance was imposed. At the end of the week of the specific diet, spot urine were collected and mice were anesthetized. Blood was collected by retro-orbital puncture. Organs were immediately harvested and snap frozen. RNA was extracted using Trizol (Invitrogen) and reversed transcribed with PrimeScriptTM RT reagent Kit (Takara Bio Inc). Calcium, sodium, phosphate and creatinine in plasma and urine were analyzed at the central lab of the Lausanne University hospital using a Cobas-Mira analyzer (Roche).

#### Microdissection

A separate set of three mice was kept under normal calcium diet. Proximal Tubule (Prox), thick ascending limb of the loop of Henle (TAL), distal convoluted tubule and connecting tubule (DCT-CNT) and cortical collecting duct (CCD) were isolated by microdissection of the left kidney after the mice were perfused with Liberase TM (Roche Diagnostics) [23]. RNA was extracted from the above mentioned tubules following TRI Reagent Solution protocol (Applied Biosystems) and purified with RNeasy Micro Kit (Qiagen). Reversed transcription was performed with PrimeScriptTM RT reagent Kit (Takara Bio Inc). Quantitative PCRs were performed (7500 Software v 2.0.4.) using TaqMan gene expression assays for the different genes (Applied Biosystems) and comparative CT method was applied. Expression levels were normalised to beta actin as endogenous reference gene.

#### Statistics

Comparison of groups was performed using unpaired Student's t-test.

## Supporting Information

Figure S1 QQ-plot of uncorrected serum calcium GWAS meta-analysis. Quantile-quantile plot showing observed p-values of the uncorrected serum calcium meta-analysis vs. expected p values by chance. The second genomic control step was applied to correct for the post meta-analysis of λ = 1.03.
(PDF)

Figure S2 Regional association plot for the CASR locus. Regional association plot showing −log10 p-values for the association of all SNPs ordered by their chromosomal position with uncorrected serum calcium at the *CASR* loci. The −log10 P value for each SNP is colored according to the correlation of the corresponding SNP with the SNP showing the lowest p-value (index SNP) within the locus using different colors for selected levels of linkage disequilibrium (r^2^). Correlation structures correspond to HapMap 2 CEU.
(PDF)

Figure S3 Regional association plot for the newly identified loci. Regional association plot showing −log10 p-values for the association of all SNPs ordered by their chromosomal position with uncorrected serum calcium within the replicated loci. The −log10 P value for each SNP is colored according to the correlation of the corresponding SNP with the SNP showing the lowest p-value (index SNP) within the locus using different colors for selected levels of linkage disequilibrium (r^2^). Correlation structures correspond to HapMap 2 CEU.
(PDF)

Figure S4 Manhattan plot of corrected serum calcium. Manhattan plot showing −log10 (P values) for all SNPs analyzed, ordered by their chromosomal position. The values correspond to the association of albumin-corrected serum calcium, including age and sex as covariates in the model as well as study-specific covariates if needed.
(PDF)

Figure S5 QQ-plot of corrected serum calcium. Quantile-quantile plot showing observed p-values of the corrected serum calcium meta-analysis vs. expected P values by chance in Europeans at discovery. The second genomic control step was applied to correct for the post meta-analysis of λ = 1.03.
(PDF)

Figure S6 Relative expression of genes in non-replicated loci in kidney, duodenum and tibia. The expression (based on delta CT normalized to actin) of the selected genes is compared to the expression of the *CASR* gene in the duodenum, thereby providing a relative expression. Cut-off was set at delta CT≤15. Data are means ± SEM of values obtained from 5 mice fed a normal diet.
(PDF)

Figure S7 Relative expression in segments of kidney tubules of genes located in non-replication loci. The renal tubular segments analyzed were the proximal tubule (PROX), the thick ascending limb of the loop of Henle (TAL), the distal convoluted tubule and connecting tubule (DCT-CNT), and the cortical collecting duct (CCD). The expression (based on the delta CT) of the selected genes is compared to the expression of the CASR gene in the PROX. Data are means of values obtained from 3 mice fed a normal diet. *GCKR* was not expressed.
(PDF)

Figure S8 Relative expression of genes in non-replicated loci under various calcium diets. Data are means± SEM of values obtained from 5 mice fed a low (0.17%) and high (1.69%) calcium diet compared to mice fed a normal calcium diet (0.82%). Expression levels were normalized to actin. Statistical difference was calculated using unpaired t-test. *: P value≤0.05 (low compared to high); §: P value≤0.05 (low compared to normal); #: P value≤0.05 (high compared to normal).
(PDF)

Table S1 Characteristics of study participants in discovery and replication cohorts. Data are mean (SD) unless otherwise specified for each discovery and replication studies.
(DOCX)

Table S2 SNPs brought forward for replication that did not replicate. Chr, chromosome. A1, effect allele. A2, non-effect allele. Effect A1, regression coefficient for the A1 allele. SE, standard error. Freq A1,frequency of allele A1.
(DOCX)

Table S3 SNPs with P value<5*E-05 for uncorrected calcium in Europeans (discovery). Chr, chromosome. Position, position on build 36. A1, allele 1 (effect allele). A2, allele 2. Freq A1, frequency of allele 1. InRefGen, gene symbol if SNP is located within a specific gene.
(DOCX)

Table S4 Comparison of association with uncorrected versus corrected serum calcium. Chr, chromosome. Freq A1, frequency of allele A1. Beta, regression coefficient for the A1 allele. SE, standard error. A1, allele 1 (effect allele). Only replicated loci are included in this table.
(DOCX)

Table S5 Genome-wide significant loci for corrected calcium in Europeans (discovery). Chr, chromosome. Position, position on build 36. A1, allele 1 (effect allele). A2, allele 2. Freq A1, frequency of allele 1. InRefGen, gene symbol if SNP is located within a specific gene.
(DOCX)

Table S6 eQTL analysis for the seven genome-wide replicated loci for serum calcium. We used the online eQTL database of the University of Chicago (http://eqtl.uchicago.edu/cgi-bin/gbrowse/eqtl/., last accessed, November 5, 2012). All eQTL were acting in *cis*.
(DOCX)

Table S7 Details on genes located in the GCKR genomic region.
(DOCX)

Table S8 Gene Ontology classification (AmiGo). Data are GO numbers, ontology and mechanism/location from the AmiGo 1.8 gene ontology database for each gene located within ±250 kb of the seven replicated SNPs, including rs1801725 (CASR).
(DOCX)

Table S9 OMIM disorders associated with the genes located within the replicated loci. This table includes all Mendelian disorders or other types of genetic disorders included in the OMIM database described for each gene located within ±250 kb of any of the six new loci and for *CASR*.
(DOCX)

Table S10 Association of replicated serum calcium loci in other ethnic groups. Chr, chromosome. Position, position on build 36. A1, allele 1 (effect allele). A2, allele 2. Freq A1, frequency of allele 1. Effect A1, regression coefficient for the A1 allele. SE, standard error. NA, not available.
(DOCX)

Table S11 Plasma and Urine electrolytes values by calcium diet in mice. Data are means ± SEM of values obtained from 3 to 5 mice. *: P value≤0.05 compared to normal or high calcium diet.
(DOCX)

Table S12 Study information.
(DOCX)

Table S13 Genotyping information for each cohort (discovery, replication and look-ups).
(DOCX)

Text S1 Study specific acknowledgements.
(DOCX)
