# Supplementary figures and images for: Meta-Analysis of Genome-Wide Association Studies Identifies Six New Loci for Serum Calcium Concentrations
Source: PLoS Genet. 2013 Sep 19;9(9):e1003796. doi: 10.1371/journal.pgen.1003796 (PMC3778004; doi:10.1371/journal.pgen.1003796)

SUPPLEMENTARY FIGURE 1: QQ-PLOT OF UNCORRECTED SERUM CALCIUM GWAS META-ANALYSIS

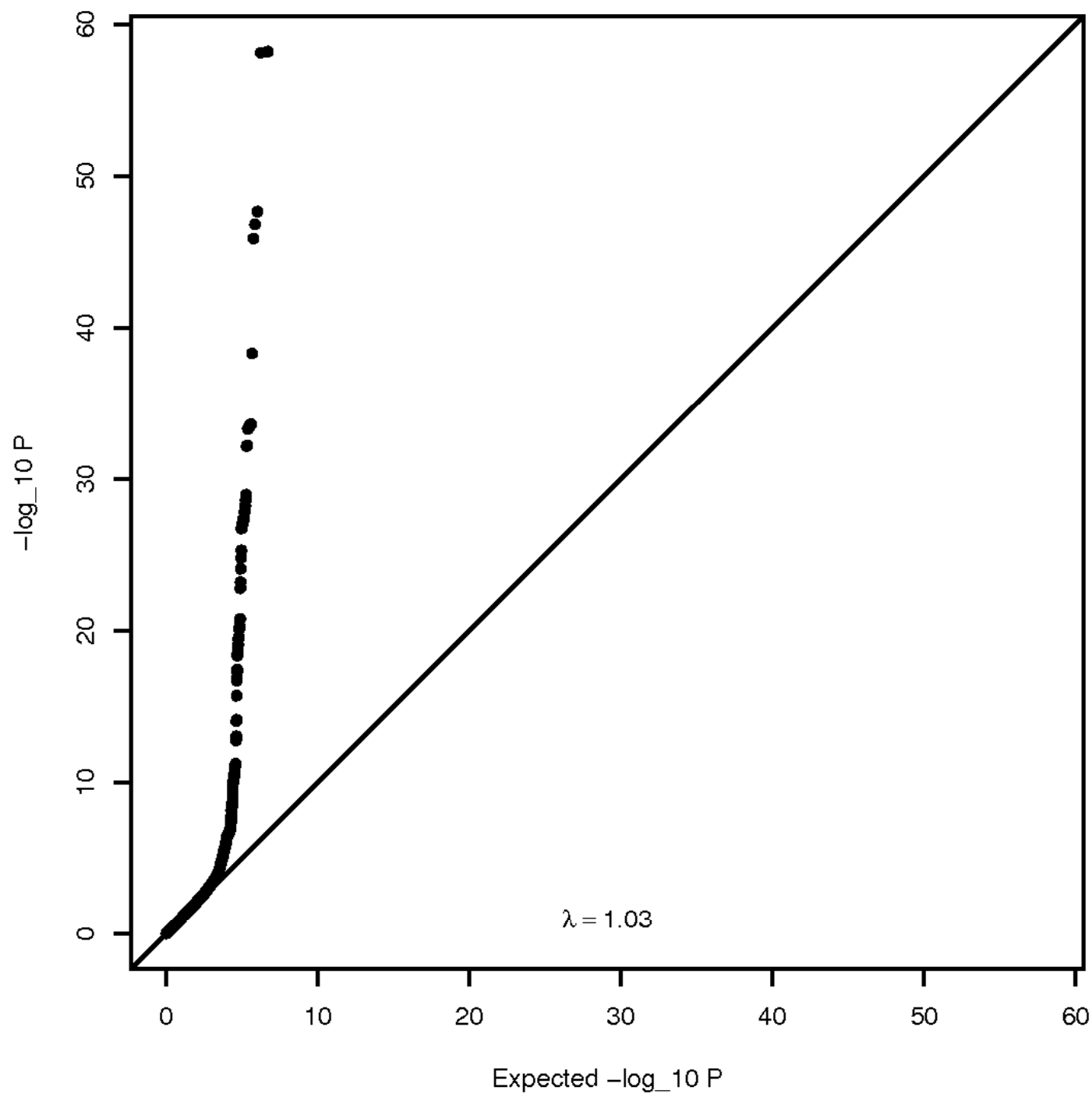

Supplement: Figure S1 — QQ-plot of uncorrected serum calcium GWAS meta-analysis. Quantile-quantile plot showing observed p-values of the uncorrected serum calcium meta-analysis vs. expected p values by chance. The second genomic control step was applied to correct for the post meta-analysis of λ = 1.03. (PDF) [file pgen.1003796.s001.pdf]

SUPPLEMENTARY FIGURE 2: REGIONAL ASSOCIATION PLOT FOR THE CASR LOCUS

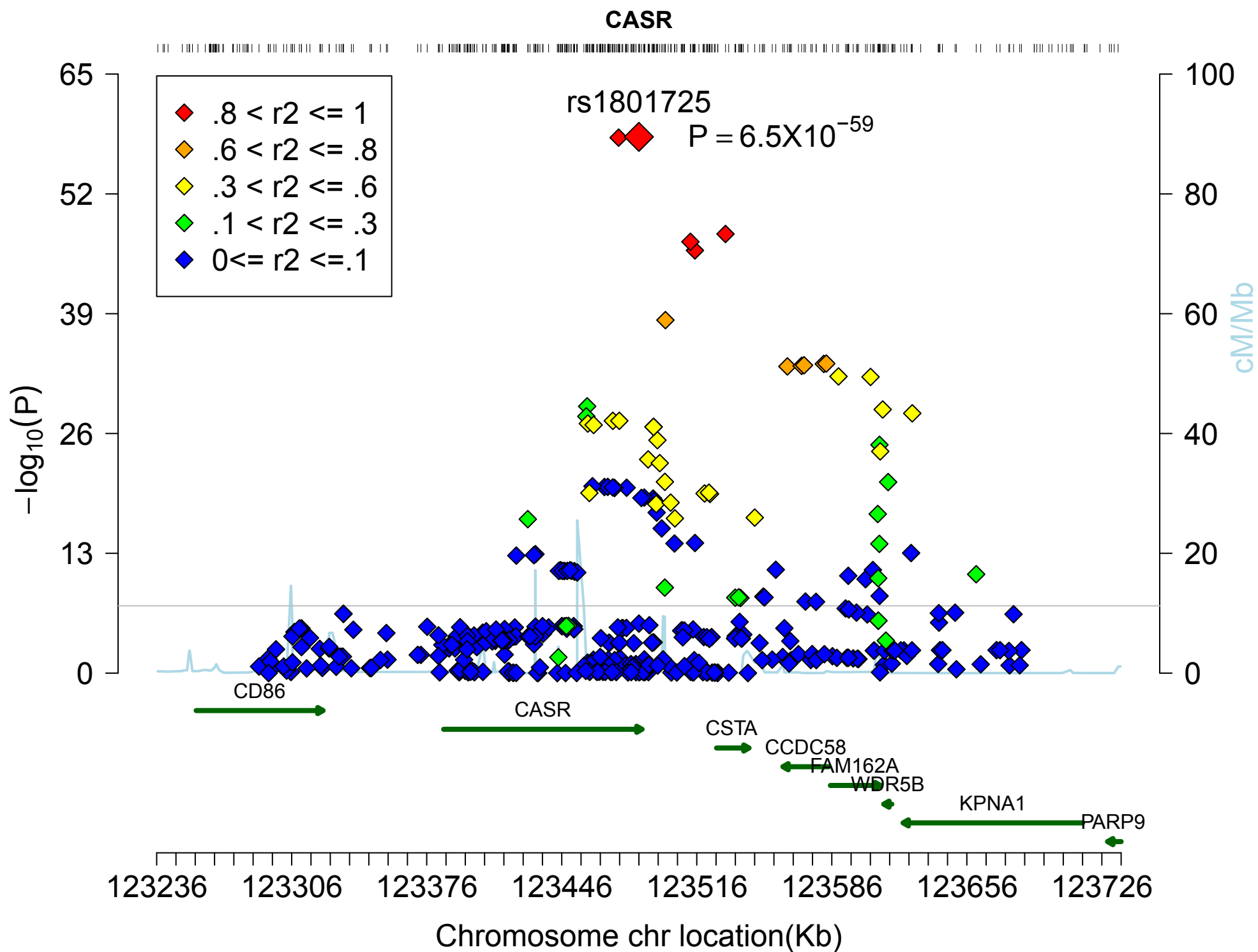

Supplement: Figure S2 — Regional association plot for the CASR locus. Regional association plot showing −log10 p-values for the association of all SNPs ordered by their chromosomal position with uncorrected serum calcium at the CASR loci. The −log10 P value for each SNP is colored according to the correlation of the corresponding SNP with the SNP showing the lowest p-value (index SNP) within the locus using different colors for selected levels of linkage disequilibrium (r2). Correlation structures correspond to HapMap 2 CEU. (PDF) [file pgen.1003796.s002.pdf]

**SUPPLEMENTARY FIGURE 3: REGIONAL ASSOCIATION PLOT FOR THE NEWLY IDENTIFIED LOCI**

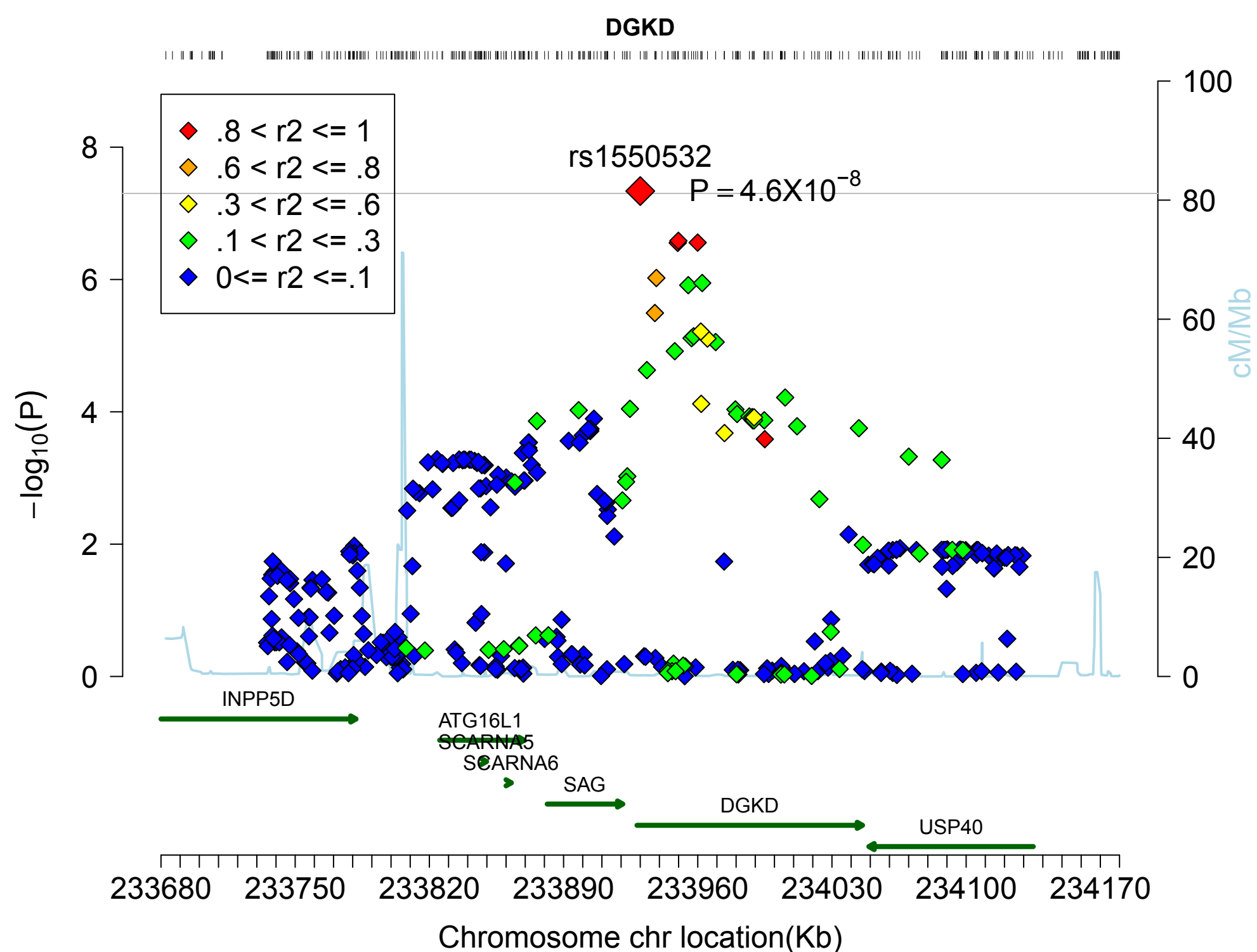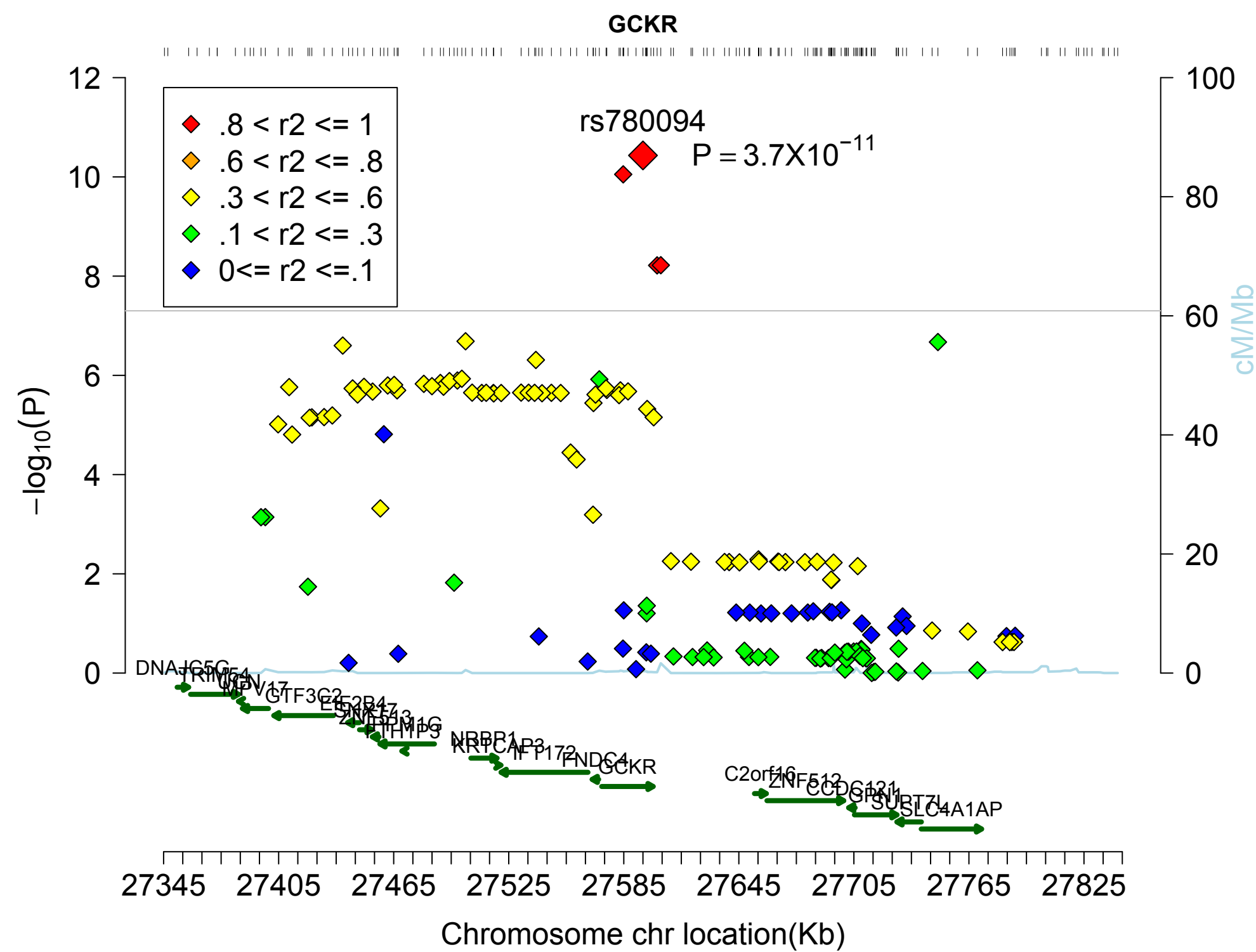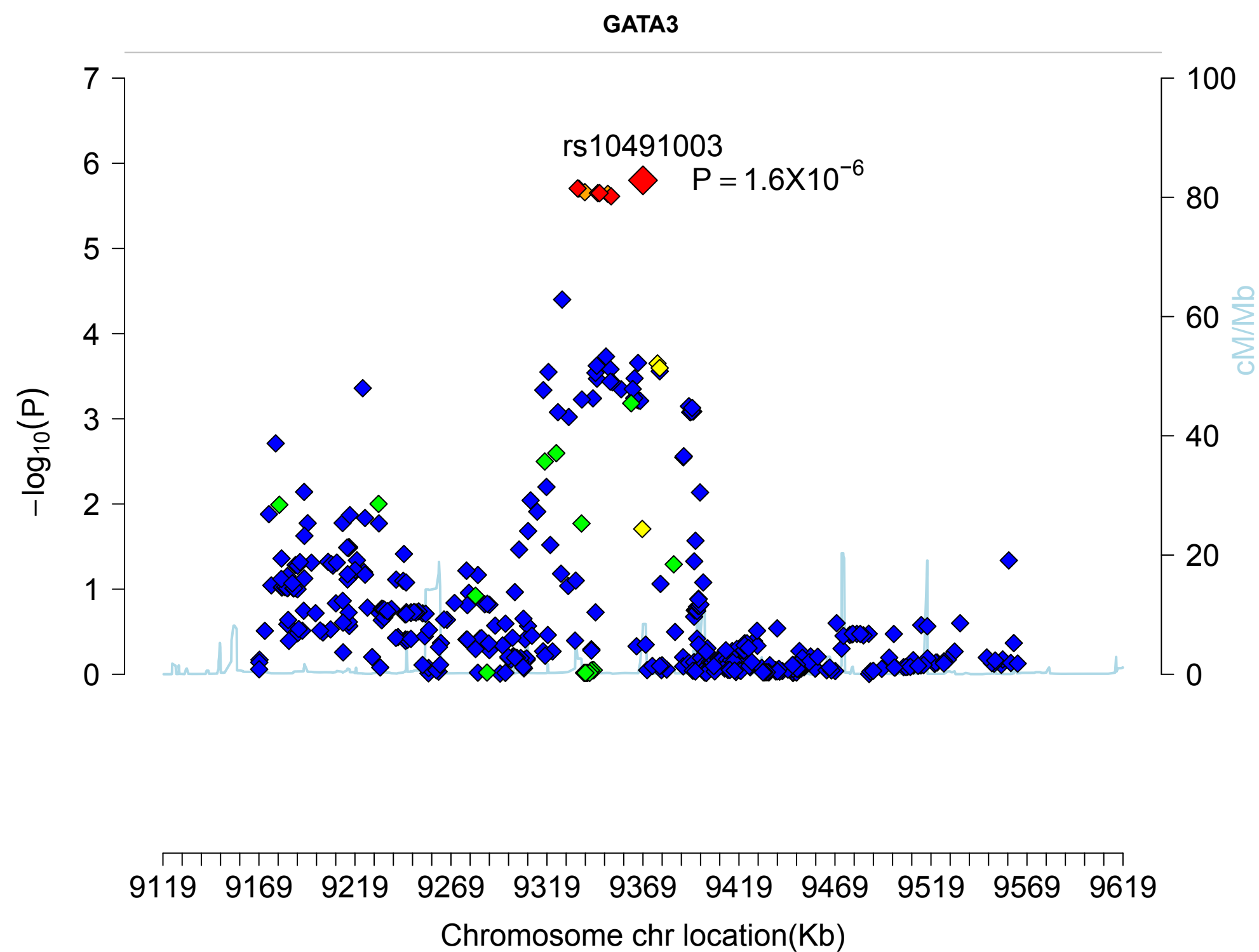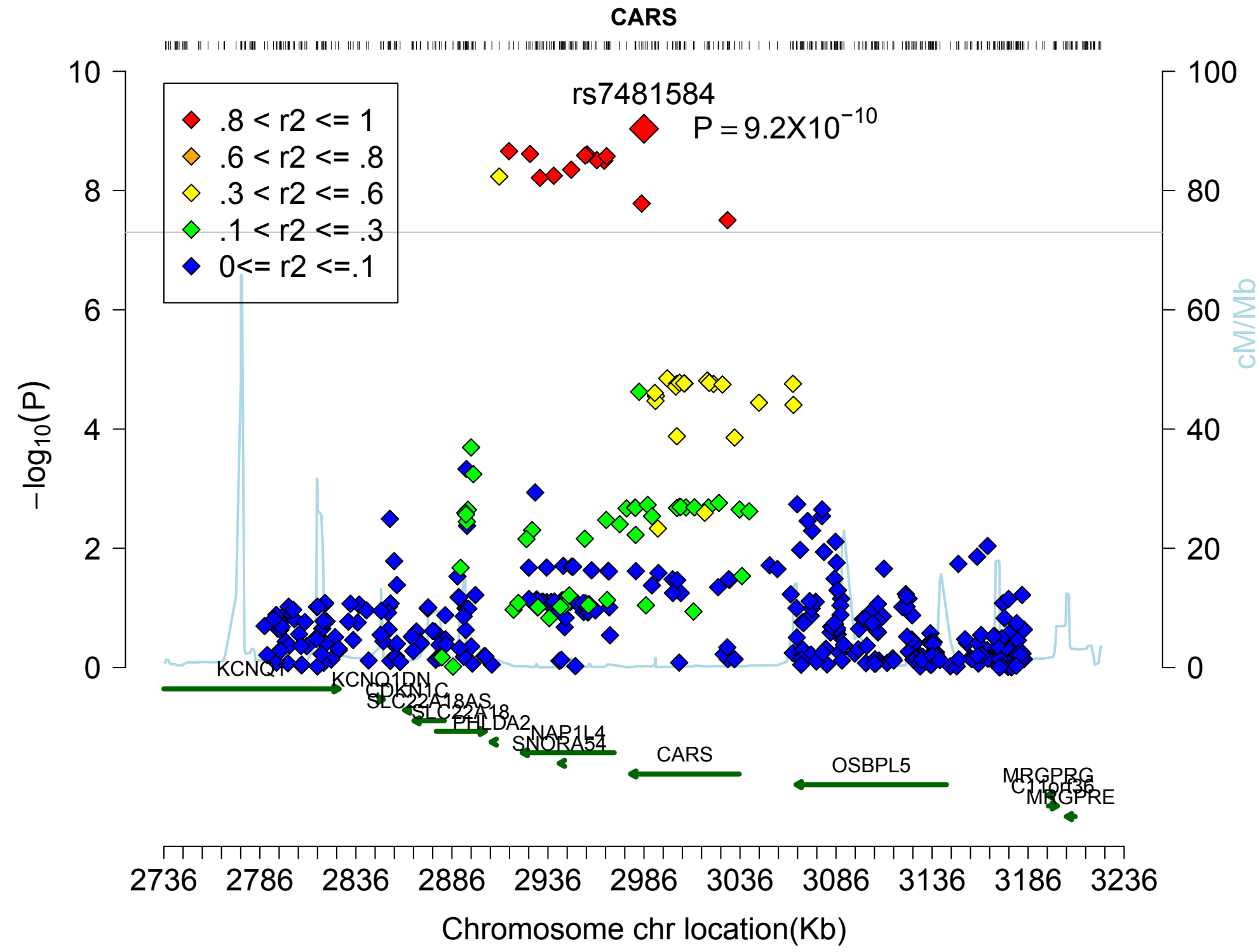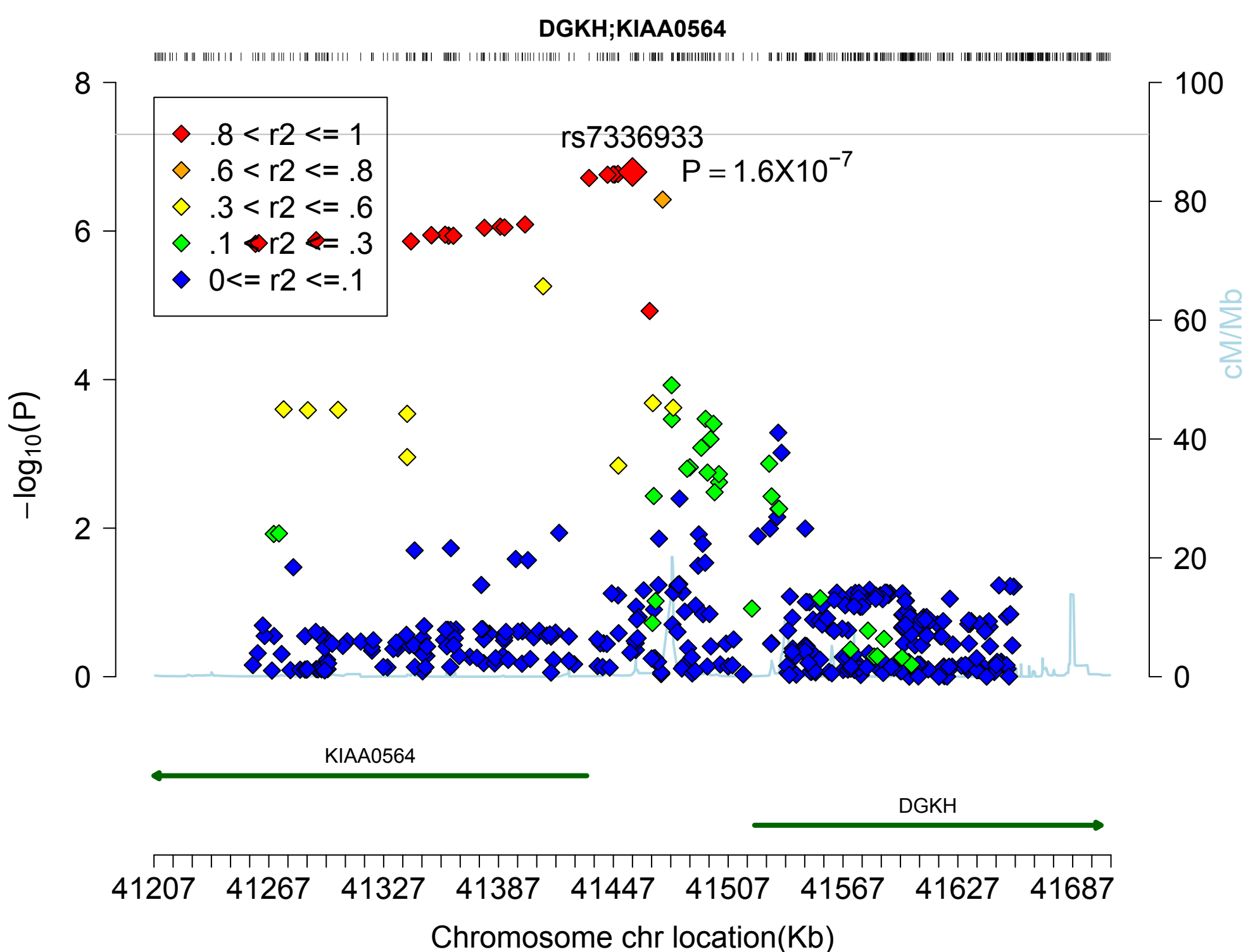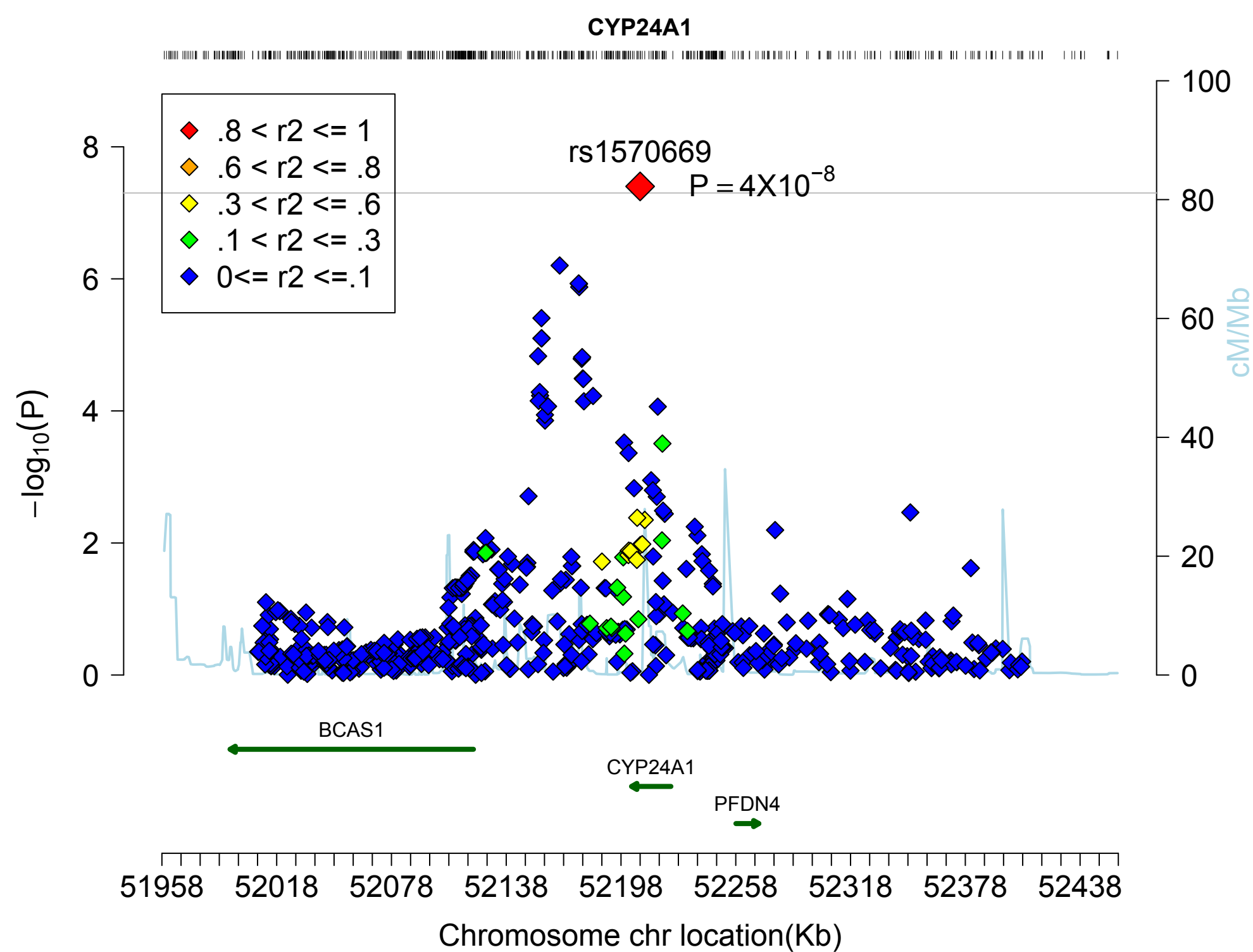

Supplement: Figure S3 — Regional association plot for the newly identified loci. Regional association plot showing −log10 p-values for the association of all SNPs ordered by their chromosomal position with uncorrected serum calcium within the replicated loci. The −log10 P value for each SNP is colored according to the correlation of the corresponding SNP with the SNP showing the lowest p-value (index SNP) within the locus using different colors for selected levels of linkage disequilibrium (r2). Correlation structures correspond to HapMap 2 CEU. (PDF) [file pgen.1003796.s003.pdf]

SUPPLEMENTARY FIGURE 4: MANHATTAN PLOT OF CORRECTED SERUM CALCIUM GWAS META-ANALYSIS

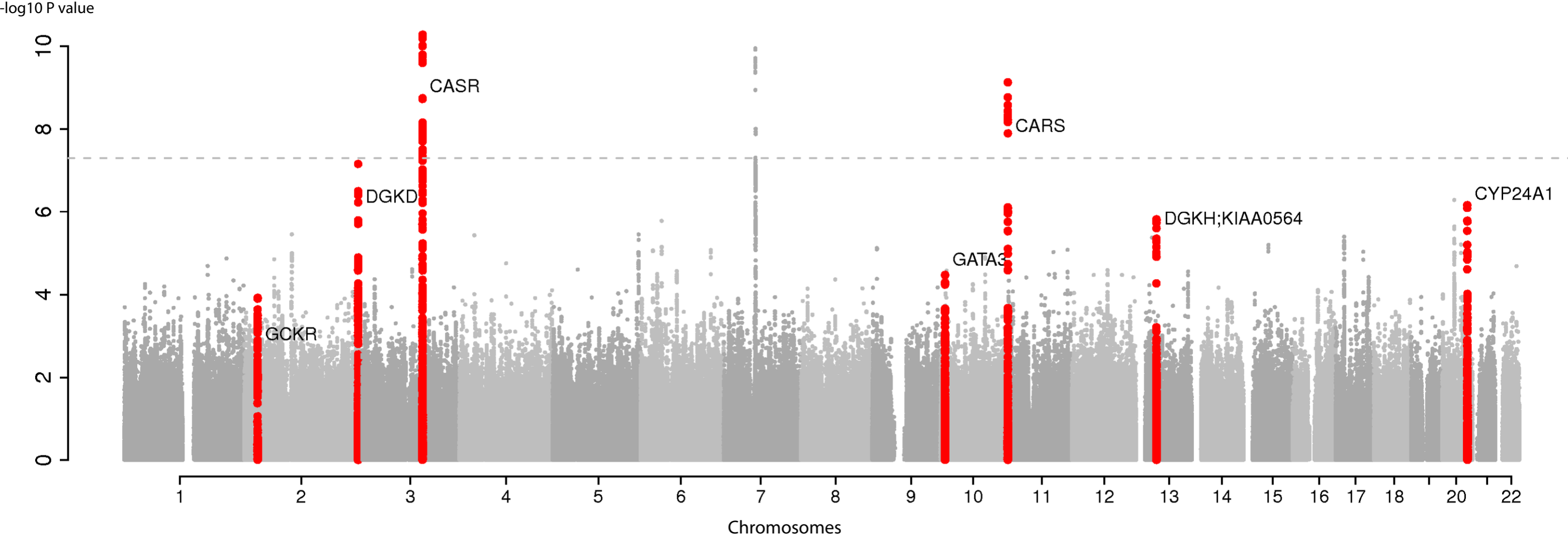

Supplement: Figure S4 — Manhattan plot of corrected serum calcium. Manhattan plot showing −log10 (P values) for all SNPs analyzed, ordered by their chromosomal position. The values correspond to the association of albumin-corrected serum calcium, including age and sex as covariates in the model as well as study-specific covariates if needed. (PDF) [file pgen.1003796.s004.pdf]

SUPPLEMENTARY FIGURE 5: QQ-PLOT OF CORRECTED SERUM CALCIUM

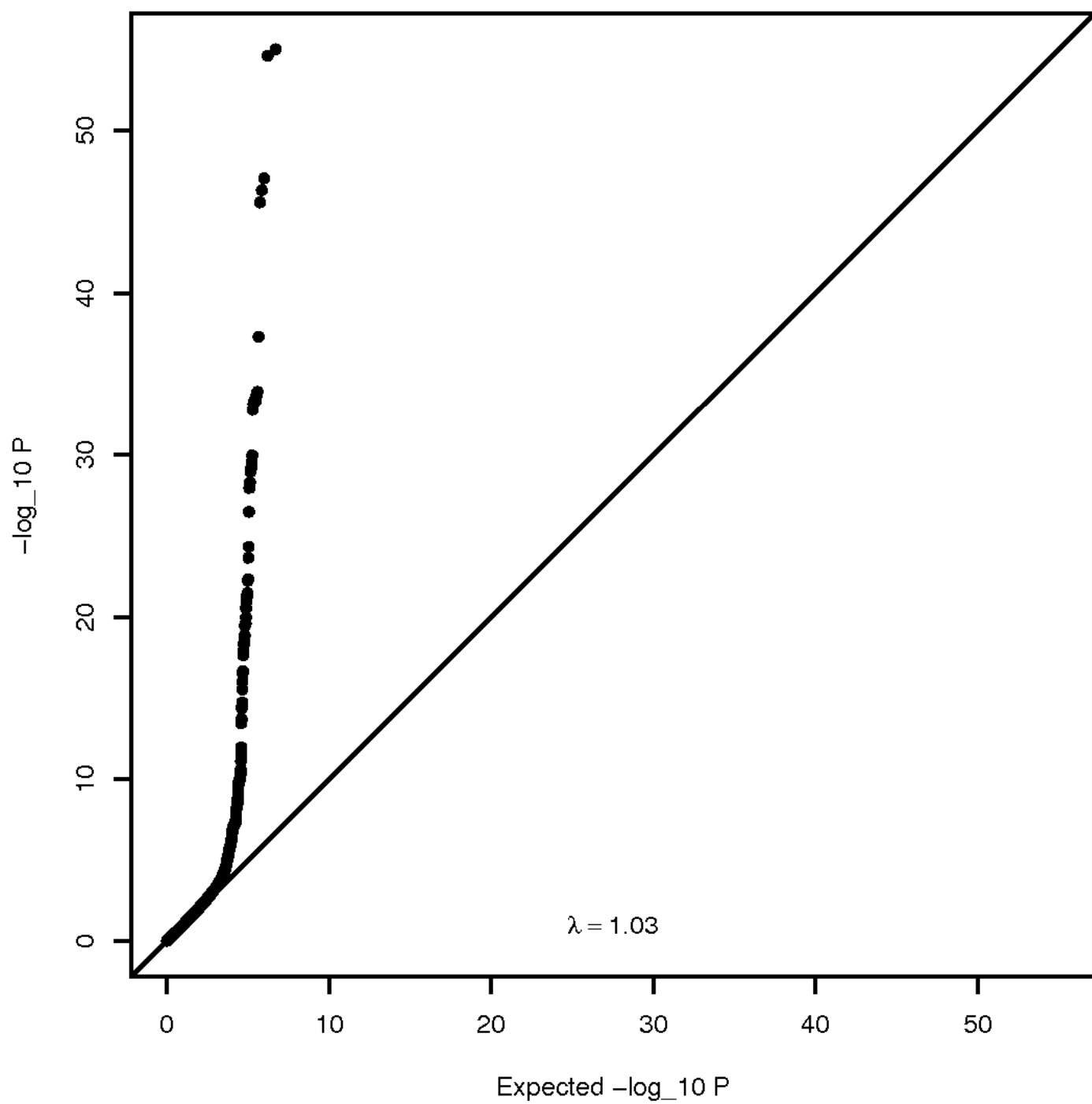

Supplement: Figure S5 — QQ-plot of corrected serum calcium. Quantile-quantile plot showing observed p-values of the corrected serum calcium meta-analysis vs. expected P values by chance in Europeans at discovery. The second genomic control step was applied to correct for the post meta-analysis of λ = 1.03. (PDF) [file pgen.1003796.s005.pdf]
