# Supplementary material for: Meta-Analysis of Genome-Wide Association Studies Identifies Six New Loci for Serum Calcium Concentrations
Source: PLoS Genet. 2013 Sep 19;9(9):e1003796. doi: 10.1371/journal.pgen.1003796 (PMC3778004; doi:10.1371/journal.pgen.1003796)

SUPPLEMENTARY FIGURE 6: RELATIVE mRNA EXPRESSION OF GENES IN NON-REPLICATED LOCI IN KIDNEY, DUODENUM AND TIBIA.

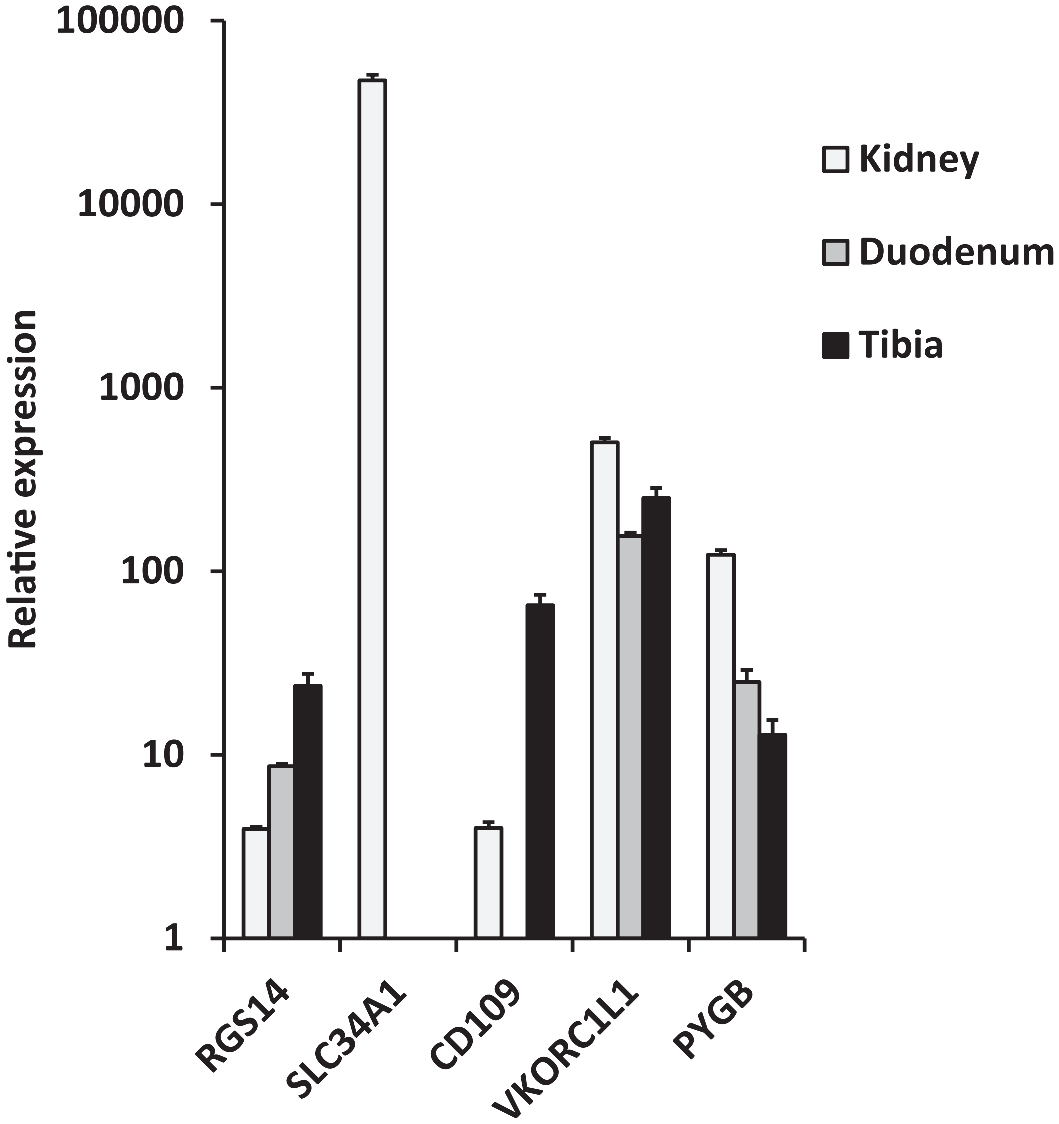

Supplement: Figure S6 — Relative expression of genes in non-replicated loci in kidney, duodenum and tibia. The expression (based on delta CT normalized to actin) of the selected genes is compared to the expression of the CASR gene in the duodenum, thereby providing a relative expression. Cut-off was set at delta CT≤15. Data are means ± SEM of values obtained from 5 mice fed a normal diet. (PDF) [file pgen.1003796.s006.pdf]
