# Supplementary material for: Meta-Analysis of Genome-Wide Association Studies Identifies Six New Loci for Serum Calcium Concentrations
Source: PLoS Genet. 2013 Sep 19;9(9):e1003796. doi: 10.1371/journal.pgen.1003796 (PMC3778004; doi:10.1371/journal.pgen.1003796)

SUPPLEMENTARY FIGURE 7: RELATIVE mRNA EXPRESSION IN SEGMENT OF KIDNEY TUBULES OF GENES LOCATED IN NON-REPLICATION LOCI.

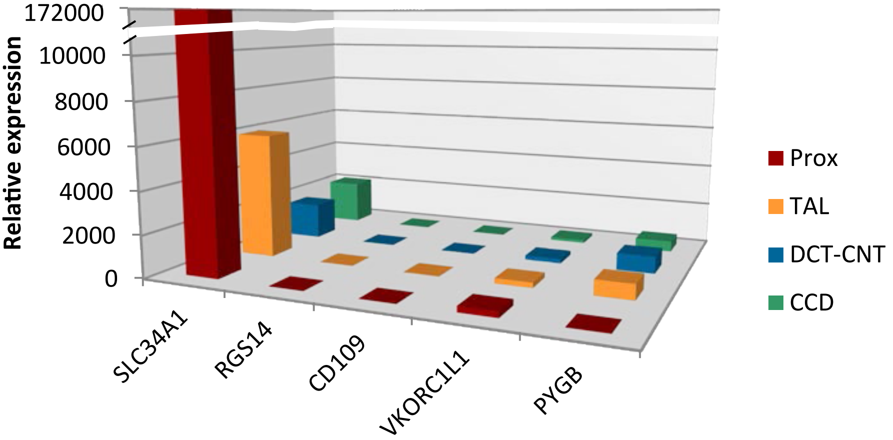

SUPPLEMENTARY FIGURE 8: RELATIVE EXPRESSION OF GENES IN NON-REPLICATED LOCI UNDER VARIOUS CALCIUM DIETS.

Data are means± SEM of values obtained from 5 mice fed a low (0.17%) and high (1.69%) calcium diet compared to mice fed a normal calcium diet (0.82%). Expression levels were normalized to actin. Statistical difference was calculated using unpaired t-test.
*: p≤0.05 (low compared to high);
§: p≤0.05 (low compared to normal);
#: p≤0.05 (high compared to normal).

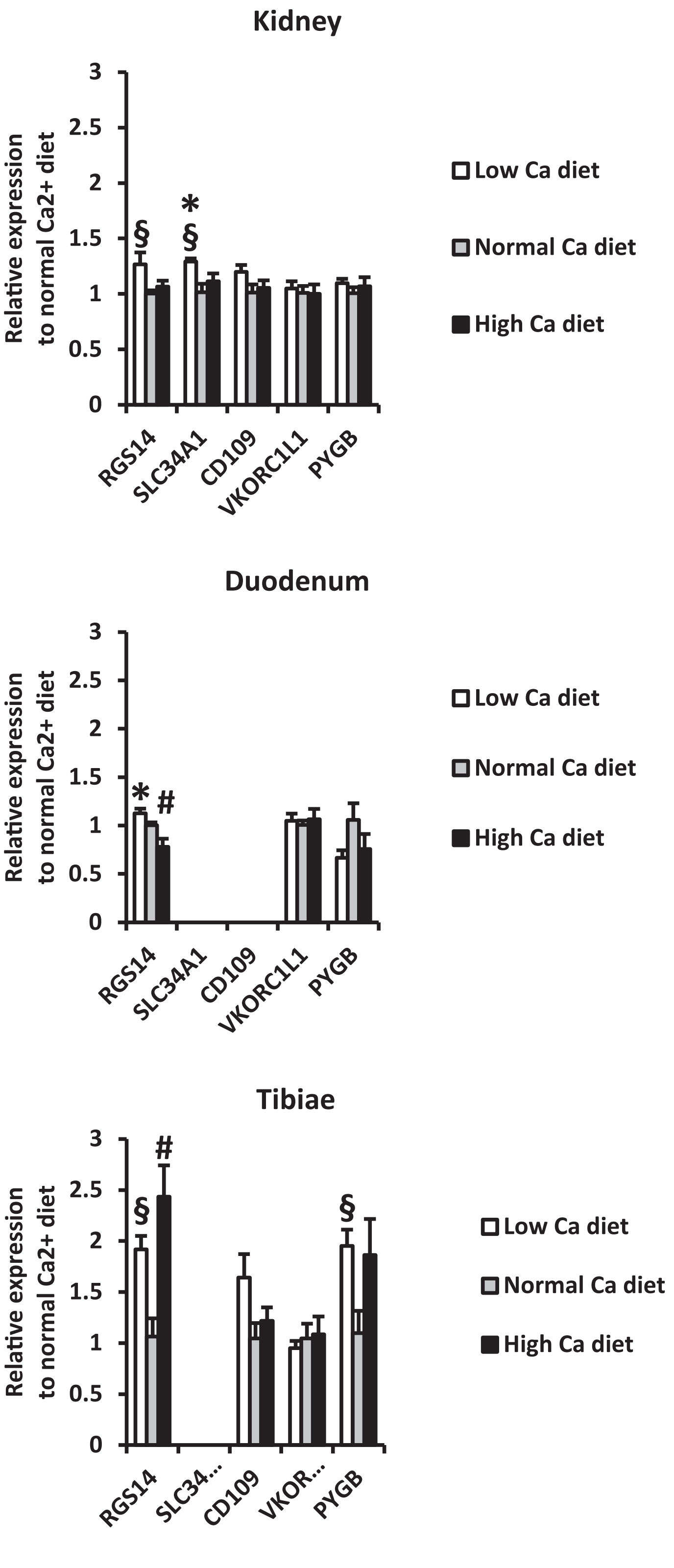

Supplement: Figure S7 — Relative expression in segments of kidney tubules of genes located in non-replication loci. The renal tubular segments analyzed were the proximal tubule (PROX), the thick ascending limb of the loop of Henle (TAL), the distal convoluted tubule and connecting tubule (DCT-CNT), and the cortical collecting duct (CCD). The expression (based on the delta CT) of the selected genes is compared to the expression of the CASR gene in the PROX. Data are means of values obtained from 3 mice fed a normal diet. GCKR was not expressed. (PDF) [file pgen.1003796.s007.pdf]
