# Supplementary material for: Meta-Analysis of Genome-Wide Association Studies Identifies Six New Loci for Serum Calcium Concentrations
Source: PLoS Genet. 2013 Sep 19;9(9):e1003796. doi: 10.1371/journal.pgen.1003796 (PMC3778004; doi:10.1371/journal.pgen.1003796)

SUPPLEMENTARY FIGURE 8: RELATIVE EXPRESSION OF GENES IN NON-REPLICATED LOCI UNDER VARIOUS CALCIUM DIETS.

§: p≤0.05 (low compared to normal);
#: p≤0.05 (high compared to normal).

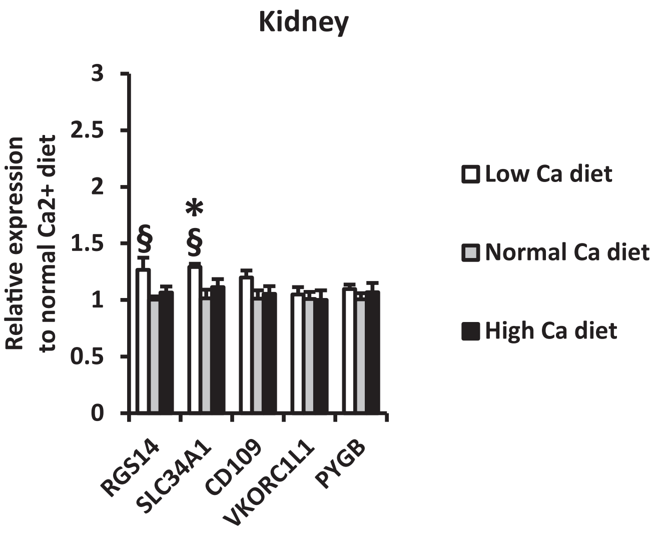

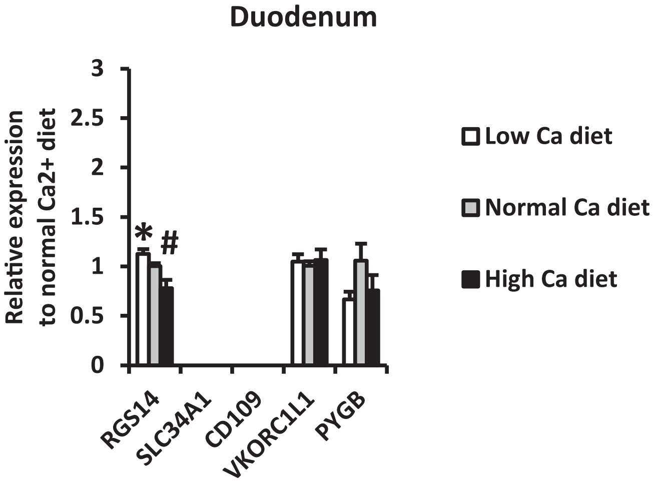

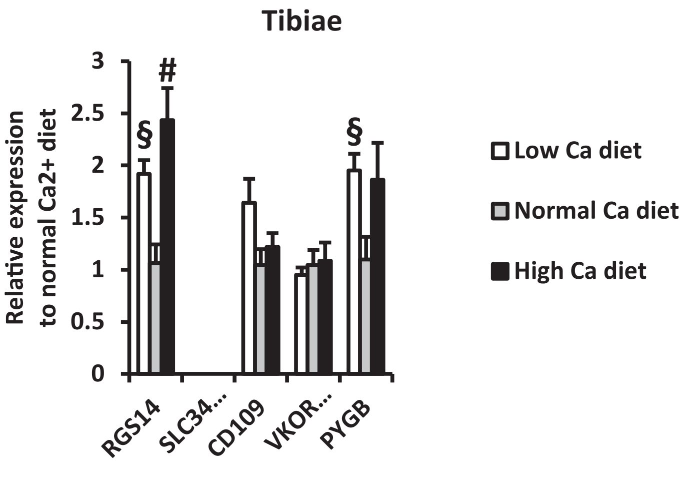

Supplement: Figure S8 — Relative expression of genes in non-replicated loci under various calcium diets. Data are means± SEM of values obtained from 5 mice fed a low (0.17%) and high (1.69%) calcium diet compared to mice fed a normal calcium diet (0.82%). Expression levels were normalized to actin. Statistical difference was calculated using unpaired t-test. *: P value≤0.05 (low compared to high); §: P value≤0.05 (low compared to normal); #: P value≤0.05 (high compared to normal). (PDF) [file pgen.1003796.s008.pdf]
