# Supplementary material for: Meta-Analysis of Genome-Wide Association Studies Identifies Six New Loci for Serum Calcium Concentrations
Source: PLoS Genet. 2013 Sep 19;9(9):e1003796. doi: 10.1371/journal.pgen.1003796 (PMC3778004; doi:10.1371/journal.pgen.1003796)
Supplement: Table S1 — Characteristics of study participants in discovery and replication cohorts. Data are mean (SD) unless otherwise specified for each discovery and replication studies. (DOCX) [file pgen.1003796.s009.docx]

## Table S1: Characteristics of study participants in discovery and replication cohorts

| **Cohort** | **Sample  size** | **Calcium mg/dL, mean (SD)** | **Female,  % (N)** | **Age, years, mean (SD)** | **Albumin g/dL, mean (SD)** |
| --- | --- | --- | --- | --- | --- |
| **Discovery cohorts** |  |  |  |  |  |
| AGES | 1949 | 9.42 (0.48) | 56.6 (1103) | 77.0 (5.4) | NA |
| ARIC | 9049 | 9.77 (0.41) | 52.9 (4790) | 54.3 (5.7) | NA |
| BLSA | 719 | 9.28 (0.45) | 54.5 (392) | 71.1 (15.0) | 4.1 (0.4) |
| CHS | 1802 | 9.45 (0.35) | 69.4 (1228) | 71.2 (4.6) | NA |
| CoLaus | 5411 | 9.16 (0.38) | 53.1 (2871) | 53.4 (10.7) | 4.4 (0.3) |
| CROATIA-Korcula | 880 | 9.26 (0.54) | 65.0 (572) | 56.2 (13.9) | 4.3 (0.3) |
| CROATIA-Split | 488 | 9.79 (0.64) | 58.6 (286) | 49.1 (14.6) | 4.5 (0.4) |
| CROATIA-Vis | 910 | 9.34 (0.64) | 57.7 (525) | 56.4 (15.5) | 4.5 (0.4) |
| FHS | 2853 | 9.61 (0.37) | 52.1 (1491) | 43.6 (9.8) | 4.5 (0.3) |
| HABC | 1554 | 8.82 (0.42) | 46.8 (728) | 74.8 (2.8) | NA |
| InCHIANTI | 1204 | 9.44 (0.47) | 55.6 (669) | 68.2 (15.5) | 4.2 (0.3) |
| LBC1936 | 993 | 9.40 (0.39) | 49.6 (493) | 69.6 (0.8) | 4.5 (0.3) |
| LOLIPOP EW A | 589 | 9.27 (0.36) | 12.9 (76) | 54.3 (10.4) | 4.4 (0.3) |
| LOLIPOP EW P | 652 | 9.32 (0.38) | 0 | 55.7 (9.1) | 4.4 (0.3) |
| LOLIPOP EW610 | 927 | 9.12 (0.33) | 26.9 (249) | 56.0 (9.8) | 4.4 (0.3) |
| OGP Talana | 1039 | 9.22 (0.50) | 55.9 (581) | 43.8 (22.0) | 4.3 (0.3) |
| ORCADES | 877 | 9.24 (0.36) | 55.3 (485) | 53.5 (15.7) | 4.2 (0.3) |
| RS | 3436 | 9.54 (0.63) | 59.4 (3547) | 69.4 (9.1) | NA |
| SHIP | 4068 | 9.66 (0.46) | 50.7 (2064) | 49.7 (16.3) | NA |
| **TOTAL** | **39,400** |  |  |  |  |
| **Replication cohorts** |  |  |  |  |  |
| BRIGHT | 1855 | 9.06 (1.37) | 57.5 (1127) | 59.6 (12.1) | 4.4 (2.8) |
| Bus Santé | 4670 | 9.33 (0.50) | 49.3 (2'304) | 57.7 (11.4) | NA |
| INGI-Carlantino | 499 | 9.35 (0.50) | 61.1 (305) | 45.0 (20.3) | NA |
| INGI-FVG | 1432 | 9.91 (0.70) | 58.4 (837) | 48.2 (19.7) | NA |
| INGI-CILENTO | 1147 | 9.79 (0.55) | 55.0 (631) | 51.3 (19.3) | 4.0 (0.5) |
| KORA F3 | 1640 | 9.48 (0.45) | 50.5 (829) | 62.3 (10.1) | NA |
| KORA F4 | 1809 | 9.60 (0.46) | 51.2 (927) | 60.9 (8.9) | NA |
| LURIC | 2927 | 9.31 (0.43) | 30.2 (885) | 62.6 (10.7) | 4.4 (0.6) |
| PIVUS | 945 | 9.44 (0.51) | 50.1 (474) | 70.2 (0.2) | 4.0 (0.3) |
| SHIP-Trend | 986 | 9.20 (0.40) | 56.2 (554) | 50.1 (13.7) | NA |
| TwinsUK | 3965 | 9.44 (0.43) | 93.9 (3'724) | 48.1 (12.9) | NA |
| **TOTAL** | **21,875** |  |  |  |  |
