# Supplementary material for: Meta-Analysis of Genome-Wide Association Studies Identifies Six New Loci for Serum Calcium Concentrations
Source: PLoS Genet. 2013 Sep 19;9(9):e1003796. doi: 10.1371/journal.pgen.1003796 (PMC3778004; doi:10.1371/journal.pgen.1003796)
Supplement: Table S2 — SNPs brought forward for replication that did not replicate. Chr, chromosome. A1, effect allele. A2, non-effect allele. Effect A1, regression coefficient for the A1 allele. SE, standard error. Freq A1,frequency of allele A1. (DOCX) [file pgen.1003796.s010.docx]

## Table S2: SNPs brought forward for replication that did not replicate

|  |  |  |  |  |  |  | **Discovery analysis** | | | | **Replication analysis** | | | | **Meta-analysis (discovery + replication)** | | | |
| --- | --- | --- | --- | --- | --- | --- | --- | --- | --- | --- | --- | --- | --- | --- | --- | --- | --- | --- |
| **Markers*** | **Chr** | **Position** | **Nearby Gene** | **A1** | **A2** | **Freq A1** | **N** | **Effect A1** | **SE** | **P value** | **N** | **Effect A1** | **SE** | **P value (1-sided)** | **N** | **Effect A1** | **SE** | **P value** |
| rs2885836 | 3 | 25170127 | *RARBTOP2B* | a | g | 0.24 | 39400 | 0.018 | 0.004 | 6.9E-07 | 21570 | -0.002 | 0.006 | 1.00 | 60970 | 0.012 | 0.003 | 5.4E-05 |
| rs4074995 | 5 | 176729949 | *RGS14, SLC34A1* | a | g | 0.28 | 39400 | 0.017 | 0.003 | 2.4E-07 | 21587 | 0.003 | 0.005 | 0.30 | 60987 | 0.013 | 0.003 | 4.6E-06 |
| rs9447004 | 6 | 74515458 | *CD109* | a | g | 0.48 | 39400 | 0.014 | 0.003 | 8.1E-06 | 21658 | 0.008 | 0.005 | 0.04 | 61058 | 0.012 | 0.003 | 3.3E-06 |
| rs11967485 | 6 | 157169949 | *ARID1B* | a | g | 0.10 | 38361 | -0.033 | 0.006 | 2.5E-07 | 21660 | -0.010 | 0.010 | 0.14 | 60021 | -0.026 | 0.005 | 9.4E-07 |
| rs17711722 | 7 | 64908632 | *VKORC1L1* | t | c | 0.47 | 39400 | 0.021 | 0.003 | 2.8E-11 | 21573 | 0.0003 | 0.005 | 0.48 | 60973 | 0.015 | 0.003 | 8.2E-09 |
| rs12150338 | 17 | 1580854 | *WDR81;SERPINF2* | t | c | 0.09 | 38361 | 0.037 | 0.007 | 6.5E-07 | 21649 | 0.013 | 0.011 | 0.12 | 60010 | 0.030 | 0.006 | 1.5E-06 |
| rs2281558 | 20 | 25188189 | *PYGB* | t | g | 0.25 | 32748 | 0.020 | 0.004 | 6.4E-07 | 21578 | 0.0045 | 0.0056 | 0.21 | 54326 | 0.015 | 0.003 | 5.1E-06 |
