# Supplementary material for: Meta-Analysis of Genome-Wide Association Studies Identifies Six New Loci for Serum Calcium Concentrations
Source: PLoS Genet. 2013 Sep 19;9(9):e1003796. doi: 10.1371/journal.pgen.1003796 (PMC3778004; doi:10.1371/journal.pgen.1003796)
Supplement: Table S3 — SNPs with P value<5*E-05 for uncorrected calcium in Europeans (discovery). Chr, chromosome. Position, position on build 36. A1, allele 1 (effect allele). A2, allele 2. Freq A1, frequency of allele 1. InRefGen, gene symbol if SNP is located within a specific gene. (DOCX) [file pgen.1003796.s011.docx]

## Table S3: SNPs with P value <5*E-05 for uncorrected calcium in Europeans (discovery)

| **SNP** | **chr** | **position** | **Effect A1** | **SE** | **P value** | **A1** | **A2** | **Freq A1** | **InRefGene** |
| --- | --- | --- | --- | --- | --- | --- | --- | --- | --- |
| rs1801725 | 3 | 123486447 | 0.0689 | 0.0043 | 6.52E-59 | t | g | 0.1523 | CASR |
| rs17251221 | 3 | 123475937 | -0.0702 | 0.0043 | 7.89E-59 | a | g | 0.8484 | CASR |
| rs17265703 | 3 | 123531334 | -0.0602 | 0.0041 | 2.25E-48 | a | g | 0.8353 | CSTA |
| rs5008830 | 3 | 123513152 | 0.06 | 0.0041 | 1.58E-47 | a | g | 0.1645 |  |
| rs2001548 | 3 | 123515479 | 0.0591 | 0.0041 | 1.37E-46 | a | g | 0.1649 |  |
| rs16832956 | 3 | 123500198 | -0.0491 | 0.0038 | 4.97E-39 | c | g | 0.7946 |  |
| rs16833080 | 3 | 123583597 | 0.0516 | 0.0042 | 2.56E-34 | t | c | 0.1534 | CCDC58 |
| rs16833078 | 3 | 123582284 | -0.0514 | 0.0042 | 2.82E-34 | a | g | 0.8462 | CCDC58 |
| rs9834317 | 3 | 123572049 | 0.0512 | 0.0042 | 3.92E-34 | t | g | 0.1541 | CCDC58 |
| rs6438725 | 3 | 123570796 | -0.0511 | 0.0042 | 4.56E-34 | t | c | 0.8458 | CCDC58 |
| rs4491840 | 3 | 123563437 | 0.0507 | 0.0042 | 5.27E-34 | a | g | 0.1549 | CCDC58 |
| rs6791616 | 3 | 123589925 | 0.0485 | 0.0041 | 6.43E-33 | t | c | 0.1671 | C3orf28 |
| rs12107092 | 3 | 123606538 | 0.0487 | 0.0041 | 7.28E-33 | t | c | 0.1662 | C3orf28 |
| rs10222633 | 3 | 123459616 | -0.0343 | 0.003 | 1.13E-29 | a | g | 0.4856 | CASR |
| rs17200894 | 3 | 123612831 | -0.047 | 0.0042 | 2.49E-29 | c | g | 0.8409 |  |
| rs17201246 | 3 | 123628160 | 0.0466 | 0.0042 | 6.47E-29 | a | c | 0.1593 | KPNA1 |
| rs3749203 | 3 | 123459184 | -0.0349 | 0.0031 | 1.39E-28 | t | c | 0.486 | CASR |
| rs9789994 | 3 | 123700541 | 0.0463 | 0.0042 | 1.51E-28 | a | t | 0.1589 | KPNA1 |
| rs3792289 | 3 | 123476290 | -0.0356 | 0.0032 | 4.20E-28 | a | g | 0.6656 | CASR |
| rs13095172 | 3 | 123472947 | 0.0356 | 0.0032 | 4.22E-28 | t | c | 0.3344 | CASR |
| rs16833168 | 3 | 123737337 | 0.0461 | 0.0042 | 4.69E-28 | t | c | 0.1578 | PARP9 |
| rs17267388 | 3 | 123750236 | 0.045 | 0.0041 | 4.71E-28 | a | g | 0.1682 | PARP9 |
| rs2270859 | 3 | 123738398 | 0.046 | 0.0042 | 6.94E-28 | a | g | 0.1578 | PARP9 |
| rs10934578 | 3 | 123459972 | 0.0352 | 0.0032 | 8.35E-28 | t | g | 0.3343 | CASR |
| rs3749208 | 3 | 123462974 | 0.0351 | 0.0032 | 1.18E-27 | t | c | 0.3343 | CASR |
| rs11929034 | 3 | 123761739 | 0.0468 | 0.0043 | 1.46E-27 | a | g | 0.1599 | PARP9 |
| rs13085498 | 3 | 123494053 | 0.0353 | 0.0032 | 1.88E-27 | t | c | 0.3357 |  |
| rs13085674 | 3 | 123494041 | 0.0353 | 0.0032 | 1.93E-27 | a | g | 0.3357 |  |
| rs10934582 | 3 | 123496044 | 0.0344 | 0.0033 | 5.33E-26 | a | g | 0.3378 |  |
| rs4306808 | 3 | 123611156 | -0.0594 | 0.0057 | 1.69E-25 | c | g | 0.8963 | C3orf28 |
| rs17266816 | 3 | 123611564 | 0.0586 | 0.0057 | 8.73E-25 | a | g | 0.1036 | C3orf28 |
| rs12635478 | 3 | 123491243 | 0.0337 | 0.0033 | 6.47E-24 | a | c | 0.3243 |  |
| rs13083990 | 3 | 123497256 | -0.0323 | 0.0032 | 1.64E-23 | t | c | 0.647 |  |
| rs6438720 | 3 | 123499922 | 0.0304 | 0.0032 | 1.77E-21 | a | c | 0.4101 |  |
| rs1067 | 3 | 123615655 | 0.0381 | 0.004 | 1.87E-21 | a | g | 0.1995 | WDR5B |
| rs6768471 | 3 | 123462394 | -0.0298 | 0.0032 | 5.28E-21 | a | g | 0.3551 | CASR |
| rs11716910 | 3 | 123470309 | 0.0297 | 0.0032 | 6.20E-21 | a | g | 0.6456 | CASR |
| rs11711910 | 3 | 123468572 | 0.0297 | 0.0032 | 6.38E-21 | t | g | 0.6455 | CASR |
| rs7644390 | 3 | 123472918 | -0.0297 | 0.0032 | 7.11E-21 | a | t | 0.3551 | CASR |
| rs11922857 | 3 | 123480078 | -0.0297 | 0.0032 | 7.61E-21 | a | g | 0.3548 | CASR |
| rs4678173 | 3 | 123473829 | -0.0296 | 0.0032 | 8.15E-21 | a | c | 0.3551 | CASR |
| rs9864290 | 3 | 123522752 | -0.0308 | 0.0033 | 2.64E-20 | t | c | 0.7082 |  |
| rs3804590 | 3 | 123460763 | 0.0338 | 0.0037 | 2.85E-20 | t | g | 0.3308 | CASR |
| rs4678180 | 3 | 123520487 | 0.0307 | 0.0033 | 3.21E-20 | t | c | 0.2919 |  |
| rs6803098 | 3 | 123523300 | -0.0307 | 0.0033 | 3.62E-20 | t | c | 0.7082 |  |
| rs7646147 | 3 | 123489217 | 0.0288 | 0.0032 | 8.97E-20 | t | c | 0.6496 |  |
| rs9740 | 3 | 123487743 | 0.0288 | 0.0032 | 9.77E-20 | a | g | 0.6494 | CASR |
| rs10934581 | 3 | 123493844 | -0.0288 | 0.0032 | 1.14E-19 | t | c | 0.3502 |  |
| rs7633800 | 3 | 123494355 | -0.0287 | 0.0032 | 1.97E-19 | a | g | 0.3499 |  |
| rs7633667 | 3 | 123494416 | 0.0287 | 0.0032 | 1.99E-19 | c | g | 0.6501 |  |
| rs9851884 | 3 | 123502884 | 0.0299 | 0.0033 | 3.14E-19 | a | g | 0.2845 |  |
| rs1472621 | 3 | 123495416 | -0.0332 | 0.0037 | 4.51E-19 | a | g | 0.6679 |  |
| rs11720638 | 3 | 123495657 | -0.0279 | 0.0032 | 3.80E-18 | c | g | 0.3499 |  |
| rs4678192 | 3 | 123610282 | -0.0275 | 0.0032 | 5.45E-18 | a | g | 0.6116 | C3orf28 |
| rs9856248 | 3 | 123546501 | 0.0293 | 0.0034 | 1.34E-17 | t | c | 0.2713 |  |
| rs1402200 | 3 | 123505107 | -0.0282 | 0.0033 | 1.63E-17 | c | g | 0.6958 |  |
| rs11926904 | 3 | 123428845 | 0.0654 | 0.0077 | 1.98E-17 | t | g | 0.0462 | CASR |
| rs12185943 | 3 | 123498259 | 0.0265 | 0.0032 | 2.01E-16 | a | g | 0.6579 |  |
| rs2001547 | 3 | 123515524 | -0.025 | 0.0032 | 7.62E-15 | a | g | 0.3343 |  |
| rs17252533 | 3 | 123504897 | -0.025 | 0.0032 | 8.57E-15 | t | g | 0.3355 |  |
| rs1127343 | 3 | 123611084 | -0.025 | 0.0032 | 9.42E-15 | a | g | 0.6062 | C3orf28 |
| rs16833133 | 3 | 123627563 | 0.0553 | 0.0074 | 9.35E-14 | a | g | 0.0625 | KPNA1 |
| rs7618100 | 3 | 123432810 | -0.0252 | 0.0034 | 1.24E-13 | c | g | 0.7115 | CASR |
| rs9811123 | 3 | 123431982 | -0.0251 | 0.0034 | 1.60E-13 | a | g | 0.7113 | CASR |
| rs16833165 | 3 | 123730363 | -0.0549 | 0.0074 | 1.63E-13 | t | c | 0.9376 | PARP9 |
| rs9866419 | 3 | 123422899 | -0.0251 | 0.0034 | 1.78E-13 | a | g | 0.7113 | CASR |
| rs6798997 | 3 | 123557394 | -0.0211 | 0.0031 | 6.06E-12 | a | g | 0.4108 |  |
| rs6768280 | 3 | 123607742 | -0.0212 | 0.0031 | 6.49E-12 | a | g | 0.4102 | C3orf28 |
| rs9875101 | 3 | 123450780 | 0.0229 | 0.0033 | 6.81E-12 | t | c | 0.301 | CASR |
| rs1979869 | 3 | 123445975 | 0.0228 | 0.0033 | 7.03E-12 | t | c | 0.3018 | CASR |
| rs12493480 | 3 | 123445653 | -0.0228 | 0.0033 | 7.09E-12 | a | g | 0.6982 | CASR |
| rs7635354 | 3 | 123449328 | -0.0228 | 0.0033 | 8.06E-12 | a | c | 0.6987 | CASR |
| rs2173961 | 3 | 123451537 | 0.0229 | 0.0033 | 8.07E-12 | t | g | 0.3006 | CASR |
| rs2134221 | 3 | 123444936 | -0.0227 | 0.0033 | 8.10E-12 | a | g | 0.6979 | CASR |
| rs13326577 | 3 | 123447936 | 0.0227 | 0.0033 | 8.21E-12 | a | t | 0.3015 | CASR |
| rs3863977 | 3 | 123446952 | 0.0227 | 0.0033 | 8.24E-12 | t | c | 0.3015 | CASR |
| rs1965358 | 3 | 123452991 | -0.0229 | 0.0034 | 8.56E-12 | a | g | 0.6989 | CASR |
| rs1463892 | 3 | 123453076 | 0.0229 | 0.0034 | 9.83E-12 | a | g | 0.3012 | CASR |
| rs937626 | 3 | 123454492 | -0.0235 | 0.0035 | 1.20E-11 | a | g | 0.6949 | CASR |
| rs12487598 | 3 | 123661292 | 0.0208 | 0.0031 | 1.88E-11 | t | c | 0.5905 | KPNA1 |
| rs9818363 | 3 | 123594981 | 0.0204 | 0.0031 | 2.76E-11 | t | c | 0.5724 | C3orf28 |
| rs17711722 | 7 | 64908632 | 0.0208 | 0.0031 | 2.78E-11 | t | c | 0.4734 |  |
| rs780094 | 2 | 27594741 | 0.0204 | 0.0031 | 3.69E-11 | t | c | 0.4136 | GCKR |
| rs780093 | 2 | 27596107 | 0.0204 | 0.0031 | 4.07E-11 | t | c | 0.4132 | GCKR |
| rs7791814 | 7 | 65002924 | -0.0201 | 0.003 | 4.13E-11 | t | c | 0.5557 | VKORC1L1 |
| rs11763147 | 7 | 64964256 | 0.0201 | 0.003 | 4.18E-11 | a | g | 0.4443 |  |
| rs4718286 | 7 | 64930199 | 0.0202 | 0.0031 | 4.28E-11 | a | g | 0.4453 |  |
| rs4502988 | 7 | 64935181 | 0.0202 | 0.0031 | 4.29E-11 | a | g | 0.4453 |  |
| rs6438737 | 3 | 123711664 | -0.0203 | 0.0031 | 4.45E-11 | t | c | 0.4117 | KPNA1 |
| rs13320117 | 3 | 123610512 | -0.0242 | 0.0037 | 5.08E-11 | t | c | 0.6407 | C3orf28 |
| rs4678191 | 3 | 123603928 | 0.0206 | 0.0032 | 6.49E-11 | t | g | 0.528 | C3orf28 |
| rs7784623 | 7 | 65032469 | 0.0198 | 0.003 | 7.54E-11 | t | g | 0.444 | VKORC1L1 |
| rs6960048 | 7 | 65045474 | 0.0198 | 0.003 | 7.77E-11 | a | g | 0.444 | VKORC1L1 |
| rs10807697 | 7 | 65053605 | 0.0198 | 0.003 | 8.68E-11 | a | g | 0.444 | VKORC1L1 |
| rs1260326 | 2 | 27584444 | 0.0201 | 0.0031 | 8.89E-11 | t | c | 0.4242 | GCKR |
| rs1829943 | 7 | 64913843 | 0.02 | 0.0031 | 8.90E-11 | a | g | 0.4454 |  |
| rs1917563 | 7 | 65053082 | 0.0197 | 0.003 | 9.05E-11 | a | g | 0.4441 | VKORC1L1 |
| rs9530 | 7 | 65063329 | 0.0197 | 0.003 | 9.26E-11 | a | g | 0.4438 | GUSB |
| rs781143 | 7 | 65077314 | -0.0198 | 0.0031 | 9.69E-11 | c | g | 0.5558 | GUSB |
| rs1631091 | 7 | 65103375 | 0.0198 | 0.0031 | 9.97E-11 | t | g | 0.4443 |  |
| rs2658585 | 7 | 65099376 | 0.0197 | 0.0031 | 1.01E-10 | a | t | 0.4443 |  |
| rs1701760 | 7 | 65111123 | 0.0197 | 0.0031 | 1.02E-10 | a | g | 0.4443 |  |
| rs1723268 | 7 | 65110515 | 0.0197 | 0.0031 | 1.03E-10 | a | c | 0.4443 |  |
| rs6947132 | 7 | 64910930 | 0.0199 | 0.0031 | 1.12E-10 | a | g | 0.4458 |  |
| rs2690442 | 7 | 64873311 | 0.0196 | 0.0031 | 2.36E-10 | t | c | 0.4461 |  |
| rs4718278 | 7 | 64881031 | 0.0196 | 0.0031 | 2.55E-10 | t | c | 0.446 |  |
| rs11768292 | 7 | 65112236 | 0.0199 | 0.0032 | 3.01E-10 | t | g | 0.4052 |  |
| rs6808422 | 3 | 123499876 | -0.0195 | 0.0031 | 5.27E-10 | t | c | 0.4874 |  |
| rs7481584 | 11 | 2985665 | -0.0208 | 0.0034 | 9.21E-10 | a | g | 0.2937 | CARS |
| rs729662 | 11 | 2984716 | -0.0208 | 0.0034 | 9.30E-10 | a | g | 0.2938 | CARS |
| rs2583435 | 11 | 2915394 | 0.0201 | 0.0034 | 2.19E-09 | t | c | 0.6989 |  |
| rs12805915 | 11 | 2926347 | -0.02 | 0.0033 | 2.44E-09 | a | g | 0.3007 | NAP1L4 |
| rs3814964 | 11 | 2956083 | -0.0199 | 0.0033 | 2.48E-09 | t | g | 0.3011 | NAP1L4 |
| rs4758502 | 11 | 2955037 | -0.0199 | 0.0033 | 2.58E-09 | t | c | 0.3012 | NAP1L4 |
| rs4758621 | 11 | 2966216 | 0.0196 | 0.0033 | 2.66E-09 | a | g | 0.6841 | NAP1L4 |
| rs1468102 | 11 | 2961102 | 0.0196 | 0.0033 | 3.09E-09 | c | g | 0.6838 | NAP1L4 |
| rs12806061 | 11 | 2964980 | -0.0196 | 0.0033 | 3.16E-09 | a | g | 0.3161 | NAP1L4 |
| rs10511410 | 3 | 123611209 | -0.02 | 0.0034 | 4.24E-09 | c | g | 0.2957 | C3orf28 |
| rs2071110 | 11 | 2947795 | 0.0196 | 0.0033 | 4.47E-09 | c | g | 0.6931 | NAP1L4 |
| rs16833051 | 3 | 123550948 | -0.0201 | 0.0034 | 4.96E-09 | a | g | 0.2741 |  |
| rs11024759 | 11 | 2938668 | 0.0195 | 0.0033 | 5.64E-09 | t | c | 0.6932 | NAP1L4 |
| rs3847646 | 11 | 2910299 | -0.025 | 0.0043 | 5.82E-09 | t | c | 0.223 |  |
| rs7628094 | 3 | 123538185 | -0.0176 | 0.003 | 6.00E-09 | a | t | 0.5733 | CSTA |
| rs2911711 | 2 | 27604050 | -0.0179 | 0.0031 | 6.03E-09 | a | t | 0.5338 |  |
| rs1260333 | 2 | 27602128 | 0.0179 | 0.0031 | 6.08E-09 | a | g | 0.4663 |  |
| rs4315641 | 3 | 123551565 | -0.02 | 0.0034 | 6.09E-09 | a | t | 0.2761 |  |
| rs2001006 | 11 | 2931488 | 0.0195 | 0.0033 | 6.12E-09 | t | c | 0.6937 | NAP1L4 |
| rs6438722 | 3 | 123536405 | -0.0176 | 0.003 | 6.51E-09 | a | g | 0.5734 | CSTA |
| rs17589 | 3 | 123539122 | -0.0176 | 0.003 | 7.03E-09 | t | c | 0.5734 | CSTA |
| rs6968619 | 7 | 65706302 | -0.0171 | 0.003 | 1.56E-08 | t | c | 0.4945 |  |
| rs756693 | 11 | 2984465 | 0.0202 | 0.0036 | 1.65E-08 | a | g | 0.7238 | CARS |
| rs11920570 | 3 | 123572792 | -0.0193 | 0.0034 | 1.72E-08 | a | g | 0.2741 | CCDC58 |
| rs10258739 | 7 | 65700370 | -0.017 | 0.003 | 1.76E-08 | a | t | 0.495 |  |
| rs12633068 | 3 | 123578264 | 0.0193 | 0.0034 | 1.84E-08 | c | g | 0.7259 | CCDC58 |
| rs801191 | 7 | 65670390 | 0.017 | 0.003 | 2.18E-08 | a | g | 0.5064 |  |
| rs587360 | 7 | 65160133 | -0.0172 | 0.0031 | 2.65E-08 | a | c | 0.4382 |  |
| rs429289 | 11 | 3029152 | -0.0222 | 0.004 | 3.14E-08 | t | c | 0.259 | CARS |
| rs1570669 | 20 | 52207834 | -0.0178 | 0.0032 | 3.98E-08 | a | g | 0.6603 | CYP24A1 |
| rs1550532 | 2 | 233929587 | 0.0179 | 0.0033 | 4.60E-08 | c | g | 0.312 | DGKD |
| rs4718287 | 7 | 64944462 | -0.017 | 0.0031 | 5.39E-08 | c | g | 0.3948 |  |
| rs11767457 | 7 | 64928050 | -0.017 | 0.0031 | 5.43E-08 | t | c | 0.3954 |  |
| rs7798244 | 7 | 64924890 | -0.017 | 0.0031 | 5.50E-08 | a | g | 0.3954 |  |
| rs6950899 | 7 | 64924084 | -0.017 | 0.0031 | 5.71E-08 | a | g | 0.3956 |  |
| rs7778644 | 7 | 64965891 | 0.0169 | 0.0031 | 5.73E-08 | t | c | 0.6038 |  |
| rs10950022 | 7 | 65013891 | -0.0167 | 0.0031 | 7.80E-08 | t | c | 0.3957 | VKORC1L1 |
| rs7786356 | 7 | 65042610 | 0.0166 | 0.0031 | 8.70E-08 | c | g | 0.6038 | VKORC1L1 |
| rs12538430 | 7 | 65057623 | 0.0166 | 0.0031 | 9.37E-08 | t | c | 0.6041 |  |
| rs4535605 | 7 | 65052860 | 0.0166 | 0.0031 | 9.46E-08 | c | g | 0.6038 | VKORC1L1 |
| rs6438726 | 3 | 123593611 | -0.0182 | 0.0034 | 9.71E-08 | t | c | 0.2701 | C3orf28 |
| rs6460293 | 7 | 65447627 | 0.0166 | 0.0031 | 1.09E-07 | a | c | 0.6187 | TPST1 |
| rs4499980 | 7 | 64854059 | 0.0166 | 0.0031 | 1.13E-07 | a | g | 0.6041 |  |
| rs1568780 | 7 | 64841022 | -0.0166 | 0.0031 | 1.14E-07 | a | g | 0.3958 |  |
| rs4718296 | 7 | 65073571 | 0.0164 | 0.0031 | 1.20E-07 | a | c | 0.6039 | GUSB |
| rs11760664 | 7 | 64831205 | 0.0166 | 0.0031 | 1.21E-07 | a | g | 0.6044 |  |
| rs7635930 | 3 | 123595247 | -0.018 | 0.0034 | 1.23E-07 | c | g | 0.2699 | C3orf28 |
| rs12540589 | 7 | 65075209 | -0.0164 | 0.0031 | 1.34E-07 | t | c | 0.3961 | GUSB |
| rs12535204 | 7 | 65075255 | -0.0164 | 0.0031 | 1.36E-07 | a | g | 0.396 | GUSB |
| rs1167406 | 7 | 65194371 | 0.0164 | 0.0031 | 1.39E-07 | c | g | 0.621 | ASL |
| rs778736 | 7 | 65451283 | -0.0165 | 0.0031 | 1.43E-07 | t | c | 0.3815 | TPST1 |
| rs6959268 | 7 | 65450401 | 0.0165 | 0.0031 | 1.44E-07 | a | g | 0.6185 | TPST1 |
| rs7783779 | 7 | 65434061 | -0.0165 | 0.0031 | 1.51E-07 | a | g | 0.38 | TPST1 |
| rs709609 | 7 | 65197996 | -0.0163 | 0.0031 | 1.55E-07 | a | g | 0.3795 |  |
| rs1167390 | 7 | 65213328 | 0.0163 | 0.0031 | 1.55E-07 | a | g | 0.6201 |  |
| rs10447522 | 7 | 65433509 | -0.0164 | 0.0031 | 1.58E-07 | a | g | 0.3802 | TPST1 |
| rs7336933 | 13 | 41457076 | -0.0226 | 0.0043 | 1.61E-07 | a | g | 0.1472 |  |
| rs10228885 | 7 | 65417964 | -0.0164 | 0.0031 | 1.62E-07 | c | g | 0.3799 | TPST1 |
| rs7319447 | 13 | 41449508 | 0.0225 | 0.0043 | 1.71E-07 | a | g | 0.8527 |  |
| rs7328352 | 13 | 41446979 | 0.0225 | 0.0043 | 1.73E-07 | a | g | 0.8522 |  |
| rs1983372 | 7 | 65248786 | 0.0162 | 0.0031 | 1.73E-07 | t | c | 0.619 | RCP9 |
| rs12372841 | 13 | 41447812 | -0.0225 | 0.0043 | 1.74E-07 | c | g | 0.1478 |  |
| rs7333778 | 13 | 41444074 | -0.0223 | 0.0043 | 1.75E-07 | t | g | 0.1482 |  |
| rs1000464 | 7 | 65415344 | 0.0163 | 0.0031 | 1.87E-07 | c | g | 0.6194 | TPST1 |
| rs709596 | 7 | 65463348 | 0.0162 | 0.0031 | 1.87E-07 | a | g | 0.619 |  |
| rs11984115 | 7 | 65411294 | 0.0163 | 0.0031 | 1.88E-07 | t | c | 0.6194 | TPST1 |
| rs2249458 | 7 | 65202509 | 0.0163 | 0.0031 | 1.89E-07 | t | c | 0.6172 |  |
| rs778724 | 7 | 65466726 | 0.0162 | 0.0031 | 1.91E-07 | t | c | 0.6183 |  |
| rs7782704 | 7 | 65442801 | 0.0163 | 0.0031 | 1.93E-07 | a | g | 0.6199 | TPST1 |
| rs7326697 | 13 | 41434401 | 0.0223 | 0.0043 | 1.93E-07 | a | t | 0.8512 |  |
| rs4718298 | 7 | 65112998 | 0.0163 | 0.0031 | 1.93E-07 | c | g | 0.6041 |  |
| rs1643394 | 7 | 65473529 | 0.0162 | 0.0031 | 1.96E-07 | a | g | 0.6181 |  |
| rs1643393 | 7 | 65473530 | -0.0162 | 0.0031 | 2.00E-07 | a | t | 0.3825 |  |
| rs778720 | 7 | 65483710 | -0.0161 | 0.0031 | 2.02E-07 | a | c | 0.3827 | LOC285908 |
| rs4665978 | 2 | 27502230 | 0.0162 | 0.0031 | 2.06E-07 | t | c | 0.5677 |  |
| rs401176 | 7 | 65127077 | -0.0162 | 0.0031 | 2.08E-07 | t | c | 0.396 |  |
| rs2178198 | 2 | 27748577 | 0.0237 | 0.0046 | 2.13E-07 | t | c | 0.1376 | SLC4A1AP |
| rs778711 | 7 | 65489092 | -0.0161 | 0.0031 | 2.13E-07 | a | g | 0.3829 | LOC285908 |
| rs778702 | 7 | 65502270 | -0.0161 | 0.0031 | 2.17E-07 | a | g | 0.3828 | LOC285908 |
| rs908915 | 7 | 65252086 | 0.0161 | 0.0031 | 2.17E-07 | t | g | 0.6189 | RCP9 |
| rs778680 | 7 | 65477849 | -0.0161 | 0.0031 | 2.18E-07 | a | g | 0.3829 |  |
| rs1643375 | 7 | 65510112 | 0.0161 | 0.0031 | 2.24E-07 | t | c | 0.6173 |  |
| rs6947339 | 7 | 65525905 | 0.016 | 0.0031 | 2.27E-07 | t | c | 0.618 |  |
| rs6962717 | 7 | 65521170 | -0.016 | 0.0031 | 2.30E-07 | a | g | 0.3823 |  |
| rs7798630 | 7 | 65408914 | 0.0161 | 0.0031 | 2.33E-07 | c | g | 0.6193 | TPST1 |
| rs6976714 | 7 | 65528896 | -0.016 | 0.0031 | 2.34E-07 | a | g | 0.382 |  |
| rs1875057 | 7 | 65369290 | -0.016 | 0.0031 | 2.34E-07 | a | g | 0.3801 | TPST1 |
| rs2877616 | 3 | 123783260 | 0.0166 | 0.0032 | 2.36E-07 | c | g | 0.6629 |  |
| rs11773628 | 7 | 65620066 | 0.016 | 0.0031 | 2.36E-07 | t | g | 0.6184 |  |
| rs4074995 | 5 | 176729949 | 0.0174 | 0.0034 | 2.42E-07 | a | g | 0.2833 | RGS14 |
| rs11967485 | 6 | 157169949 | -0.0325 | 0.0063 | 2.45E-07 | a | g | 0.1005 | ARID1B |
| rs10263935 | 7 | 65733463 | 0.0157 | 0.003 | 2.46E-07 | a | g | 0.4661 | KCTD7 |
| rs6460282 | 7 | 65328681 | -0.016 | 0.0031 | 2.46E-07 | t | c | 0.3808 | TPST1 |
| rs13232191 | 7 | 65624083 | 0.016 | 0.0031 | 2.50E-07 | a | g | 0.6185 |  |
| rs949930 | 7 | 65404257 | -0.0161 | 0.0031 | 2.51E-07 | c | g | 0.3807 | TPST1 |
| rs7586601 | 2 | 27438170 | 0.0161 | 0.0031 | 2.51E-07 | a | g | 0.5676 |  |
| rs10950043 | 7 | 65626045 | 0.016 | 0.0031 | 2.54E-07 | t | c | 0.6187 |  |
| rs6460302 | 7 | 65597692 | -0.016 | 0.0031 | 2.56E-07 | c | g | 0.3817 |  |
| rs6964437 | 7 | 65323879 | -0.016 | 0.0031 | 2.59E-07 | a | g | 0.3808 | TPST1 |
| rs838709 | 2 | 233949483 | -0.017 | 0.0033 | 2.60E-07 | a | c | 0.6824 | DGKD |
| rs6960446 | 7 | 65370694 | -0.016 | 0.0031 | 2.65E-07 | a | g | 0.3801 | TPST1 |
| rs10224872 | 7 | 65397208 | 0.0161 | 0.0031 | 2.68E-07 | a | t | 0.6194 | TPST1 |
| rs13226170 | 7 | 65636733 | -0.0159 | 0.0031 | 2.69E-07 | a | g | 0.3813 |  |
| rs1986617 | 3 | 123599247 | -0.0172 | 0.0033 | 2.70E-07 | a | t | 0.2858 | C3orf28 |
| rs7624614 | 3 | 123786619 | -0.0164 | 0.0032 | 2.72E-07 | a | c | 0.3372 |  |
| rs6952182 | 7 | 65320752 | -0.0159 | 0.0031 | 2.74E-07 | t | g | 0.3812 | TPST1 |
| rs6460304 | 7 | 65602163 | -0.0159 | 0.0031 | 2.76E-07 | a | g | 0.382 |  |
| rs838716 | 2 | 233959530 | -0.0168 | 0.0033 | 2.77E-07 | c | g | 0.6881 | DGKD |
| rs7648255 | 3 | 123650370 | -0.0176 | 0.0034 | 2.77E-07 | a | t | 0.2682 | KPNA1 |
| rs838708 | 2 | 233949138 | -0.0168 | 0.0033 | 2.80E-07 | a | g | 0.6892 | DGKD |
| rs10282433 | 7 | 65358874 | 0.0159 | 0.0031 | 2.80E-07 | a | g | 0.6183 | TPST1 |
| rs6785609 | 3 | 123785505 | 0.0164 | 0.0032 | 2.82E-07 | t | c | 0.6628 |  |
| rs801217 | 7 | 65648012 | 0.0157 | 0.003 | 2.82E-07 | t | c | 0.4661 |  |
| rs12698534 | 7 | 65624280 | -0.0156 | 0.003 | 2.85E-07 | t | c | 0.5333 |  |
| rs9880647 | 3 | 123789610 | 0.0164 | 0.0032 | 2.85E-07 | a | g | 0.6628 |  |
| rs12537823 | 7 | 65358319 | -0.0159 | 0.0031 | 2.89E-07 | a | g | 0.3814 | TPST1 |
| rs2302918 | 7 | 65638367 | 0.0156 | 0.003 | 2.89E-07 | a | g | 0.4664 |  |
| rs4718330 | 7 | 65352678 | 0.0159 | 0.0031 | 3.03E-07 | a | g | 0.6182 | TPST1 |
| rs10261398 | 7 | 65387599 | -0.0159 | 0.0031 | 3.04E-07 | a | g | 0.3812 | TPST1 |
| rs4236208 | 7 | 65386513 | 0.0159 | 0.0031 | 3.06E-07 | t | c | 0.6189 | TPST1 |
| rs4149461 | 7 | 65382167 | -0.0159 | 0.0031 | 3.06E-07 | t | c | 0.3807 | TPST1 |
| rs6950137 | 7 | 65614045 | -0.0158 | 0.0031 | 3.09E-07 | a | g | 0.3819 |  |
| rs1532571 | 7 | 65386934 | -0.0161 | 0.0032 | 3.12E-07 | t | c | 0.374 | TPST1 |
| rs4677951 | 3 | 123641930 | 0.0175 | 0.0034 | 3.13E-07 | c | g | 0.7317 | KPNA1 |
| rs10215132 | 7 | 65691841 | 0.0156 | 0.003 | 3.15E-07 | a | c | 0.4634 |  |
| rs10950044 | 7 | 65680411 | -0.0155 | 0.003 | 3.27E-07 | a | g | 0.5364 |  |
| rs6943696 | 7 | 65685720 | -0.0155 | 0.003 | 3.28E-07 | t | c | 0.5365 |  |
| rs801193 | 7 | 65668047 | -0.0155 | 0.003 | 3.30E-07 | t | g | 0.5348 |  |
| rs6952407 | 7 | 65682947 | -0.0157 | 0.0031 | 3.32E-07 | a | g | 0.5315 |  |
| rs2013222 | 7 | 65673371 | -0.0155 | 0.003 | 3.36E-07 | c | g | 0.5361 |  |
| rs9791713 | 7 | 65742633 | 0.0158 | 0.0031 | 3.38E-07 | a | c | 0.6208 | KCTD7 |
| rs1860469 | 7 | 65744310 | -0.0158 | 0.0031 | 3.39E-07 | a | g | 0.3792 |  |
| rs12673450 | 7 | 65646655 | -0.0158 | 0.0031 | 3.40E-07 | a | g | 0.3799 |  |
| rs801190 | 7 | 65670468 | 0.0155 | 0.003 | 3.41E-07 | t | c | 0.4642 |  |
| rs11760844 | 7 | 65377318 | -0.0158 | 0.0031 | 3.42E-07 | c | g | 0.3802 | TPST1 |
| rs1860472 | 7 | 65720158 | -0.0158 | 0.0031 | 3.46E-07 | t | c | 0.3799 |  |
| rs4075958 | 5 | 176717118 | 0.0172 | 0.0034 | 3.53E-07 | a | g | 0.2762 |  |
| rs4406286 | 7 | 64793111 | -0.0166 | 0.0033 | 3.55E-07 | a | g | 0.3998 |  |
| rs10256544 | 7 | 65312563 | 0.0158 | 0.0031 | 3.58E-07 | a | g | 0.6189 | TPST1 |
| rs6971059 | 7 | 65704467 | -0.0158 | 0.0031 | 3.59E-07 | a | g | 0.38 |  |
| rs6967708 | 7 | 65294748 | -0.0158 | 0.0031 | 3.61E-07 | a | t | 0.3815 |  |
| rs880166 | 7 | 65308197 | -0.0158 | 0.0031 | 3.63E-07 | t | c | 0.3813 | TPST1 |
| rs2420174 | 7 | 65282796 | 0.0158 | 0.0031 | 3.65E-07 | t | c | 0.619 |  |
| rs6460307 | 7 | 65698306 | 0.0158 | 0.0031 | 3.66E-07 | a | g | 0.6198 |  |
| rs4718383 | 7 | 65745844 | -0.0158 | 0.0031 | 3.67E-07 | t | c | 0.3794 |  |
| rs9818228 | 3 | 123333279 | -0.016 | 0.0032 | 3.69E-07 | a | c | 0.5593 |  |
| rs9818482 | 3 | 123333244 | -0.016 | 0.0032 | 3.71E-07 | t | g | 0.5594 |  |
| rs2220626 | 7 | 65183497 | 0.0156 | 0.0031 | 3.73E-07 | t | c | 0.5741 | ASL |
| rs9532963 | 13 | 41472856 | -0.0233 | 0.0046 | 3.78E-07 | a | g | 0.1522 |  |
| rs6460308 | 7 | 65722175 | -0.0155 | 0.003 | 3.83E-07 | t | c | 0.5359 |  |
| rs6951503 | 7 | 65356371 | -0.0159 | 0.0031 | 3.89E-07 | t | g | 0.3867 | TPST1 |
| rs3764903 | 7 | 65735917 | 0.0155 | 0.003 | 3.90E-07 | a | g | 0.4643 | KCTD7 |
| rs10278014 | 7 | 65688699 | 0.0157 | 0.0031 | 4.06E-07 | t | c | 0.6197 |  |
| rs6769837 | 3 | 123680674 | 0.0173 | 0.0034 | 4.09E-07 | a | g | 0.7306 | KPNA1 |
| rs313808 | 7 | 65137308 | 0.0195 | 0.0039 | 4.13E-07 | t | g | 0.6939 |  |
| rs10107 | 7 | 65656376 | 0.0157 | 0.0031 | 4.14E-07 | a | g | 0.6201 |  |
| rs13536 | 7 | 65656625 | 0.0157 | 0.0031 | 4.24E-07 | a | g | 0.6202 |  |
| rs11924963 | 3 | 123604823 | 0.0197 | 0.0039 | 4.30E-07 | a | g | 0.7881 | C3orf28 |
| rs6805271 | 3 | 123695649 | 0.0172 | 0.0034 | 4.48E-07 | t | c | 0.7307 | KPNA1 |
| rs801195 | 7 | 65663550 | -0.0156 | 0.0031 | 4.50E-07 | a | g | 0.38 |  |
| rs10249404 | 7 | 65684159 | 0.0154 | 0.0031 | 4.59E-07 | a | g | 0.4616 |  |
| rs6780306 | 3 | 123708144 | 0.0171 | 0.0034 | 4.89E-07 | t | c | 0.7298 | KPNA1 |
| rs780110 | 2 | 27538892 | -0.0156 | 0.0031 | 4.89E-07 | a | g | 0.4311 | IFT172 |
| rs6955837 | 7 | 65680577 | 0.0156 | 0.0031 | 4.93E-07 | t | c | 0.6198 |  |
| rs17216707 | 20 | 52165769 | 0.0205 | 0.0041 | 6.27E-07 | t | c | 0.7915 |  |
| rs2281558 | 20 | 25188189 | 0.0195 | 0.0039 | 6.40E-07 | t | g | 0.2559 | PYGB |
| rs12150338 | 17 | 1580854 | 0.0369 | 0.0074 | 6.50E-07 | t | c | 0.0967 | WDR81 |
| rs2885836 | 3 | 25170127 | 0.0178 | 0.0036 | 6.87E-07 | a | g | 0.2373 |  |
| rs697968 | 7 | 65172468 | 0.0152 | 0.0031 | 7.11E-07 | a | g | 0.5773 |  |
| rs1183245 | 7 | 65178620 | 0.0152 | 0.0031 | 7.53E-07 | t | c | 0.5774 | ASL |
| rs6780909 | 3 | 123732854 | 0.0168 | 0.0034 | 7.69E-07 | t | g | 0.7294 | PARP9 |
| rs7326009 | 13 | 41400972 | 0.0218 | 0.0044 | 8.16E-07 | a | g | 0.8602 | KIAA0564 |
| rs801211 | 7 | 65653124 | -0.0151 | 0.0031 | 8.56E-07 | t | g | 0.5248 |  |
| rs2191268 | 7 | 65747659 | -0.015 | 0.003 | 8.74E-07 | a | g | 0.5369 |  |
| rs9315872 | 13 | 41388008 | -0.0216 | 0.0044 | 8.74E-07 | t | c | 0.1401 | KIAA0564 |
| rs12104449 | 2 | 27826337 | -0.0242 | 0.0049 | 8.75E-07 | a | g | 0.8692 |  |
| rs6946143 | 7 | 65217157 | 0.015 | 0.0031 | 8.88E-07 | t | g | 0.5767 |  |
| rs4452696 | 7 | 64924147 | -0.0151 | 0.0031 | 8.92E-07 | a | g | 0.4408 |  |
| rs2332240 | 3 | 123779524 | 0.0154 | 0.0031 | 8.93E-07 | a | g | 0.5605 |  |
| rs9532947 | 13 | 41390215 | -0.0217 | 0.0044 | 8.95E-07 | t | c | 0.1396 | KIAA0564 |
| rs2003064 | 13 | 41379712 | 0.0216 | 0.0044 | 9.05E-07 | t | c | 0.8606 | KIAA0564 |
| rs1500533 | 3 | 123780459 | -0.0155 | 0.0032 | 9.06E-07 | a | t | 0.4357 |  |
| rs2036342 | 3 | 123773269 | 0.0221 | 0.0045 | 9.43E-07 | t | c | 0.3012 | DTX3L |
| rs838705 | 2 | 233937981 | -0.0153 | 0.0031 | 9.49E-07 | a | g | 0.6141 | DGKD |
| rs697970 | 7 | 65197487 | 0.015 | 0.0031 | 9.67E-07 | c | g | 0.577 |  |
| rs11986230 | 8 | 134404993 | 0.018 | 0.0037 | 1.05E-06 | t | c | 0.6965 |  |
| rs875971 | 7 | 65255030 | -0.0149 | 0.0031 | 1.07E-06 | t | c | 0.4249 | RCP9 |
| rs7328609 | 13 | 41359189 | 0.0215 | 0.0044 | 1.12E-06 | a | c | 0.86 | KIAA0564 |
| rs838719 | 2 | 233961895 | 0.0152 | 0.0031 | 1.13E-06 | t | c | 0.5949 | DGKD |
| rs970983 | 13 | 41351960 | 0.0215 | 0.0044 | 1.13E-06 | a | c | 0.86 | KIAA0564 |
| rs3812036 | 5 | 176746010 | 0.0179 | 0.0037 | 1.14E-06 | t | c | 0.2562 | SLC34A1 |
| rs9566874 | 13 | 41352094 | 0.0215 | 0.0044 | 1.14E-06 | a | t | 0.86 | KIAA0564 |
| rs7318334 | 13 | 41361027 | -0.0214 | 0.0044 | 1.15E-06 | a | c | 0.1401 | KIAA0564 |
| rs7318484 | 13 | 41361010 | 0.0214 | 0.0044 | 1.16E-06 | t | c | 0.8599 | KIAA0564 |
| rs7332372 | 13 | 41363490 | 0.0214 | 0.0044 | 1.16E-06 | t | c | 0.86 | KIAA0564 |
| rs6547626 | 2 | 27500274 | -0.0153 | 0.0032 | 1.17E-06 | t | c | 0.3789 |  |
| rs8121940 | 20 | 52175713 | 0.0183 | 0.0038 | 1.17E-06 | c | g | 0.7901 |  |
| rs6013897 | 20 | 52175886 | -0.0181 | 0.0037 | 1.19E-06 | a | t | 0.2101 |  |
| rs780090 | 2 | 27571978 | 0.0277 | 0.0057 | 1.20E-06 | t | c | 0.0883 |  |
| rs838713 | 2 | 233954634 | 0.0151 | 0.0031 | 1.22E-06 | c | g | 0.5939 | DGKD |
| rs904593 | 3 | 25168051 | 0.0198 | 0.0041 | 1.24E-06 | t | c | 0.2355 |  |
| rs1728922 | 2 | 27497968 | 0.0153 | 0.0032 | 1.27E-06 | a | c | 0.6211 |  |
| rs4665976 | 2 | 27493829 | 0.0153 | 0.0032 | 1.30E-06 | a | g | 0.6212 |  |
| rs6947487 | 7 | 64718677 | -0.0157 | 0.0032 | 1.32E-06 | a | c | 0.4032 |  |
| rs4147047 | 13 | 41291824 | -0.0215 | 0.0044 | 1.33E-06 | a | g | 0.1397 | KIAA0564 |
| rs17217119 | 20 | 52175997 | 0.018 | 0.0037 | 1.33E-06 | a | g | 0.79 |  |
| rs9532943 | 13 | 41341355 | 0.0214 | 0.0044 | 1.38E-06 | a | c | 0.8599 | KIAA0564 |
| rs1060525 | 2 | 27489086 | 0.0152 | 0.0032 | 1.42E-06 | a | g | 0.6214 |  |
| rs968980 | 13 | 41261839 | 0.0215 | 0.0045 | 1.45E-06 | a | c | 0.86 | KIAA0564 |
| rs2911712 | 2 | 27480449 | 0.0152 | 0.0032 | 1.48E-06 | a | t | 0.6213 | PPM1G |
| rs7339425 | 13 | 41260115 | 0.0215 | 0.0045 | 1.49E-06 | a | g | 0.8599 | KIAA0564 |
| rs7594812 | 2 | 27464973 | 0.0151 | 0.0032 | 1.56E-06 | a | g | 0.621 | PPM1G |
| rs17145704 | 10 | 9368497 | -0.0268 | 0.0056 | 1.57E-06 | t | g | 0.906 |  |
| rs10491003 | 10 | 9368657 | 0.0267 | 0.0056 | 1.59E-06 | t | c | 0.0936 |  |
| rs1647284 | 2 | 27461619 | -0.0151 | 0.0032 | 1.60E-06 | t | c | 0.379 | PPM1G |
| rs12654812 | 5 | 176726797 | 0.0162 | 0.0034 | 1.64E-06 | a | g | 0.299 | RGS14 |
| rs7563162 | 2 | 27484695 | -0.0151 | 0.0032 | 1.65E-06 | t | c | 0.3786 | PPM1G |
| rs1436244 | 3 | 25227645 | 0.0195 | 0.0041 | 1.67E-06 | t | c | 0.1718 |  |
| rs4258924 | 3 | 25174588 | 0.0172 | 0.0036 | 1.68E-06 | a | g | 0.2369 |  |
| rs2010087 | 2 | 27490739 | -0.0151 | 0.0032 | 1.69E-06 | t | c | 0.3746 |  |
| rs1528533 | 2 | 27449260 | -0.0151 | 0.0032 | 1.70E-06 | c | g | 0.3792 | SNX17 |
| rs3739095 | 2 | 27410225 | -0.015 | 0.0031 | 1.73E-06 | a | g | 0.4229 | GTF3C2 |
| rs813592 | 2 | 27575475 | 0.0151 | 0.0032 | 1.83E-06 | t | c | 0.6223 | GCKR |
| rs2280737 | 2 | 27443314 | 0.0151 | 0.0032 | 1.84E-06 | t | c | 0.6254 | EIF2B4 |
| rs9826795 | 3 | 25225870 | 0.0194 | 0.0041 | 1.93E-06 | a | g | 0.172 |  |
| rs6907209 | 6 | 157173357 | -0.0275 | 0.0058 | 1.93E-06 | a | g | 0.0784 | ARID1B |
| rs1260320 | 2 | 27575920 | -0.015 | 0.0032 | 1.97E-06 | a | g | 0.3777 | GCKR |
| rs17145615 | 10 | 9334631 | 0.0331 | 0.0069 | 1.98E-06 | a | t | 0.0645 |  |
| rs17145618 | 10 | 9335226 | -0.033 | 0.0069 | 1.99E-06 | a | g | 0.9359 |  |
| rs12476704 | 2 | 27466535 | -0.015 | 0.0032 | 2.01E-06 | a | c | 0.3746 | PPM1G |
| rs2293571 | 2 | 27582984 | -0.015 | 0.0032 | 2.01E-06 | a | g | 0.3735 | GCKR |
| rs10511409 | 3 | 123610479 | 0.0174 | 0.0037 | 2.05E-06 | t | c | 0.3061 | C3orf28 |
| rs17266628 | 3 | 123610616 | -0.0215 | 0.0045 | 2.06E-06 | a | g | 0.8051 | C3orf28 |
| rs10866705 | 5 | 176733737 | -0.0171 | 0.0036 | 2.08E-06 | a | c | 0.7412 |  |
| rs4425043 | 2 | 27586956 | -0.015 | 0.0032 | 2.11E-06 | a | g | 0.3737 | GCKR |
| rs13472 | 2 | 27453743 | -0.015 | 0.0032 | 2.13E-06 | a | g | 0.3749 | ZNF513 |
| rs6945843 | 7 | 65372218 | 0.0144 | 0.003 | 2.13E-06 | a | g | 0.577 | TPST1 |
| rs1830811 | 10 | 9338392 | -0.0329 | 0.0069 | 2.19E-06 | t | c | 0.9362 |  |
| rs313812 | 7 | 65142478 | -0.0147 | 0.0031 | 2.20E-06 | t | c | 0.4419 |  |
| rs3926380 | 7 | 65718080 | 0.0164 | 0.0035 | 2.21E-06 | t | g | 0.6754 |  |
| rs780104 | 2 | 27531195 | -0.0149 | 0.0032 | 2.23E-06 | a | g | 0.3774 | IFT172 |
| rs780100 | 2 | 27505657 | -0.0149 | 0.0032 | 2.23E-06 | t | g | 0.3774 | NRBP1 |
| rs780106 | 2 | 27535102 | 0.0149 | 0.0032 | 2.24E-06 | a | c | 0.6226 | IFT172 |
| rs7908103 | 10 | 9346070 | -0.0325 | 0.0069 | 2.24E-06 | a | t | 0.9359 |  |
| rs1535867 | 10 | 9345171 | 0.0325 | 0.0069 | 2.24E-06 | t | g | 0.0642 |  |
| rs780102 | 2 | 27512995 | 0.0149 | 0.0032 | 2.25E-06 | t | c | 0.6227 | NRBP1 |
| rs1647266 | 2 | 27546989 | 0.0149 | 0.0032 | 2.25E-06 | t | c | 0.6228 | IFT172 |
| rs780107 | 2 | 27538238 | 0.0149 | 0.0032 | 2.25E-06 | a | g | 0.6226 | IFT172 |
| rs704791 | 2 | 27510671 | 0.0149 | 0.0032 | 2.26E-06 | t | c | 0.6227 | NRBP1 |
| rs780117 | 2 | 27551847 | 0.0149 | 0.0032 | 2.27E-06 | c | g | 0.6226 | IFT172 |
| rs1260341 | 2 | 27516719 | -0.0149 | 0.0032 | 2.27E-06 | a | t | 0.3774 | NRBP1 |
| rs4803 | 2 | 27520801 | 0.0149 | 0.0032 | 2.27E-06 | a | g | 0.6226 | IFT172 |
| rs7096832 | 10 | 9350365 | 0.0324 | 0.0069 | 2.29E-06 | a | g | 0.0639 |  |
| rs1647276 | 2 | 27542105 | -0.0149 | 0.0032 | 2.29E-06 | t | c | 0.3774 | IFT172 |
| rs1260342 | 2 | 27516920 | -0.0149 | 0.0032 | 2.31E-06 | t | g | 0.3774 | NRBP1 |
| rs2173570 | 7 | 65400398 | 0.0145 | 0.0031 | 2.31E-06 | a | t | 0.5761 | TPST1 |
| rs17087784 | 6 | 157171872 | 0.0275 | 0.0058 | 2.32E-06 | a | g | 0.9214 | ARID1B |
| rs2425448 | 20 | 38801835 | 0.0538 | 0.0114 | 2.39E-06 | a | g | 0.0317 |  |
| rs7631296 | 3 | 25222679 | -0.0196 | 0.0042 | 2.40E-06 | c | g | 0.8295 |  |
| rs365896 | 7 | 65148132 | -0.0146 | 0.0031 | 2.42E-06 | a | g | 0.442 |  |
| rs7602534 | 2 | 27445927 | -0.0149 | 0.0032 | 2.43E-06 | t | c | 0.3747 | EIF2B4 |
| rs10267430 | 7 | 65380458 | 0.0144 | 0.0031 | 2.43E-06 | t | g | 0.5762 | TPST1 |
| rs704795 | 2 | 27569998 | -0.0149 | 0.0032 | 2.44E-06 | a | g | 0.3783 | FNDC4 |
| rs17145685 | 10 | 9352066 | 0.0324 | 0.0069 | 2.44E-06 | a | c | 0.0638 |  |
| rs321521 | 3 | 25183168 | 0.0185 | 0.0039 | 2.45E-06 | t | c | 0.1869 |  |
| rs6460292 | 7 | 65447510 | 0.0145 | 0.0031 | 2.45E-06 | a | g | 0.5764 | TPST1 |
| rs10257427 | 7 | 65380643 | -0.0144 | 0.0031 | 2.46E-06 | t | c | 0.4238 | TPST1 |
| rs12269977 | 11 | 96763163 | 0.1158 | 0.0246 | 2.48E-06 | a | g | 0.012 |  |
| rs6956179 | 7 | 65444094 | 0.0145 | 0.0031 | 2.48E-06 | t | c | 0.5764 | TPST1 |
| rs2293572 | 2 | 27582281 | 0.0149 | 0.0032 | 2.50E-06 | c | g | 0.6265 | GCKR |
| rs709595 | 7 | 65454768 | -0.0145 | 0.0031 | 2.54E-06 | c | g | 0.4234 | TPST1 |
| rs2420591 | 7 | 65549816 | 0.0143 | 0.003 | 2.59E-06 | t | c | 0.5765 |  |
| rs12149780 | 16 | 83331126 | -0.0165 | 0.0035 | 2.59E-06 | t | c | 0.5884 | USP10 |
| rs10215948 | 7 | 65385221 | 0.0144 | 0.0031 | 2.63E-06 | a | g | 0.576 | TPST1 |
| rs811880 | 7 | 65456081 | 0.0144 | 0.0031 | 2.66E-06 | t | c | 0.5762 | TPST1 |
| rs9817571 | 3 | 123538670 | -0.0201 | 0.0043 | 2.75E-06 | t | c | 0.1562 | CSTA |
| rs11078597 | 17 | 1565113 | -0.0243 | 0.0052 | 2.82E-06 | t | c | 0.8191 | MGC14376 |
| rs12668936 | 7 | 65551839 | -0.0143 | 0.0031 | 2.83E-06 | t | c | 0.4226 |  |
| rs709597 | 7 | 65463418 | -0.0143 | 0.0031 | 2.86E-06 | t | c | 0.422 |  |
| rs7783613 | 7 | 65442696 | 0.0144 | 0.0031 | 2.91E-06 | t | c | 0.5771 | TPST1 |
| rs6971509 | 7 | 65352405 | 0.0142 | 0.003 | 2.93E-06 | t | g | 0.5767 | TPST1 |
| rs6945019 | 7 | 65559893 | 0.0143 | 0.0031 | 3.01E-06 | c | g | 0.5778 |  |
| rs778685 | 7 | 65473611 | -0.0143 | 0.0031 | 3.01E-06 | t | g | 0.4238 |  |
| rs12673810 | 7 | 65561288 | -0.0143 | 0.0031 | 3.01E-06 | a | t | 0.4222 |  |
| rs6945032 | 7 | 65559921 | -0.0143 | 0.0031 | 3.02E-06 | t | c | 0.4222 |  |
| rs2042133 | 7 | 65569357 | 0.0143 | 0.0031 | 3.04E-06 | a | g | 0.5777 |  |
| rs11974219 | 7 | 65284845 | 0.0142 | 0.0031 | 3.08E-06 | t | c | 0.576 |  |
| rs7447593 | 5 | 176756743 | -0.0174 | 0.0037 | 3.11E-06 | c | g | 0.651 | SLC34A1 |
| rs6460296 | 7 | 65532574 | -0.0142 | 0.003 | 3.11E-06 | t | c | 0.4229 |  |
| rs7789768 | 7 | 65576415 | 0.0143 | 0.0031 | 3.13E-06 | a | c | 0.578 |  |
| rs2077593 | 7 | 65529965 | 0.0142 | 0.003 | 3.19E-06 | a | g | 0.5771 |  |
| rs1441093 | 2 | 233937223 | 0.0164 | 0.0035 | 3.21E-06 | a | g | 0.2516 | DGKD |
| rs1643388 | 7 | 65481997 | 0.0142 | 0.0031 | 3.25E-06 | t | c | 0.5762 | LOC285908 |
| rs6862195 | 5 | 176755118 | 0.0174 | 0.0037 | 3.27E-06 | t | g | 0.3489 | SLC34A1 |
| rs778706 | 7 | 65497859 | 0.0142 | 0.0031 | 3.28E-06 | a | g | 0.5763 | LOC285908 |
| rs6420094 | 5 | 176750242 | -0.0173 | 0.0037 | 3.30E-06 | a | g | 0.6513 | SLC34A1 |
| rs1968225 | 7 | 65512208 | 0.0142 | 0.0031 | 3.31E-06 | t | c | 0.5764 |  |
| rs2877602 | 3 | 123642022 | -0.0185 | 0.004 | 3.34E-06 | t | c | 0.2128 | KPNA1 |
| rs778726 | 7 | 65466166 | -0.0143 | 0.0031 | 3.38E-06 | t | c | 0.4255 |  |
| rs1002053 | 7 | 65435980 | 0.0143 | 0.0031 | 3.47E-06 | a | g | 0.5756 | TPST1 |
| rs1436250 | 3 | 25210866 | -0.0191 | 0.0041 | 3.54E-06 | t | c | 0.8242 |  |
| rs2303369 | 2 | 27568920 | -0.0147 | 0.0032 | 3.61E-06 | t | c | 0.3739 | FNDC4 |
| rs1974769 | 7 | 65588049 | -0.0143 | 0.0031 | 3.62E-06 | a | g | 0.4175 |  |
| rs6958277 | 7 | 65616784 | 0.0141 | 0.003 | 3.68E-06 | a | g | 0.5778 |  |
| rs11765965 | 7 | 65479656 | 0.0142 | 0.0031 | 3.86E-06 | a | g | 0.5704 | LOC285908 |
| rs209956 | 20 | 52156263 | 0.0157 | 0.0034 | 3.94E-06 | a | g | 0.7204 |  |
| rs9830578 | 3 | 25236728 | 0.0187 | 0.0041 | 4.03E-06 | a | c | 0.1722 |  |
| rs2885835 | 3 | 25249254 | -0.0198 | 0.0043 | 4.17E-06 | a | g | 0.8451 |  |
| rs1042636 | 3 | 123486459 | 0.0263 | 0.0057 | 4.25E-06 | a | g | 0.9199 | CASR |
| rs7792762 | 7 | 65641573 | 0.014 | 0.0031 | 4.38E-06 | t | c | 0.5777 |  |
| rs7794930 | 7 | 65415981 | -0.0143 | 0.0031 | 4.44E-06 | t | g | 0.4214 | TPST1 |
| rs1565531 | 7 | 65300548 | 0.0141 | 0.0031 | 4.64E-06 | a | t | 0.5711 |  |
| rs668272 | 11 | 85898372 | -0.0151 | 0.0033 | 4.68E-06 | a | g | 0.6998 | ME3 |
| rs11681351 | 2 | 27596927 | -0.0144 | 0.0031 | 4.77E-06 | a | g | 0.3815 | GCKR |
| rs17054619 | 6 | 157268496 | -0.0275 | 0.006 | 4.78E-06 | t | c | 0.0704 | ARID1B |
| rs978142 | 3 | 25203993 | -0.0163 | 0.0036 | 4.93E-06 | t | c | 0.7576 |  |
