# Supplementary material for: Meta-Analysis of Genome-Wide Association Studies Identifies Six New Loci for Serum Calcium Concentrations
Source: PLoS Genet. 2013 Sep 19;9(9):e1003796. doi: 10.1371/journal.pgen.1003796 (PMC3778004; doi:10.1371/journal.pgen.1003796)
Supplement: Table S4 — Comparison of association with uncorrected versus corrected serum calcium. Chr, chromosome. Freq A1, frequency of allele A1. Beta, regression coefficient for the A1 allele. SE, standard error. A1, allele 1 (effect allele). Only replicated loci are included in this table. (DOCX) [file pgen.1003796.s012.docx]

Table S4: Comparison of association with uncorrected versus corrected serum calcium

|  |  |  |  |  |  | **primary analysis (uncorrected serum calcium)** | | | | | **secondary analysis**  **(albumin-corrected serum calcium)** | | | | |
| --- | --- | --- | --- | --- | --- | --- | --- | --- | --- | --- | --- | --- | --- | --- | --- |
| **Locus** | **SNP** | **chr** | **position** | **A1** | **A2** | **N** | **Freq A1** | **Beta** | **SE** | **P value** | **N** | **Freq A1** | **Beta** | **SE** | **P value** |
| *CASR* | *rs1801725* | 3 | 123486447 | g | t | 39400 | 0.15 | 0.0689 | 0.0043 | 6.52E-59 | 35080 | 0.15 | 0.0647 | 0.0041 | 2.37E-55 |
| *DGKD* | *rs1550532* | 2 | 233929587 | c | g | 39400 | 0.31 | 0.0179 | 0.0033 | 4.60E-08 | 35080 | 0.31 | 0.0175 | 0.0032 | 4.19E-08 |
| GCKR | *rs780094* | 2 | 27594741 | t | c | 39400 | 0.41 | 0.0204 | 0.0031 | 3.69E-11 | 35080 | 0.42 | 0.0117 | 0.003 | 9.74E-05 |
| GATA3 | rs10491003 | 10 | 9368657 | c | t | 38361 | 0.09 | 0.0267 | 0.0056 | 1.59E-06 | 34041 | 0.09 | 0.0229 | 0.0054 | 2.48E-05 |
| CARS | rs7481584 | 11 | 2985665 | g | a | 39400 | 0.29 | -0.021 | 0.0034 | 9.21E-10 | 35080 | 0.29 | -0.0207 | 0.0033 | 3.89E-10 |
| DGKH;KIAA0564 | rs7336933 | 13 | 41457076 | g | a | 39400 | 0.15 | -0.023 | 0.0043 | 1.61E-07 | 35080 | 0.15 | -0.0205 | 0.0042 | 1.11E-06 |
| CYP24A1 | rs1570669 | 20 | 52207834 | g | a | 39400 | 0.66 | -0.018 | 0.0032 | 3.98E-08 | 35080 | 0.66 | -0.0154 | 0.0032 | 1.17E-06 |
