# Supplementary material for: Meta-Analysis of Genome-Wide Association Studies Identifies Six New Loci for Serum Calcium Concentrations
Source: PLoS Genet. 2013 Sep 19;9(9):e1003796. doi: 10.1371/journal.pgen.1003796 (PMC3778004; doi:10.1371/journal.pgen.1003796)
Supplement: Table S5 — Genome-wide significant loci for corrected calcium in Europeans (discovery). Chr, chromosome. Position, position on build 36. A1, allele 1 (effect allele). A2, allele 2. Freq A1, frequency of allele 1. InRefGen, gene symbol if SNP is located within a specific gene. (DOCX) [file pgen.1003796.s013.docx]

## Table S5: Genome-wide significant loci for corrected calcium in Europeans (discovery)

| **SNP** | **chr** | **position** | **Effect A1** | **SE** | **P.value** | **A1** | **A2** | **Freq A1** | **InRefGene** |
| --- | --- | --- | --- | --- | --- | --- | --- | --- | --- |
| rs17251221 | 3 | 123475937 | -0.0665 | 0.0042 | 9.36E-56 | a | g | 0.8472 | CASR |
| rs1801725 | 3 | 123486447 | 0.0647 | 0.0041 | 2.37E-55 | t | g | 0.1536 | CASR |
| rs17265703 | 3 | 123531334 | -0.058 | 0.004 | 8.38E-48 | a | g | 0.8336 | CSTA |
| rs5008830 | 3 | 123513152 | 0.0579 | 0.004 | 4.48E-47 | a | g | 0.1662 |  |
| rs2001548 | 3 | 123515479 | 0.0572 | 0.004 | 2.56E-46 | a | g | 0.1667 |  |
| rs16832956 | 3 | 123500198 | -0.0471 | 0.0037 | 4.65E-38 | c | g | 0.794 |  |
| rs16833080 | 3 | 123583597 | 0.0503 | 0.0041 | 1.17E-34 | t | c | 0.1551 | CCDC58 |
| rs16833078 | 3 | 123582284 | -0.05 | 0.0041 | 2.06E-34 | a | g | 0.8446 | CCDC58 |
| rs6791616 | 3 | 123589925 | 0.0478 | 0.0039 | 4.26E-34 | t | c | 0.169 | C3orf28 |
| rs12107092 | 3 | 123606538 | 0.048 | 0.0039 | 4.67E-34 | t | c | 0.1681 | C3orf28 |
| rs9834317 | 3 | 123572049 | 0.0495 | 0.0041 | 4.70E-34 | t | g | 0.1558 | CCDC58 |
| rs6438725 | 3 | 123570796 | -0.0494 | 0.0041 | 6.74E-34 | t | c | 0.8441 | CCDC58 |
| rs4491840 | 3 | 123563437 | 0.0488 | 0.004 | 1.59E-33 | a | g | 0.1567 | CCDC58 |
| rs17200894 | 3 | 123612831 | -0.0468 | 0.0041 | 1.06E-30 | c | g | 0.8395 |  |
| rs17201246 | 3 | 123628160 | 0.0464 | 0.0041 | 2.81E-30 | a | c | 0.1606 | KPNA1 |
| rs9789994 | 3 | 123700541 | 0.0461 | 0.0041 | 6.30E-30 | a | t | 0.1602 | KPNA1 |
| rs16833168 | 3 | 123737337 | 0.0463 | 0.0041 | 7.07E-30 | t | c | 0.1591 | PARP9 |
| rs2270859 | 3 | 123738398 | 0.0462 | 0.0041 | 1.08E-29 | a | g | 0.1589 | PARP9 |
| rs17267388 | 3 | 123750236 | 0.0445 | 0.004 | 4.77E-29 | a | g | 0.1691 | PARP9 |
| rs11929034 | 3 | 123761739 | 0.0466 | 0.0042 | 1.08E-28 | a | g | 0.161 | PARP9 |
| rs10222633 | 3 | 123459616 | -0.0329 | 0.003 | 1.09E-28 | a | g | 0.485 | CASR |
| rs3749203 | 3 | 123459184 | -0.0333 | 0.0031 | 3.17E-27 | t | c | 0.4856 | CASR |
| rs4306808 | 3 | 123611156 | -0.0569 | 0.0055 | 4.62E-25 | c | g | 0.8946 | C3orf28 |
| rs17266816 | 3 | 123611564 | 0.0562 | 0.0055 | 2.20E-24 | a | g | 0.1052 | C3orf28 |
| rs1067 | 3 | 123615655 | 0.0385 | 0.0039 | 4.50E-23 | a | g | 0.201 | WDR5B |
| rs4678192 | 3 | 123610282 | -0.0305 | 0.0031 | 5.64E-23 | a | g | 0.6114 | C3orf28 |
| rs13095172 | 3 | 123472947 | 0.0305 | 0.0032 | 3.32E-22 | t | c | 0.3362 | CASR |
| rs3792289 | 3 | 123476290 | -0.0305 | 0.0032 | 3.56E-22 | a | g | 0.6637 | CASR |
| rs10934578 | 3 | 123459972 | 0.0302 | 0.0031 | 4.27E-22 | t | g | 0.3361 | CASR |
| rs3749208 | 3 | 123462974 | 0.0302 | 0.0031 | 6.09E-22 | t | c | 0.3363 | CASR |
| rs13085674 | 3 | 123494041 | 0.0303 | 0.0032 | 1.04E-21 | a | g | 0.3377 |  |
| rs13085498 | 3 | 123494053 | 0.0303 | 0.0032 | 1.07E-21 | t | c | 0.3377 |  |
| rs10934582 | 3 | 123496044 | 0.0301 | 0.0032 | 2.73E-21 | a | g | 0.3395 |  |
| rs6768471 | 3 | 123462394 | -0.0289 | 0.0031 | 1.05E-20 | a | g | 0.3548 | CASR |
| rs11711910 | 3 | 123468572 | 0.0286 | 0.0031 | 2.51E-20 | t | g | 0.6459 | CASR |
| rs11716910 | 3 | 123470309 | 0.0286 | 0.0031 | 2.64E-20 | a | g | 0.646 | CASR |
| rs7644390 | 3 | 123472918 | -0.0285 | 0.0031 | 3.00E-20 | a | t | 0.3548 | CASR |
| rs4678173 | 3 | 123473829 | -0.0285 | 0.0031 | 3.06E-20 | a | c | 0.3548 | CASR |
| rs11922857 | 3 | 123480078 | -0.0285 | 0.0031 | 3.43E-20 | a | g | 0.3546 | CASR |
| rs7646147 | 3 | 123489217 | 0.028 | 0.0031 | 1.36E-19 | t | c | 0.65 |  |
| rs9740 | 3 | 123487743 | 0.028 | 0.0031 | 1.41E-19 | a | g | 0.6498 | CASR |
| rs10934581 | 3 | 123493844 | -0.028 | 0.0031 | 2.08E-19 | t | c | 0.3498 |  |
| rs13083990 | 3 | 123497256 | -0.0283 | 0.0031 | 2.36E-19 | t | c | 0.6445 |  |
| rs7633800 | 3 | 123494355 | -0.0278 | 0.0031 | 3.86E-19 | a | g | 0.3495 |  |
| rs7633667 | 3 | 123494416 | 0.0278 | 0.0031 | 4.36E-19 | c | g | 0.6504 |  |
| rs1127343 | 3 | 123611084 | -0.028 | 0.0031 | 4.67E-19 | a | g | 0.6056 | C3orf28 |
| rs12635478 | 3 | 123491243 | 0.0287 | 0.0033 | 1.20E-18 | a | c | 0.3261 |  |
| rs11720638 | 3 | 123495657 | -0.0275 | 0.0031 | 1.91E-18 | c | g | 0.3499 |  |
| rs9856248 | 3 | 123546501 | 0.0291 | 0.0033 | 2.22E-18 | t | c | 0.2729 |  |
| rs11926904 | 3 | 123428845 | 0.0638 | 0.0075 | 2.16E-17 | t | g | 0.0465 | CASR |
| rs9864290 | 3 | 123522752 | -0.0275 | 0.0032 | 2.18E-17 | t | c | 0.707 |  |
| rs6438720 | 3 | 123499922 | 0.0264 | 0.0031 | 2.38E-17 | a | c | 0.4122 |  |
| rs4678180 | 3 | 123520487 | 0.0275 | 0.0032 | 2.40E-17 | t | c | 0.2932 |  |
| rs6803098 | 3 | 123523300 | -0.0274 | 0.0032 | 3.13E-17 | t | c | 0.707 |  |
| rs12185943 | 3 | 123498259 | 0.0262 | 0.0032 | 9.41E-17 | a | g | 0.6584 |  |
| rs9851884 | 3 | 123502884 | 0.0266 | 0.0033 | 2.85E-16 | a | g | 0.2851 |  |
| rs16833133 | 3 | 123627563 | 0.058 | 0.0073 | 1.79E-15 | a | g | 0.0621 | KPNA1 |
| rs16833165 | 3 | 123730363 | -0.058 | 0.0073 | 2.25E-15 | t | c | 0.9381 | PARP9 |
| rs17252533 | 3 | 123504897 | -0.0248 | 0.0031 | 3.20E-15 | t | g | 0.3344 |  |
| rs2001547 | 3 | 123515524 | -0.0247 | 0.0031 | 4.09E-15 | a | g | 0.3329 |  |
| rs3804590 | 3 | 123460763 | 0.0284 | 0.0036 | 4.12E-15 | t | g | 0.3328 | CASR |
| rs1472621 | 3 | 123495416 | -0.0282 | 0.0037 | 1.83E-14 | a | g | 0.6662 |  |
| rs1402200 | 3 | 123505107 | -0.0246 | 0.0032 | 2.29E-14 | c | g | 0.6955 |  |
| rs13320117 | 3 | 123610512 | -0.0273 | 0.0036 | 3.55E-14 | t | c | 0.6406 | C3orf28 |
| rs6798997 | 3 | 123557394 | -0.0214 | 0.003 | 1.06E-12 | a | g | 0.4099 |  |
| rs6768280 | 3 | 123607742 | -0.0211 | 0.003 | 2.29E-12 | a | g | 0.4093 | C3orf28 |
| rs9818363 | 3 | 123594981 | 0.0207 | 0.003 | 3.89E-12 | t | c | 0.573 | C3orf28 |
| rs12487598 | 3 | 123661292 | 0.0207 | 0.003 | 7.40E-12 | t | c | 0.5916 | KPNA1 |
| rs6438737 | 3 | 123711664 | -0.0201 | 0.003 | 2.52E-11 | t | c | 0.4107 | KPNA1 |
| rs16833051 | 3 | 123550948 | -0.0223 | 0.0034 | 2.73E-11 | a | g | 0.2735 |  |
| rs10511410 | 3 | 123611209 | -0.022 | 0.0033 | 3.15E-11 | c | g | 0.2943 | C3orf28 |
| rs4678191 | 3 | 123603928 | 0.0202 | 0.0031 | 4.73E-11 | t | g | 0.5303 | C3orf28 |
| rs4315641 | 3 | 123551565 | -0.022 | 0.0034 | 4.92E-11 | a | t | 0.2753 |  |
| rs11763147 | 7 | 64964256 | 0.0194 | 0.003 | 5.55E-11 | a | g | 0.4443 |  |
| rs7791814 | 7 | 65002924 | -0.0194 | 0.003 | 5.87E-11 | t | c | 0.5557 | VKORC1L1 |
| rs4718286 | 7 | 64930199 | 0.0195 | 0.003 | 5.94E-11 | a | g | 0.4454 |  |
| rs4502988 | 7 | 64935181 | 0.0195 | 0.003 | 5.96E-11 | a | g | 0.4454 |  |
| rs17711722 | 7 | 64908632 | 0.0199 | 0.003 | 5.97E-11 | t | c | 0.4732 |  |
| rs11920570 | 3 | 123572792 | -0.0218 | 0.0033 | 7.97E-11 | a | g | 0.2734 | CCDC58 |
| rs12633068 | 3 | 123578264 | 0.0217 | 0.0033 | 8.71E-11 | c | g | 0.7266 | CCDC58 |
| rs1829943 | 7 | 64913843 | 0.0195 | 0.003 | 9.75E-11 | a | g | 0.4456 |  |
| rs7784623 | 7 | 65032469 | 0.0191 | 0.003 | 1.04E-10 | t | g | 0.444 | VKORC1L1 |
| rs6960048 | 7 | 65045474 | 0.0191 | 0.003 | 1.08E-10 | a | g | 0.444 | VKORC1L1 |
| rs6947132 | 7 | 64910930 | 0.0194 | 0.003 | 1.10E-10 | a | g | 0.446 |  |
| rs7618100 | 3 | 123432810 | -0.0215 | 0.0033 | 1.12E-10 | c | g | 0.7118 | CASR |
| rs1917563 | 7 | 65053082 | 0.0191 | 0.003 | 1.19E-10 | a | g | 0.444 | VKORC1L1 |
| rs10807697 | 7 | 65053605 | 0.0191 | 0.003 | 1.23E-10 | a | g | 0.444 | VKORC1L1 |
| rs9811123 | 3 | 123431982 | -0.0214 | 0.0033 | 1.25E-10 | a | g | 0.7117 | CASR |
| rs9866419 | 3 | 123422899 | -0.0214 | 0.0033 | 1.28E-10 | a | g | 0.7117 | CASR |
| rs9530 | 7 | 65063329 | 0.019 | 0.003 | 1.49E-10 | a | g | 0.4437 | GUSB |
| rs1631091 | 7 | 65103375 | 0.019 | 0.003 | 1.50E-10 | t | g | 0.4444 |  |
| rs781143 | 7 | 65077314 | -0.019 | 0.003 | 1.51E-10 | c | g | 0.5557 | GUSB |
| rs1723268 | 7 | 65110515 | 0.019 | 0.003 | 1.53E-10 | a | c | 0.4444 |  |
| rs2658585 | 7 | 65099376 | 0.019 | 0.003 | 1.55E-10 | a | t | 0.4443 |  |
| rs1701760 | 7 | 65111123 | 0.019 | 0.003 | 1.66E-10 | a | g | 0.4444 |  |
| rs2690442 | 7 | 64873311 | 0.0191 | 0.003 | 2.10E-10 | t | c | 0.4462 |  |
| rs4718278 | 7 | 64881031 | 0.0191 | 0.003 | 2.24E-10 | t | c | 0.4463 |  |
| rs7481584 | 11 | 2985665 | -0.0207 | 0.0033 | 3.89E-10 | a | g | 0.2948 | CARS |
| rs729662 | 11 | 2984716 | -0.0207 | 0.0033 | 3.91E-10 | a | g | 0.2948 | CARS |
| rs11768292 | 7 | 65112236 | 0.019 | 0.0031 | 6.05E-10 | t | g | 0.4053 |  |
| rs3847646 | 11 | 2910299 | -0.0258 | 0.0042 | 9.13E-10 | t | c | 0.2237 |  |
| rs7635930 | 3 | 123595247 | -0.0203 | 0.0033 | 9.64E-10 | c | g | 0.2689 | C3orf28 |
| rs6438726 | 3 | 123593611 | -0.0203 | 0.0033 | 1.01E-09 | t | c | 0.2691 | C3orf28 |
| rs1986617 | 3 | 123599247 | -0.0199 | 0.0033 | 1.01E-09 | a | t | 0.2851 | C3orf28 |
| rs2583435 | 11 | 2915394 | 0.0198 | 0.0033 | 1.43E-09 | t | c | 0.6975 |  |
| rs3814964 | 11 | 2956083 | -0.0195 | 0.0032 | 1.98E-09 | t | g | 0.3025 | NAP1L4 |
| rs12805915 | 11 | 2926347 | -0.0196 | 0.0033 | 1.99E-09 | a | g | 0.302 | NAP1L4 |
| rs2071110 | 11 | 2947795 | 0.0195 | 0.0033 | 2.02E-09 | c | g | 0.6913 | NAP1L4 |
| rs4758502 | 11 | 2955037 | -0.0195 | 0.0032 | 2.07E-09 | t | c | 0.3026 | NAP1L4 |
| rs11024759 | 11 | 2938668 | 0.0194 | 0.0033 | 2.58E-09 | t | c | 0.6915 | NAP1L4 |
| rs2001006 | 11 | 2931488 | 0.0194 | 0.0033 | 2.74E-09 | t | c | 0.692 | NAP1L4 |
| rs1468102 | 11 | 2961102 | 0.019 | 0.0032 | 3.31E-09 | c | g | 0.6827 | NAP1L4 |
| rs756693 | 11 | 2984465 | 0.0206 | 0.0035 | 3.35E-09 | a | g | 0.7225 | CARS |
| rs12806061 | 11 | 2964980 | -0.019 | 0.0032 | 3.40E-09 | a | g | 0.3172 | NAP1L4 |
| rs4758621 | 11 | 2966216 | 0.0189 | 0.0032 | 3.80E-09 | a | g | 0.6828 | NAP1L4 |
| rs7648255 | 3 | 123650370 | -0.0197 | 0.0033 | 3.90E-09 | a | t | 0.2667 | KPNA1 |
| rs9875101 | 3 | 123450780 | 0.0191 | 0.0033 | 4.24E-09 | t | c | 0.3007 | CASR |
| rs2173961 | 3 | 123451537 | 0.0192 | 0.0033 | 4.29E-09 | t | g | 0.3002 | CASR |
| rs4677951 | 3 | 123641930 | 0.0196 | 0.0033 | 4.60E-09 | c | g | 0.7331 | KPNA1 |
| rs1979869 | 3 | 123445975 | 0.0189 | 0.0032 | 4.98E-09 | t | c | 0.3017 | CASR |
| rs12493480 | 3 | 123445653 | -0.0189 | 0.0032 | 5.11E-09 | a | g | 0.6983 | CASR |
| rs2134221 | 3 | 123444936 | -0.0189 | 0.0032 | 5.34E-09 | a | g | 0.698 | CASR |
| rs6968619 | 7 | 65706302 | -0.0172 | 0.0029 | 5.61E-09 | t | c | 0.4942 |  |
| rs7635354 | 3 | 123449328 | -0.019 | 0.0033 | 5.68E-09 | a | c | 0.699 | CASR |
| rs3863977 | 3 | 123446952 | 0.0189 | 0.0032 | 5.73E-09 | t | c | 0.3013 | CASR |
| rs13326577 | 3 | 123447936 | 0.0189 | 0.0032 | 5.94E-09 | a | t | 0.3013 | CASR |
| rs1965358 | 3 | 123452991 | -0.019 | 0.0033 | 6.07E-09 | a | g | 0.6991 | CASR |
| rs1463892 | 3 | 123453076 | 0.0191 | 0.0033 | 6.22E-09 | a | g | 0.3009 | CASR |
| rs10258739 | 7 | 65700370 | -0.0171 | 0.0029 | 6.61E-09 | a | t | 0.4948 |  |
| rs6769837 | 3 | 123680674 | 0.0193 | 0.0033 | 6.76E-09 | a | g | 0.7319 | KPNA1 |
| rs429289 | 11 | 3029152 | -0.0226 | 0.0039 | 7.24E-09 | t | c | 0.2614 | CARS |
| rs801191 | 7 | 65670390 | 0.0171 | 0.003 | 7.59E-09 | a | g | 0.5067 |  |
| rs6805271 | 3 | 123695649 | 0.0192 | 0.0033 | 7.64E-09 | t | c | 0.732 | KPNA1 |
| rs6780306 | 3 | 123708144 | 0.0191 | 0.0033 | 9.23E-09 | t | c | 0.731 | KPNA1 |
| rs937626 | 3 | 123454492 | -0.0194 | 0.0034 | 1.15E-08 | a | g | 0.6952 | CASR |
| rs6780909 | 3 | 123732854 | 0.0186 | 0.0033 | 1.82E-08 | t | g | 0.7306 | PARP9 |
| rs1042636 | 3 | 123486459 | 0.0313 | 0.0056 | 2.13E-08 | a | g | 0.9202 | CASR |
| rs6808422 | 3 | 123499876 | -0.0171 | 0.0031 | 2.72E-08 | t | c | 0.4871 |  |
| rs1167406 | 7 | 65194371 | 0.0168 | 0.003 | 2.93E-08 | c | g | 0.6195 | ASL |
| rs1167390 | 7 | 65213328 | 0.0167 | 0.003 | 2.96E-08 | a | g | 0.6185 |  |
| rs709609 | 7 | 65197996 | -0.0167 | 0.003 | 3.15E-08 | a | g | 0.381 |  |
| rs6460293 | 7 | 65447627 | 0.0168 | 0.003 | 3.32E-08 | a | c | 0.6171 | TPST1 |
| rs4678176 | 3 | 123491865 | 0.0307 | 0.0056 | 3.50E-08 | a | g | 0.92 |  |
| rs1983372 | 7 | 65248786 | 0.0166 | 0.003 | 3.76E-08 | t | c | 0.6173 | RCP9 |
| rs1550532 | 2 | 233929587 | 0.0175 | 0.0032 | 4.19E-08 | c | g | 0.311 | DGKD |
| rs587360 | 7 | 65160133 | -0.0165 | 0.003 | 4.28E-08 | a | c | 0.4397 |  |
| rs908915 | 7 | 65252086 | 0.0165 | 0.003 | 4.47E-08 | t | g | 0.6172 | RCP9 |
| rs778736 | 7 | 65451283 | -0.0167 | 0.003 | 4.54E-08 | t | c | 0.3831 | TPST1 |
| rs6959268 | 7 | 65450401 | 0.0167 | 0.003 | 4.59E-08 | a | g | 0.6169 | TPST1 |
| rs7783779 | 7 | 65434061 | -0.0166 | 0.003 | 4.77E-08 | a | g | 0.3813 | TPST1 |
| rs10447522 | 7 | 65433509 | -0.0166 | 0.003 | 4.88E-08 | a | g | 0.3816 | TPST1 |
| rs2249458 | 7 | 65202509 | 0.0166 | 0.003 | 4.92E-08 | t | c | 0.6157 |  |
