# Supplementary material for: Meta-Analysis of Genome-Wide Association Studies Identifies Six New Loci for Serum Calcium Concentrations
Source: PLoS Genet. 2013 Sep 19;9(9):e1003796. doi: 10.1371/journal.pgen.1003796 (PMC3778004; doi:10.1371/journal.pgen.1003796)
Supplement: Table S6 — eQTL analysis for the seven genome-wide replicated loci for serum calcium. We used the online eQTL database of the University of Chicago (http://eqtl.uchicago.edu/cgi-bin/gbrowse/eqtl/., last accessed, November 5, 2012). All eQTL were acting in cis. (DOCX) [file pgen.1003796.s014.docx]

## Table S6: eQTL analysis for the seven genome-wide replicated loci for serum calcium.

| **Serum calcium SNP** | **eSNP** | **r^2^ between serum calcium SNP and eSNP** | **reference** | **PMID** | **Tissue** | **Significance criteria for expression** | **Transcript(s)** | **chr: position (b36)** |
| --- | --- | --- | --- | --- | --- | --- | --- | --- |
| rs1801725 | rs17265703 | 0.929 | Zeller 2010[[1](#_ENREF_1)] | PMC2872668 | circulating monocytes | P<5.78×10^−12^ | CSTA | [chr3:123531334..123531334](http://eqtl.uchicago.edu/cgi-bin/gbrowse/eqtl?name=chr3:123531334..123531334) |
| rs1801725 | rs5008830 | 1 | Zeller 2010[[1](#_ENREF_1)] | PMC2872668 | circulating monocytes | P<5.78×10^−12^ | CSTA | [chr3:123513152..123513152](http://eqtl.uchicago.edu/cgi-bin/gbrowse/eqtl?name=chr3:123513152..123513152) |
| rs7481584 | rs2583435 | 1 | Innocenti 2011[[2](#_ENREF_2)] | 21637794 | liver | Bayes Factor >5 | NAP1L4 | [chr11:2915394..2915394](http://eqtl.uchicago.edu/cgi-bin/gbrowse/eqtl?name=chr11:2915394..2915394) |
| rs7481584 | rs4758621 | 0.961 | Zeller 2010[[1](#_ENREF_1)] | PMC2872668 | circulating monocytes | P<5.78×10^−12^ | SLC22A18; SLC22A18AS | [chr11:2966216..2966216](http://eqtl.uchicago.edu/cgi-bin/gbrowse/eqtl?name=chr11:2966216..2966216) |
| rs780094 | rs1260333 | 0.87 | Veyrieras 2008[[3](#_ENREF_3)] | PMC2556086 | lymphoblastoid cell lines | P<7×10^−6^ | IKZF3; ORMDL3 | [chr17:35336333..35336333](http://eqtl.uchicago.edu/cgi-bin/gbrowse/eqtl?name=chr17:35336333..35336333) |
