# Supplementary material for: Meta-Analysis of Genome-Wide Association Studies Identifies Six New Loci for Serum Calcium Concentrations
Source: PLoS Genet. 2013 Sep 19;9(9):e1003796. doi: 10.1371/journal.pgen.1003796 (PMC3778004; doi:10.1371/journal.pgen.1003796)
Supplement: Table S7 — Details on genes located in the GCKR genomic region. (DOCX) [file pgen.1003796.s015.docx]

## Table S7: Details on genes located in the GCKR genomic region

***Chromosome 2, locus rs780094***

***GCKR*** : *GCKR* encodes glucokinase (hexokinase 4) regulator, which inhibits hepatic glucokinase activity. The product of this gene therefore plays a key role in glucose homeostasis. rs780094 is located in intro 16 of the *GCKR* gene and is in strong linkage disequilibrium (r2=0.93[^1^](#_ENREF_1)), in Caucasians, with a common non-synonymous SNP (P446L, rs1260326) associated with glucokinase activity *in vitro*[*^1^*](#_ENREF_1)*^,^*[*^2^*](#_ENREF_2). The *GCKR* gene is known for its pleiotropic effect.[^3^](#_ENREF_3) GCKR variants have been associated with multiple metabolic alterations, including blood lipid concentrations,[^4^](#_ENREF_4)^,^[^5^](#_ENREF_5) type 2 diabetes and fasting glucose concentrations,[^6^](#_ENREF_6) inflammatory markers and diseases),[^7^](#_ENREF_7)^,^[^8^](#_ENREF_8) circulating amino acids,[^9^](#_ENREF_9) liver enzymes,[^10^](#_ENREF_10) serum urate,[^11^](#_ENREF_11) serum albumin[^12^](#_ENREF_12) as well as renal function.[^13^](#_ENREF_13)^,^[^14^](#_ENREF_14)

***FNDC4*** encodes fibronectin type III domain containing protein 4. It belongs to the fibronectin family of glycoproteins, which play a role in extracellular matrix.

***IFT172*** encodes intraflagellar transport 172 homolog. The gene product is involved in genesis and maintenance of primary cilia.[^15^](#_ENREF_15) There is no known direct link with calcium homeostasis.

***KRTCAP3*** encodes keratinocyte associated protein 3. Little is known about the function of this gene. There is no known direct link with calcium homeostasis.

***NRBP1*** encodes nuclear receptor binding protein 1. This is a tumor suppressor gene.[^16^](#_ENREF_16)

***FTHL3P*** encodes ferritin, heavy polypeptide 1 pseudogene 3.

***PPM1G*** encodes protein phosphatase, Mg2+/Mn2+ dependent, 1G, a member of the PP2C family of Ser/Thr protein phosphatases involved in cell stress response pathways. This enzyme could regulate cell cycle progression. There is no known direct link with calcium homeostasis.

***SNX17*** encodes sorting nexin 17, involved in intracellular trafficking. SNX17 has been identified as a β1-integrin-tail-binding protein that promotes the recycling of β1-integrins to the cell surface.[^17^](#_ENREF_17). There is no known direct link with calcium homeostasis.

***EIF2B4*** encodes eukaryotic translation initiation factor 2B, subunit 4 delta, 67kDa, involved in protein synthesis. Mutations in this gene are responsible for Vanishing white matter disease (OMIM #603896), an autosomal recessive transmitted leukoencephalopathy.[^18^](#_ENREF_18) There is no known direct link with calcium homeostasis.

***GTF3C2*** encodes general transcription factor IIIC, polypeptide 2, beta. There is no known direct link with calcium homeostasis.

***ZNF513*** encodes zinc finger protein 513. This gene, expressed in the retina, is involved in autosomal-recessive retinitis pigmentosa.[^19^](#_ENREF_19) There is no known direct link with calcium homeostasis.

***MPV17*** encodes MpV17 mitochondrial inner membrane protein, implicated in the metabolism of reactive oxygen species. This gene is responsible for the hepatocerebral form of mitochondrial DNA depletion syndrome (MDDS) (OMIM#256810), characterized by early progressive liver failure, ataxia, hypotonia, dystonia, systemic infections, poor weight gain, short stature and psychomotor regression.[^20^](#_ENREF_20)^,^[^21^](#_ENREF_21)

***UCN*** encodes urocortin, a member of the sauvagine/corticotropin-releasing factor/urotensin I family. Urocortins are members of the corticotrophin-releasing factor (CRF) family of peptides and they have vasodilatory properties by modulating vascular smooth muscle cells.[^22^](#_ENREF_22) Urocortin appears to be involved in osteoclasts’ differentiation and influence bone resorption.[^23^](#_ENREF_23)

***TRIM54*** encodes tripartite motif-containing protein 54. This gene is also called MURF for muscle-specific RING finger protein. The gene product is believe to play a role in microtubule stability in striated muscles.[^24^](#_ENREF_24)

***DNAJC5G*** encodes DnaJ (Hsp40) homolog, subfamily C, member 5 gamma, which belongs to proteins interacting selectively with a heat shock protein.[^25^](#_ENREF_25)^,^[^26^](#_ENREF_26) There is no known direct link with calcium homeostasis.

***C2orf16*** encodes chromosome 2 open reading frame 16. Nothing is known about this transcript.

***ZNF512*** encodes zink finger protein 512. Zinc finger-containing proteins are involved in a variety of processes, including DNA recognition, RNA packaging, transcriptional activation, regulation of apoptosis, protein folding and assembly, and lipid binding. Little is known on this specific gene. There is no known direct link with calcium homeostasis.

***CCDC121*** encodes coiled-coil domain containing 121. Little is known on this specific gene. There is no known direct link with calcium homeostasis.

***GPN1*** encodes GNP-loop GTPase 1, a guanosine triphosphatase enzyme that interacts with RNA polymerase II.[^27^](#_ENREF_27) There is no known direct link with calcium homeostasis.

***SUPT7L*** encodes suppressor of Ty 7 (S. cerevisiae)-like, a protein of the human STAGA complex, which modifies chromatin. There is no known direct link with calcium homeostasis.

***SLC4A1AP*** encodes solute carrier family 4 (anion exchanger), member 1, adaptor protein, expressed in the collecting tubule in mouse.[^28^](#_ENREF_28) There is no known direct link with calcium homeostasis.

References

1. Orho-Melander, M. et al. Common missense variant in the glucokinase regulatory protein gene is associated with increased plasma triglyceride and C-reactive protein but lower fasting glucose concentrations. *Diabetes* **57**, 3112-21 (2008).

2. Beer, N.L. et al. The P446L variant in GCKR associated with fasting plasma glucose and triglyceride levels exerts its effect through increased glucokinase activity in liver. *Hum Mol Genet* **18**, 4081-8 (2009).

3. Huang, J., Johnson, A.D. & O'Donnell, C.J. PRIMe: a method for characterization and evaluation of pleiotropic regions from multiple genome-wide association studies. *Bioinformatics* **27**, 1201-6 (2011).

4. Johansen, C.T. et al. Excess of rare variants in genes identified by genome-wide association study of hypertriglyceridemia. *Nature Genetics* **42**, 684-7 (2010).

5. Teslovich, T.M. et al. Biological, clinical and population relevance of 95 loci for blood lipids. *Nature* **466**, 707-13 (2010).

6. Beer, N.L. et al. The P446L variant in GCKR associated with fasting plasma glucose and triglyceride levels exerts its effect through increased glucokinase activity in liver. *Human molecular genetics* **18**, 4081-8 (2009).

7. Umeno, J. et al. Meta-analysis of published studies identified eight additional common susceptibility loci for Crohn's disease and ulcerative colitis. *Inflammatory bowel diseases* **17**, 2407-15 (2011).

8. Dupuis, J. et al. New genetic loci implicated in fasting glucose homeostasis and their impact on type 2 diabetes risk. *Nature Genetics* **42**, 105-16 (2010).

9. Kettunen, J. et al. Genome-wide association study identifies multiple loci influencing human serum metabolite levels. *Nature Genetics* **44**, 269-76 (2012).

10. Chambers, J.C. et al. Genome-wide association study identifies loci influencing concentrations of liver enzymes in plasma. *Nature Genetics* **43**, 1131-8 (2011).

11. Yang, Q. et al. Multiple genetic loci influence serum urate levels and their relationship with gout and cardiovascular disease risk factors. *Circulation. Cardiovascular genetics* **3**, 523-30 (2010).

12. Franceschini, N. et al. Discovery and Fine Mapping of Serum Protein Loci through Transethnic Meta-analysis. *Am J Hum Genet* **91**, 744-753 (2012).

13. Boger, C.A. et al. Association of eGFR-Related Loci Identified by GWAS with Incident CKD and ESRD. *PLoS genetics* **7**, e1002292 (2011).

14. Kottgen, A. et al. New loci associated with kidney function and chronic kidney disease. *Nature Genetics* **42**, 376-84 (2010).

15. Ocbina, P.J., Eggenschwiler, J.T., Moskowitz, I. & Anderson, K.V. Complex interactions between genes controlling trafficking in primary cilia. *Nat Genet* **43**, 547-53 (2011).

16. Wilson, C.H. et al. Nuclear receptor binding protein 1 regulates intestinal progenitor cell homeostasis and tumour formation. *EMBO J* **31**, 2486-97 (2012).

17. Bottcher, R.T. et al. Sorting nexin 17 prevents lysosomal degradation of beta1 integrins by binding to the beta1-integrin tail. *Nat Cell Biol* **14**, 584-92 (2012).

18. Scali, O., Di Perri, C. & Federico, A. The spectrum of mutations for the diagnosis of vanishing white matter disease. *Neurol Sci* **27**, 271-7 (2006).

19. Li, L. et al. A mutation in ZNF513, a putative regulator of photoreceptor development, causes autosomal-recessive retinitis pigmentosa. *Am J Hum Genet* **87**, 400-9 (2010).

20. Navarro-Sastre, A. et al. Functional splicing assay supporting that c.70 + 5G > A mutation in the MPV17 gene is disease causing. *J Inherit Metab Dis* (2010).

21. Singleton, R. et al. Neuropathy in Navajo children: clinical and epidemiologic features. *Neurology* **40**, 363-7 (1990).

22. Kageyama, K., Teui, K., Tamasawa, N. & Suda, T. Regulation and roles of urocortins in the vascular system. *Int J Endocrinol* **2012**, 873723 (2012).

23. Combs, C.E. et al. Urocortin is a novel regulator of osteoclast differentiation and function through inhibition of a canonical transient receptor potential 1-like cation channel. *J Endocrinol* **212**, 187-97 (2012).

24. Centner, T. et al. Identification of muscle specific ring finger proteins as potential regulators of the titin kinase domain. *J Mol Biol* **306**, 717-26 (2001).

25. Tobaben, S. et al. A trimeric protein complex functions as a synaptic chaperone machine. *Neuron* **31**, 987-99 (2001).

26. McClintick, C.A., Theisen, C.S., Ferns, J.E., Fibuch, E.E. & Seidler, N.W. Isoflurane preconditioning involves upregulation of molecular chaperone genes. *Biochem Biophys Res Commun* **411**, 387-92 (2011).

27. Reyes-Pardo, H. et al. A nuclear export sequence in GPN-loop GTPase 1, an essential protein for nuclear targeting of RNA polymerase II, is necessary and sufficient for nuclear export. *Biochim Biophys Acta* **1823**, 1756-66 (2012).

28. Chen, J., Vijayakumar, S., Li, X. & Al-Awqati, Q. Kanadaptin is a protein that interacts with the kidney but not the erythroid form of band 3. *J Biol Chem* **273**, 1038-43 (1998).
