# Supplementary material for: Meta-Analysis of Genome-Wide Association Studies Identifies Six New Loci for Serum Calcium Concentrations
Source: PLoS Genet. 2013 Sep 19;9(9):e1003796. doi: 10.1371/journal.pgen.1003796 (PMC3778004; doi:10.1371/journal.pgen.1003796)
Supplement: Table S8 — Gene Ontology classification (AmiGo). Data are GO numbers, ontology and mechanism/location from the AmiGo 1.8 gene ontology database for each gene located within ±250 kb of the seven replicated SNPs, including rs1801725 (CASR). (DOCX) [file pgen.1003796.s016.docx]

## Table S8: Gene Ontology classification (AmiGo)

| **Gene** | **GO number** | **Ontology** | **Mechanism/location** |
| --- | --- | --- | --- |
| ATG16L1 | 0000045 | biological process | autophagic vacuole assembly |
| ATG16L1 | 0051260 | biological process | protein homooligomerization |
| ATG16L1 | 0015031 | biological process | protein transport |
| ATG16L1 | 0005776 | cellular component | autophagic vacuole |
| ATG16L1 | 0000421 | cellular component | autophagic vacuole membrane |
| ATG16L1 | 0034045 | cellular component | pre-autophagosomal structure membrane |
| ATG16L1 | 0005515 | molecular function | protein bining |
| BCAS1 | NA | NA | NA |
| C11orf36 | NA | NA | NA |
| C2orf16 | NA | NA | NA |
| CARS | 0006423 | biological process | cysteinyl-tRNA aminoacylation |
| CARS | 0005737 | cellular component | cytoplasm |
| CARS | 0005524 | molecular function | ATP binding |
| CARS | 0004817 | molecular function | cysteine-tRNA ligase activity |
| CASR | 0009653 | biological process | anatomical structure morphogenesis |
| CASR | 0070509 | biological process | calcium ion import |
| CASR | 0019722 | biological process | calcium-mediated signaling |
| CASR | 0006874 | biological process | cellular calcium ion homeostasis |
| CASR | 0007635 | biological process | chemosensory behavior |
| CASR | 0005513 | biological process | detection of calcium ion |
| CASR | 0007186 | biological process | G-coupled receptor signaling pathway |
| CASR | 0005887 | cellular component | integral to plasma membrane |
| CASR | 0005886 | cellular component | plasma membrane |
| CASR | 0004930 | molecular function | G-protein-coupled receptor activity |
| CASR | 0004435 | molecular function | phosphatidylinositol phospholipase C activity |
| CASR | 0005515 | molecular function | protein binding |
| CCDC121 | NA | NA | NA |
| CDKN1C | 0007050 | biological process | cell cycle arrest |
| CDKN1C | 0000080 | biological process | G1 phase of mitotic cell cycle |
| CDKN1C | 0008285 | biological process | negative regulation of cell proliferation |
| CDKN1C | 0050680 | biological process | negative regulation of epithelial cell proliferation |
| CDKN1C | 0033673 | biological process | negative regulation of kinase activity |
| CDKN1C | 0042326 | biological process | negative regulation of phosphorylation |
| CDKN1C | 0000122 | biological process | negative regulation of transcription from RNA polymerase II promoter |
| CDKN1C | 0045892 | biological process | negative regulation of transcription, DNA-dependent |
| CDKN1C | 0042551 | biological process | neuron maturation |
| CDKN1C | 0045893 | biological process | positive regulation of transcription, DNA-dependent |
| CDKN1C | 0030511 | biological process | positive regulation of transforming growth factor beta receptor signaling pathway |
| CDKN1C | 0005737 | cellular component | cytoplasm |
| CDKN1C | 0005634 | cellular component | nucleolus |
| CDKN1C | 0005634 | cellular component | nucleus |
| CDKN1C | 0004861 | molecular function | cyclin-dependent protein kinase inhibitor activity |
| CDKN1C | 0004861 | molecular function | cyclin-dependent protein kinase inhibitor activity |
| CDKN1C | 0005515 | molecular function | protein binding |
| CYP24A1 | 0001649 | biological process | osteoblast differentiation |
| CYP24A1 | 0055114 | biological process | oxidation-reduction process |
| CYP24A1 | 0033280 | biological process | response to vitamin D |
| CYP24A1 | 0044281 | biological process | small molecular metabolic process |
| CYP24A1 | 0008202 | biological process | steroid metabolic process |
| CYP24A1 | 0042369 | biological process | vitamin D catabolic process |
| CYP24A1 | 0042359 | biological process | vitamin D metabolic process |
| CYP24A1 | 0070561 | biological process | vitamin D receptor signaling pathway |
| CYP24A1 | 0006766 | biological process | vitamin metabolic process |
| CYP24A1 | 0006805 | biological process | xenobiotic metabolic process |
| CYP24A1 | 0005743 | cellular component | mitochondrial inner membrane |
| CYP24A1 | 0030342 | molecular function | 1-alpha, 25-dihydroxyvitamin D3 24-hydroxylases activity |
| CYP24A1 | 0008403 | molecular function | 25-hydroxycholecalciferol-24-hydroxylase activity |
| CYP24A1 | 0009055 | molecular function | electrion carrier activity |
| CYP24A1 | 0020037 | molecular function | heme binding |
| CYP24A1 | 0016491 | molecular function | oxidoreductase activity |
| DGKD | 0007596 | biological process | blood coagulation |
| DGKD | 0016049 | biological process | cell growth |
| DGKD | 0046339 | biological process | diacylglycerol metabolic process |
| DGKD | 0006897 | biological process | endocytosis |
| DGKD | 0007173 | biological process | epidermal growth factor receptor signaling pathway |
| DGKD | 0035556 | biological process | intracellular signal transduction |
| DGKD | 0007275 | biological process | multicellular organismal development |
| DGKD | 0030168 | biological process | platelet activation |
| DGKD | 0007205 | biological process | protein kinase C-activating G-protein coupled receptor signaling pathway |
| DGKD | 0005622 | cellular component | intracellular |
| DGKD | 0004143 | molecular function | diacylglycerol kinase activity |
| DGKD | 0046872 | molecular function | metal ion binding |
| DGKD | 0005543 | molecular function | phospholipid binding |
| DGKH | 0035556 | biological process | intracellular signal transduction |
| DGKH | 0007205 | biological process | protein kinase C-activating G-protein coupled receptor signaling pathway |
| DGKH | 0005737 | cellular component | cytoplasm |
| DGKH | 0031965 | cellular component | nuclear membrane |
| DGKH | 0005634 | cellular component | nucleus |
| DGKH | 0004143 | molecular function | diacylglycerol kinase activity |
| DGKH | 0005543 | molecular function | phospholipid binding |
| DNAJC5G | 0006457 | biolocial process | protein binding |
| DNAJC5G | 0031072 | molecular function | heat shock protein binding |
| DNAJC5G | 0051082 | molecular function | unfolded protein binding |
| [EIF2B4](#RANGE!_ENREF_21) | 0044237 | biological process | cellular metabolic process |
| [EIF2B4](#RANGE!_ENREF_21) | 0044267 | biological process | cellular protein metabolic process |
| [EIF2B4](#RANGE!_ENREF_21) | 0051716 | biological process | cellular response to stimulus |
| [EIF2B4](#RANGE!_ENREF_21) | 0010467 | biological process | gene expression |
| [EIF2B4](#RANGE!_ENREF_21) | 0042552 | biological process | myelinisation |
| [EIF2B4](#RANGE!_ENREF_21) | 0045947 | biological process | negative regulation of translational initiation |
| [EIF2B4](#RANGE!_ENREF_21) | 0014003 | biological process | oligodendrocyte development |
| [EIF2B4](#RANGE!_ENREF_21) | 0001541 | biological process | ovarian follicle development |
| [EIF2B4](#RANGE!_ENREF_21) | 0006417 | biological process | regulation of translation |
| [EIF2B4](#RANGE!_ENREF_21) | 0006446 | biological process | regulation of translational initiation |
| [EIF2B4](#RANGE!_ENREF_21) | 0009749 | biological process | response to glucose stimulus |
| [EIF2B4](#RANGE!_ENREF_21) | 0008408 | biological process | response to heat |
| [EIF2B4](#RANGE!_ENREF_21) | 0043434 | biological process | response to peptide hormone stimulus |
| [EIF2B4](#RANGE!_ENREF_21) | 0006412 | biological process | translation |
| [EIF2B4](#RANGE!_ENREF_21) | 0006413 | biological process | translation initiation |
| [EIF2B4](#RANGE!_ENREF_21) | 0005829 | cellular component | cytosol |
| [EIF2B4](#RANGE!_ENREF_21) | 0005829 | cellular component | cytosol |
| [EIF2B4](#RANGE!_ENREF_21) | 0005851 | cellular component | eukaryotic translation initiation factor 2B complex |
| [EIF2B4](#RANGE!_ENREF_21) | 0046523 | molecular function | 5-thioribose-1-phosphate isomerase activity |
| [EIF2B4](#RANGE!_ENREF_21) | 0005515 | molecular function | protein binding |
| [EIF2B4](#RANGE!_ENREF_21) | 0003743 | molecular function | translation initiation factor activity |
| [EIF2B4](#RANGE!_ENREF_21) | 0003743 | molecular function | translation initiation factor activity |
| [EIF2B4](#RANGE!_ENREF_21) | 00031369 | molecular function | translation initiation factor activity |
| FNDC4 | NA | NA | NA |
| FTHL3P | NA | NA | NA |
| GATA3 | 0005730 | cellular component | nucleolus |
| GATA3 | 0005634 | cellular component | nucleus |
| GCKR | 0005975 | biological process | carbohydrate metabolic process |
| GCKR | 0001678 | biological process | cellular glucose homeostasis |
| GCKR | 0010628 | biological process | positive regulation of gene expression |
| GCKR | 0033133 | biological process | positive regulation of glucokinase activity |
| GCKR | 0000060 | biological process | protein import into nucleus, translocation |
| GCKR | 0005634 | cellular component | nucleus |
| GCKR | 0030246 | molecular function | carbohydrate binding |
| [GPN1](#RANGE!_ENREF_30) | 0005737 | cellular component | cytoplam |
| [GPN1](#RANGE!_ENREF_30) | 0042331 | cellular component | Intracellular membrane-bounded organelle |
| [GPN1](#RANGE!_ENREF_30) | 0005524 | molecular function | ATP binding |
| [GPN1](#RANGE!_ENREF_30) | 0005525 | molecular function | GTP binding |
| [GPN1](#RANGE!_ENREF_30) | 0017111 | molecular function | nucleoside triphosphatase activity |
| [GPN1](#RANGE!_ENREF_30) | 0017111 | molecular function | nucleoside-triphophatase activity |
| [GPN1](#RANGE!_ENREF_30) | 0000166 | molecular function | Nucleotide binding |
| GTF3C2 | NA | NA | NA |
| [IFT172](#RANGE!_ENREF_18) | 0042384 | biological process | cilium assembly |
| [IFT172](#RANGE!_ENREF_18) | 0007368 | biological process | determination of left/right symmetry |
| [IFT172](#RANGE!_ENREF_18) | 0009953 | biological process | dorsal/ventral pattern formation |
| [IFT172](#RANGE!_ENREF_18) | 0001841 | biological process | neural tube formation |
| [IFT172](#RANGE!_ENREF_18) | 0016485 | biological process | protein processing |
| [IFT172](#RANGE!_ENREF_18) | 0007224 | biological process | smoothened signaling pathway |
| [IFT172](#RANGE!_ENREF_18) | 0005929 | cellular component | cilium |
| INPP5D | 0008340 | biological process | determination of adult lifespan |
| INPP5D | 0016064 | biological process | immunoglobulin mediated immune response |
| INPP5D | 0035556 | biological process | intracellular signal transduction |
| INPP5D | 0030889 | biological process | negative regulation of B cell proliferation |
| INPP5D | 0045779 | biological process | negative regulation of bone resorption |
| INPP5D | 0050777 | biological process | negative regulation of immune response |
| INPP5D | 0045409 | biological process | negative regulation of interleukin-6 biosynthetic process |
| INPP5D | 0045656 | biological process | negative regulation of monocyte differentiation |
| INPP5D | 0045659 | biological process | negative regulation of neutrophil differentiation |
| INPP5D | 0045671 | biological process | negative regulation of osteoclast differentiation |
| INPP5D | 0009968 | biological process | negative regulation of signal transduction |
| INPP5D | 0046854 | biological process | phosphatidylinositol phosphorylation |
| INPP5D | 0043065 | biological process | positive regulation of apoptotic process |
| INPP5D | 0045579 | biological process | positive regulation of B cell differenciation |
| INPP5D | 0045648 | biological process | positive regulation of erythrocyte differenciation |
| INPP5D | 0005884 | cellular component | actin filament |
| INPP5D | 0030863 | cellular component | cortical cystoskeleton |
| INPP5D | 0005829 | cellular component | cytosol |
| INPP5D | 0005886 | cellular component | plasma membrane |
| INPP5D | 0030487 | molecular function | inositol-4,5-bisphophate 5-phophatase activity |
| INPP5D | 0004445 | molecular function | inositol-polyphophate 5-phophatase activity |
| INPP5D | 0034594 | molecular function | phosphatidylinositol trisphophate phosphatase activity |
| INPP5D | 0051425 | molecular function | PTB domain binding |
| INPP5D | 0017124 | molecular function | SH3 domain binding |
| KCNQ1 | 0008015 | biological process | blood circulation |
| KCNQ1 | 00016458 | biological process | gene silencing |
| KCNQ1 | 0006936 | biological process | muscle contraction |
| KCNQ1 | 0046676 | biological process | negative regulation of insulin secrtion |
| KCNQ1 | 0071435 | biological process | potassium ion export |
| KCNQ1 | 0006349 | biological process | regulation of gene expression by genetic imprinting |
| KCNQ1 | 0008016 | biological process | regulation of heart contraction |
| KCNQ1 | 0060306 | biological process | regulation of membrane repolarization |
| KCNQ1 | 0042221 | biological process | response to chemical stimulus |
| KCNQ1 | 0007605 | biological process | sensory perception of sound |
| KCNQ1 | 0007268 | biological process | synaptic transmission |
| KCNQ1 | 0016323 | cellular component | basolateral plasma membrane |
| KCNQ1 | 0005769 | cellular component | early endosome |
| KCNQ1 | 0005770 | cellular component | late endosome |
| KCNQ1 | 0005770 | cellular component | late endosome |
| KCNQ1 | 0005764 | cellular component | lysosome |
| KCNQ1 | 0005886 | cellular component | plasma membrane |
| KCNQ1 | 0042383 | cellular component | sarcolemma |
| KCNQ1 | 0008076 | cellular component | voltage-gated potassium channel complex |
| KCNQ1 | 0042589 | cellular component | zymogen granule membrane |
| KCNQ1 | 0005516 | molecular function | calmodulin binding |
| KCNQ1 | 0005251 | molecular function | delayed rectified potassium channel activity |
| KCNQ1 | 0005249 | molecular function | gated potassium channel activity |
| KCNQ1 | 0015271 | molecular function | outward rectifier potassium channel activity |
| KCNQ1 | 0005249 | molecular function | voltage-gated potassium channel activity |
| KCNQ1OT1 | NA | NA | NA |
| KIAA0564 | 0005576 | cellular component | extracellular region |
| KIAA0564 | 0005739 | cellular component | mitochondrion |
| KIAA0564 | 0005524 | molecular function | ATP binding |
| KIAA0564 | 0016887 | molecular function | ATPase activity |
| KRTCAP3 | NA | NA | NA |
| MPV17 | 0034614 | biological process | cellular response to reactive oxygen species |
| MPV17 | 0032836 | biological process | glomerular membrane development |
| MPV17 | 0042592 | biological process | homeostatic process |
| MPV17 | 0048839 | biological process | inner ear development |
| MPV17 | 0000002 | biological process | mitochondrial genome maintenance |
| MPV17 | 0072593 | biological process | reactive oxygen species metabolis process |
| MPV17 | 2000377 | biological process | regulation of reactive oxygen species metabolic process |
| MPV17 | 0016021 | cellular component | integral to membrane |
| MPV17 | 0016021 | cellular component | integral to plasma membrane |
| MPV17 | 0005743 | cellular component | mitochondrial inner membrane |
| MPV17 | 0005739 | cellular component | mitochondrion |
| MPV17 | 0005777 | cellular component | peroxisome |
| MPV17 | 0005102 | molecular function | receptor binding |
| MRGPRE | 0016021 | cellular component | integral to membrane |
| MRGPRE | 0005886 | cellular component | Plasma membrane |
| MRGPRE | 0004930 | molecular function | G-protein coupled receptor activity |
| MRGPRG | NA | NA | NA |
| NAP1L4 | 0006334 | biological process | nucleosome assembly |
| NAP1L4 | 0005678 | cellular component | chromotin assembly complex |
| NAP1L4 | 0005737 | cellular component | cytoplasm |
| NAP1L4 | 0005634 | cellular component | nucleus |
| NAP1L4 | 0005515 | molecular function | protein binding |
| NAP1L4 | 0051082 | molecular function | unfolded protein binding |
| [NRBP1](#RANGE!_ENREF_19) | 0006888 | biological process | ER to Golgi vesicle-mediated transport |
| [NRBP1](#RANGE!_ENREF_19) | 0010467 | biological process | gene expression |
| [NRBP1](#RANGE!_ENREF_19) | 0006367 | biological process | transcription initiation from RNA polymerase II promoter |
| [NRBP1](#RANGE!_ENREF_19) | 0005938 | cellular component | cell cortex |
| [NRBP1](#RANGE!_ENREF_19) | 0012505 | cellular component | endomembrane system |
| [NRBP1](#RANGE!_ENREF_19) | 0016020 | cellular component | membrane |
| [NRBP1](#RANGE!_ENREF_19) | 005654 | cellular component | nucleoplasm |
| [NRBP1](#RANGE!_ENREF_19) | 0048471 | cellular component | perinuclear region of cytoplasm |
| [NRBP1](#RANGE!_ENREF_19) | 0048471 | cellular component | perinuclear region of cytoplasm |
| [NRBP1](#RANGE!_ENREF_19) | 0005524 | molecular function | ATP binding |
| [NRBP1](#RANGE!_ENREF_19) | 0005524 | molecular function | ATP binding |
| [NRBP1](#RANGE!_ENREF_19) | 005515 | molecular function | protein binding |
| [NRBP1](#RANGE!_ENREF_19) | 0042803 | molecular function | protein homodimerization activity |
| [NRBP1](#RANGE!_ENREF_19) | 0004672 | molecular function | protein kinase activity |
| OSBPL5 | 0008203 | biological process | cholesterol metabolic process |
| OSBPL5 | 0030301 | biological process | cholesterol transport |
| OSBPL5 | 0006893 | biological process | Golgi to plama membrane transport |
| OSBPL5 | 0006869 | biological process | lipid transport |
| OSBPL5 | 0005829 | cellular component | cytosol |
| OSBPL5 | 0005729 | cellular component | endoplasmic reticulum membrane |
| OSBPL5 | 0016021 | cellular component | integral to membrane |
| OSBPL5 | 0008142 | molecular function | oxysterol binding |
| OSBPL5 | 0005543 | molecular function | phopholipid binding |
| OSBPL5 | 0005543 | molecular function | phospholipid binding |
| PFDN4 | 0051084 | biological process | ‘de novo’ posttranslational protein folding |
| PFDN4 | 0044267 | biological process | cellular protein metabolic process |
| PFDN4 | 0006457 | biological process | protein folding |
| PFDN4 | 0005829 | cellular component | cytosol |
| PFDN4 | 0005739 | cellular component | mitochondrion |
| PFDN4 | 0016272 | cellular component | prefoldin complex |
| PFDN4 | 0051087 | molecular function | chaperone binding |
| PFDN4 | 0005515 | molecular function | protein binding |
| PFDN4 | 0051082 | molecular function | unfolded protein binding |
| PHLDA2 | NA | NA | NA |
| PPM1G | NA | NA | NA |
| SAG | 0007166 | biological process | cell surface receptor signaling pathway |
| SAG | 0016056 | biological process | rhodopsin mediated signaling pathway |
| SAG | 0007165 | biological process | signal transduction |
| SAG | 0007601 | biological process | visual perception |
| SAG | 0002046 | molecular function | opsin binding |
| SAG | 0051219 | molecular function | phosphoprotein binding |
| SAG | 0004864 | molecular function | protein phosphatase inhibitor activity |
| SCARNA5 | NA | NA | NA |
| SCARNA6 | NA | NA | NA |
| SLC22A18 | 0006855 | biological process | drug transmembrane transport |
| SLC22A18 | 0015893 | biological process | drug transport |
| SLC22A18 | 0007588 | biological process | excretion |
| SLC22A18 | 0015695 | biological process | organic cation transport |
| SLC22A18 | 0055085 | biological process | transmembrane transport |
| SLC22A18 | 0016324 | cellular component | apical plasma membrane |
| SLC22A18 | 0016021 | cellular component | integral to membrane |
| SLC22A18 | 0005635 | cellular component | nuclear envelop |
| SLC22A18 | 0005886 | cellular component | plasma membrane |
| SLC22A18 | 0015238 | molecular function | drug transmembrane transporter activity |
| SLC22A18 | 0015293 | molecular function | symporter activity |
| SLC22A18 | 0005215 | molecular function | transporter activity |
| SLC22A18 | 0031625 | molecular function | ubiquitin protein ligase binding |
| SLC22A18AS | NA | NA | NA |
| SNORA54 | NA | NA | NA |
| [SNX17](#RANGE!_ENREF_20) | 0007154 | biological process | cell communication |
| [SNX17](#RANGE!_ENREF_20) | 006707 | biological process | cholesterol catabolic process |
| [SNX17](#RANGE!_ENREF_20) | 0016197 | biological process | endosomal transport |
| [SNX17](#RANGE!_ENREF_20) | 0006898 | biological process | intracellular protein transport |
| [SNX17](#RANGE!_ENREF_20) | 0006898 | biological process | receptor–mediated endocytosis |
| [SNX17](#RANGE!_ENREF_20) | 0030100 | biological process | regulation of endocytosis |
| [SNX17](#RANGE!_ENREF_20) | 0007165 | biological process | signal transduction |
| [SNX17](#RANGE!_ENREF_20) | 0005737 | cellular component | cytoplasm |
| [SNX17](#RANGE!_ENREF_20) | 0016023 | cellular component | cytoplasmic membrane-bounded vesicle |
| [SNX17](#RANGE!_ENREF_20) | 0030659 | cellular component | cytoplasmic vesicle membrane |
| [SNX17](#RANGE!_ENREF_20) | 0005829 | cellular component | cytosol |
| [SNX17](#RANGE!_ENREF_20) | 0005769 | cellular component | early endosome |
| [SNX17](#RANGE!_ENREF_20) | 0005768 | cellular component | endosome |
| [SNX17](#RANGE!_ENREF_20) | 0005794 | cellular component | Golgi apparatus |
| [SNX17](#RANGE!_ENREF_20) | 0043231 | cellular component | intracellular membrane bound organelle |
| [SNX17](#RANGE!_ENREF_20) | 0016020 | cellular component | membrane |
| [SNX17](#RANGE!_ENREF_20) | 0050750 | molecular function | low-density lipoprotein particle receptor binding |
| [SNX17](#RANGE!_ENREF_20) | 0035091 | molecular function | phosphatidylinositol binding |
| [SNX17](#RANGE!_ENREF_20) | 0035091 | molecular function | phosphatidylinositor binding |
| [SNX17](#RANGE!_ENREF_20) | 0005515 | molecular function | protein binding |
| [SNX17](#RANGE!_ENREF_20) | 0008022 | molecular function | protein C-terminus binding |
| [SNX17](#RANGE!_ENREF_20) | 0005102 | molecular function | receptor binding |
| SUPT7L | NA | NA | NA |
| [TRIM54](#RANGE!_ENREF_27) | NA | NA | NA |
| UCN | 0042756 | biological process | drinking behavior |
| UCN | 0007565 | biological process | female pregnancy |
| UCN | 0007186 | biological process | G-protein coupled receptor signaling pathway |
| UCN | 0006954 | biological process | inflammatory response |
| UCN | 0007611 | biological process | learning or memory |
| UCN | 00045776 | biological process | negative regulation of blood pressure |
| UCN | 0031175 | biological process | neuron projection development |
| UCN | 0007218 | biological process | neuropeptide signaling pathway |
| UCN | 0030157 | biological process | pancreatic juice secretion |
| UCN | 0030307 | biological process | positive regulation of cell growth |
| UCN | 0032967 | biological process | positive regulation of collagen biosynthetic process |
| UCN | 0032755 | biological process | positive regulation of interleukin-6 production |
| UCN | 0045727 | biological process | positive regulation of translation |
| UCN | 0051966 | biological process | regulation of synaptic transmission glutamatergic |
| UCN | 0051384 | biological process | response to estradiol stimulus |
| UCN | 0051384 | biological process | response to glucocorticoid stimulus |
| UCN | 0006979 | biological process | response to oxidative stress |
| UCN | 0043679 | cellular component | axon terminus |
| UCN | 0030425 | cellular component | dendrite |
| UCN | 0005576 | cellular component | extracellular region |
| UCN | 0043025 | cellular component | neuronal cell body |
| UCN | 0005184 | molecular function | neuropeptide hormone activity |
| UCN | 0005184 | molecular function | protein binding |
| USP40 | 0006511 | biological process | ubiquitin-dependent protein catabolic process |
| USP40 | 0008234 | molecular function | cysteine-type peptidase activity |
| USP40 | 0004221 | molecular function | ubiquitin thiolesterase activity |
| ZNF512 | 0006355 | biological process | regulation of transcription, DNA dependent |
| ZNF512 | 0006351 | biological process | transcription, DNA dependent |
| ZNF512 | 0005622 | cellular component | intracellular |
| ZNF512 | 0005634 | cellular component | nucleus |
| ZNF512 | 0003677 | molecular function | DNA binding |
| ZNF512 | 0008270 | molecular function | zinc ion binding |
| [ZNF513](#RANGE!_ENREF_22) | 0006355 | biological process | regulation of transcription, DNA dependent |
| [ZNF513](#RANGE!_ENREF_22) | 0050896 | biological process | response to stimulus |
| [ZNF513](#RANGE!_ENREF_22) | 0060041 | biological process | retina development in camera-type eye |
| [ZNF513](#RANGE!_ENREF_22) | 0006351 | biological process | transcription, DNA dependent |
| [ZNF513](#RANGE!_ENREF_22) | 0005622 | cellular component | intracellular |
| [ZNF513](#RANGE!_ENREF_22) | 0005634 | cellular component | nucleus |
| [ZNF513](#RANGE!_ENREF_22) | 0003677 | molecular function | DNA binding |
| [ZNF513](#RANGE!_ENREF_22) | 0044212 | molecular function | transcription regulatory region DNA binding |
| [ZNF513](#RANGE!_ENREF_22) | 0008270 | molecular function | zinc ion binding |
