# Supplementary material for: Meta-Analysis of Genome-Wide Association Studies Identifies Six New Loci for Serum Calcium Concentrations
Source: PLoS Genet. 2013 Sep 19;9(9):e1003796. doi: 10.1371/journal.pgen.1003796 (PMC3778004; doi:10.1371/journal.pgen.1003796)
Supplement: Table S9 — OMIM disorders associated with the genes located within the replicated loci. This table includes all Mendelian disorders or other types of genetic disorders included in the OMIM database described for each gene located within ±250 kb of any of the six new loci and for CASR. (DOCX) [file pgen.1003796.s017.docx]

## Table S9: OMIM disorders associated with the genes located within the replicated loci

| **Gene** | **OMIM number** | **OMIM disorder** | **Mechanism** |
| --- | --- | --- | --- |
| ATG16L1 | #611081 | Inflammatory bowel disease 10 | non-synonymous polymorphism associated with reduced protein function |
| BCAS1 | NONE |  |  |
| C11orf36  (MRGPRG antisense RNA) | NONE |  |  |
| C2orf16 | NONE |  |  |
| CARS | NONE |  |  |
| CASR | #145980 | Hypocalciuric hypercalcemia, familial, type I | heterozygous loss-of-function mutations |
| CASR | #239200 | Neonatal severe hyperparathyroidism | loss-of-function |
| CCDC121 | NONE |  |  |
| CDKN1C | **#**130650 | Beckwith-Wiedemann syndrome | imprinting |
| CYP24A1 | #143880 | Hypercalcemia, idiopathic, of infancy | homozygous or compound heterozygous mutation |
| DGKD | NONE |  |  |
| DGKH | NONE |  |  |
| DNAJC5G | NONE |  |  |
| [EIF2B4](#RANGE!_ENREF_21) | #603896 | Leukoencephaly with vanishing white matter ; Ovarioleukodystrophy | autosomal recessive transmission. |
| FNDC4 | NONE |  |  |
| FTHL3P | NONE |  |  |
| GATA3 | #146255 | Hypoparathyroidism, sensorineural deafness, and renal dysplasia | haploinsufficiency |
| GCKR | #61463 | fasting plasma glucose level quantitative trait locus |  |
| [GPN1](#RANGE!_ENREF_30) | NONE |  |  |
| GTF3C2 | NONE |  |  |
| [IFT172](#RANGE!_ENREF_18) | NONE |  |  |
| INPP5D | NONE |  |  |
| KCNQ1 | #607554 | Atrial fibrillation, familial, 3 | missense mutation |
| KCNQ1 | #220400 | Jervell and Lange-Nielsen syndrome | homozygous frameshift mutation or deletion |
| KCNQ1 | #192500 | Long QT syndrome-1 | various mechanisms described including missense mutation |
| KCNQ1 | #609621 | Short QT syndrome-2 | missense mutation |
| KCNQ1OT1 | **#**130650 | Beckwith-Wiedemann syndrome | imprinting |
| KIAA0564 | NONE |  |  |
| KRTCAP3 | NONE |  |  |
| MPV17 | #256810 | Mitochondrial DNA depletion syndrome 6 (hepatocerebral type) |  |
| MRGPRE | NONE |  |  |
| MRGPRG | NONE |  |  |
| NAP1L4 | NONE |  | located 100 kb centromeric to the proximal Beckwith-Wiedemann breakpoint cluster region |
| [NRBP1](#RANGE!_ENREF_19) | NONE |  |  |
| OSBPL5 | NONE |  |  |
| PFDN4 | NONE |  |  |
| PHLDA2 | NONE |  | near Beckwith-Wiedemann syndrome region |
| PPM1G | NONE |  |  |
| SAG | #258100 | Oguchi disease-1 | homozygous deletion resulting in functional null alleles |
| SAG | #613758 | Retinitis pigmentosa 47 | deletion |
| SCARNA5 | NONE |  |  |
| SCARNA6 | NONE |  |  |
| [SLC4A1AP](#RANGE!_ENREF_31) | NONE |  |  |
| [SNX17](#RANGE!_ENREF_20) | NONE |  |  |
| SUPT7L | NONE |  |  |
| [TRIM54](#RANGE!_ENREF_27) | NONE |  |  |
| UCN | NONE |  |  |
| ZNF512 | NONE |  |  |
| [ZNF513](#RANGE!_ENREF_22) | #61367 | Retinitis pigmentosa 58 | homozygous missense mutation |
