# Supplementary material for: Meta-Analysis of Genome-Wide Association Studies Identifies Six New Loci for Serum Calcium Concentrations
Source: PLoS Genet. 2013 Sep 19;9(9):e1003796. doi: 10.1371/journal.pgen.1003796 (PMC3778004; doi:10.1371/journal.pgen.1003796)
Supplement: Table S10 — Association of replicated serum calcium loci in other ethnic groups. Chr, chromosome. Position, position on build 36. A1, allele 1 (effect allele). A2, allele 2. Freq A1, frequency of allele 1. Effect A1, regression coefficient for the A1 allele. SE, standard error. NA, not available. (DOCX) [file pgen.1003796.s018.docx]

## Table S10: Association of replicated serum calcium loci in other ethnic groups

|  |  |  |  |  |  | **Indians Asians** | | | | | **Japanese** | | | | |
| --- | --- | --- | --- | --- | --- | --- | --- | --- | --- | --- | --- | --- | --- | --- | --- |
| **Markers*** | **chr** | **Position** | **Nearby Gene** | **A1** | **A2** | **N** | **Freq A1** | **Effect A1** | **SE** | **P**  **value** | **N** | **Freq A1** | **Effect A1** | **SE** | **P value** |
| **Known locus** |  |  |  |  |  |  |  |  |  |  |  |  |  |  |  |
| rs1801725 | 3 | 123486447 | *CASR* | t | g | 8318 | 0.20 | 0.083 | 0.007 | 1.4E-31 | 4431 | 0.01 | 0.142 | 0.044 | 0.001 |
| **Novel loci** |  |  |  |  |  |  |  |  |  |  |  |  |  |  |  |
| rs1550532 | 2 | 233929587 | *DGKD* | c | g | 8318 | 0.16 | 0.024 | 0.008 | 0.002 | 4431 | 0.28 | 0.013 | 0.012 | 0.26 |
| rs780094 | 2 | 27594741 | *GCKR* | t | c | 8318 | 0.26 | 0.002 | 0.006 | 0.75 | 4431 | 0.44 | -0.004 | 0.011 | 0.71 |
| rs10491003 | 10 | 9368657 | *GATA3* | t | c | 8318 | 0.08 | 0.027 | 0.011 | 0.009 | 4431 | 0.01 | 0.008 | 0.070 | 0.54 |
| rs7481584 | 11 | 2985665 | *CARS* | a | g | 8318 | 0.36 | -0.011 | 0.006 | 0.06 | 4431 | 0.35 | 0.001 | 0.011 | 0.54 |
| rs7336933 | 13 | 41457076 | *DGKH; KIAA0564* | a | g | 8318 | 0.04 | 0.013 | 0.014 | 0.38 | 4431 | NA | NA | NA | NA |
| rs1570669 | 20 | 52207834 | *CYP24A1* | a | g | 8318 | 0.37 | -0.003 | 0.006 | 0.58 | 4431 | 0.42 | 0.014 | 0.011 | 0.19 |
