# Supplementary material for: Meta-Analysis of Genome-Wide Association Studies Identifies Six New Loci for Serum Calcium Concentrations
Source: PLoS Genet. 2013 Sep 19;9(9):e1003796. doi: 10.1371/journal.pgen.1003796 (PMC3778004; doi:10.1371/journal.pgen.1003796)
Supplement: Table S11 — Plasma and Urine electrolytes values by calcium diet in mice. Data are means ± SEM of values obtained from 3 to 5 mice. *: P value≤0.05 compared to normal or high calcium diet. (DOCX) [file pgen.1003796.s019.docx]

## Table S11: Plasma and Urine electrolytes values by calcium diet in mice.

|  | **Low Ca^2+^ diet** | **Normal Ca^2+^ diet** | **High Ca^2+^ diet** |
| --- | --- | --- | --- |
| **Plasma** |  |  |  |
| Na^+^, mM | 149.6 ±0.9 | 150.8 ±0.5 | 149.2 ±0.6 |
| Ca^2+^, nM | 2.44 ±0.04 | 2.39 ±0.02 | *2.43* ±0.03 |
| PO_4_^-^ nM | 2.22 ±0.16 | 2.12 ±0.08 | *1.99* ±0.09 |
| **Urine** |  |  |  |
| Na^+^/creatinine | 54.78 ±4.16 | 46.66 ±11.38 | *64.68* ±14.68 |
| Ca^2+^/creatinine | 0.33 ±0.05 | 0.29 ±0.02 | *0.43* ±0.12 |
| PO_4_^-^/creatinine | 29.12 ±3.90* | 10.63 ±3.77 | *11.22* ± 2.28 |
