# Supplementary material for: Meta-Analysis of Genome-Wide Association Studies Identifies Six New Loci for Serum Calcium Concentrations
Source: PLoS Genet. 2013 Sep 19;9(9):e1003796. doi: 10.1371/journal.pgen.1003796 (PMC3778004; doi:10.1371/journal.pgen.1003796)
Supplement: Table S12 — Study information. (DOCX) [file pgen.1003796.s020.docx]

## Table S12: Study information

| **Study Name** | **Study Design** | **Genotyped sample size** | **Study exclusions or disease enrichment** | **Exclusions** | **Calcium Measurement + QC** | **Ref** |
| --- | --- | --- | --- | --- | --- | --- |
| **Discovery cohorts** |  |  |  |  |  |  |
| Age Gene/Environment Susceptibility Reykjavik Study (AGES) | Population based | 3664 | none | Sample exclusion criteria included sample failure, genotype mismatch with reference panel, and sex mismatch, resulting in clean genotype data on 3,219 individuals. | Serum calcium was measured using a colorimetric assay on a Hitachi 912 using a Roche Diagnostics assay . | [^1^](#_ENREF_1) |
| ARIC | Prospective, population-based(1) | 9713 of European ancestry | none | Of the 9713 genotyped individuals of European ancestry, we excluded 658 individuals based on discrepancies with previous genotypes, disagreement between reported and genotypic sex, one randomly selected member of a pair of first-degree relatives, or outlier based on measures of average DST or more than 8 SD away on any of the first 10 principal components. | Total serum calcium was measured at ARIC visit 1 (1987-89) using a colorimetric method on a DACOS analyzer (http://www.cscc.unc.edu/aric/visit/Clinical_Chemistry_Determinations.1_10.pdf). | [^2^](#_ENREF_2) |
| BLSA | Prospective population based study | 1230 | none | Non-European ancestry based on Eigenstrat (N=368), call rate <98.5 (N=5), sex misspecification (N=9), Missing calcium data (N=129) | Serum calcium was measured using a colorimetric assay. | [^3^](#_ENREF_3) |
| Cohorte Lausannoise (CoLaus) | Population-based cross-sectional study | 5435 | none | Individuals with genotyping efficiency <90% were removed. Some "measured" SNPs with missing values for some individuals were imputed. As a result, the rSqHat < 1. | For each CoLaus participant a venous blood sample was collected under fasting conditions. Total serum calcium was measured by O-cresolphtalein (2.1% –1.5% maximum inter and intra-batch CVs); albumin was measured by bromocresol green (2.5% – 0.4%). | [^4^](#_ENREF_4) |
| CROATIA-Vis | Family-based, cross-sectional study in an isolated population | 924 | none | Exclusions: sample call rate<95% | Serum calcium was measured using a colorimetric assay. | [^5^](#_ENREF_5) |
| CROATIA-Korcula | Family-based, cross-sectional study in an isolated population | 899 | none | Exclusions: sample call rate<97% | Serum calcium was measured using a colorimetric assay. | [^6^](#_ENREF_6) |
| CROATIA-Split | Population-based, cross-sectional study | 499 | none | Exclusions: sample call rate<97% | Serum calcium was measured using a colorimetric assay. | [^7^](#_ENREF_7) |
| Framingham Heart Study (FHS) | Prospective family-based | 9300 | none | Of the 9,274 participants who underwent genotyping, we made the following exclusions: sample call rate <97% (n=666), genotype heterozygosity > 5 standard deviations, and ambiguous family data (n=127). This resulted in a total of 8,481 genotyped individuals. | Serum calcium was measured using a colorimetric assay. | [^8-10^](#_ENREF_8) |
| HABC | prospective cohort study | 1663 | none | none | Serum calcium was measured using a colorimetric assay. | [^11^](#_ENREF_11) |
| InCHIANTI | Prospective population based study | 1231 | none | Ambiguous family data (N=4), call rate < 98.5% (N=12), sex misspecification (N=1), heterozygosity>0.3 (N=4), Missing calcium data (N=6) | Serum calcium was measured using a colorimetric assay. | [^12^](#_ENREF_12) |
| Lothian Birth Cohort 1936 (LBC1936) | Population based birth cohort | 1005 | none | Individuals with a disagreement between genetic and reported gender were removed (n = 12). Relatedness between subjects was investigated and, for any related pair of individuals, one was removed [PI_HAT (proportion of IBD) > 0.25, n = 8). Samples with a call rate ≤ 0.95 (n = 16), and those showing evidence of non-European descent by multidimensional scaling, were also removed (n = 1). | Serum calcium was measured using a colorimetric assay. | [^13^](#_ENREF_13)^,^[^14^](#_ENREF_14) |
| London Life Sciences Population (LOLIPOP) study | | |  |  |  |  |
| LOLIPOP EW A | Population based prospective cohort study | 878 | none | excluded samples of duplicates, contaminated samples, call rate <%95, and the same samples apeared in EW610 | Serum calcium was measured using standard approach | [^15^](#_ENREF_15) |
| LOLIPOP EW P | Population based prospective cohort study | 1005 | none | excluded samples of duplicates, contaminated samples, call rate <%95, and the same samples apeared in EW610 | Serum calcium was measured using standard approach | [^16^](#_ENREF_16) |
| LOLIPOP EW610 | Population based prospective cohort study | 945 | none | excluded samples of duplicates, call rate <%95, relatedness, gender discrepancy, ethnic outliers, and imcomplete clinical data | Serum calcium was measured using standard approach | [^17^](#_ENREF_17)^,^[^18^](#_ENREF_18) |
| Ogliastra Genetic Park - Talana Study | Population-based study with pedigree information | 860 | none |  | Calcium levels were determined with an automated Targa BT-3000 Chemistry Analyser ( ml/dL) | [^19-21^](#_ENREF_19) |
| ORCADES | Family-based, cross-sectional study in an isolated population | 889 | none | Exclusions: sample call rate<97% | Serum calcium was measured using a colorimetric assay. | [^22^](#_ENREF_22) |
| SHIP | Prospective population-based study | 4081 | none | Excluded arrays with CallRate < 92%, duplicate samples (by estimated IBD) and individuals with reported/genotyped gender mismatch | Non-fasting blood samples were drawn from the cubital vein in the supine position between 7.00 a.m. and 7.00 p.m. The measurement of total calcium concentration was performed immediately after blood withdrawal. Samples were analysed on the Hitachi 911 by o-cresolphthalein complexone colorimetry (Boehringer Mannheim, Germany). The internal quality controls were analyzed daily. During the course of the study the inter as well as the intra-assay coefficient of variation was <5%. In addition, the laboratory takes part quarterly in the official national German external proficiency testing programs and fulfilled the requirements. | [^23^](#_ENREF_23) |
| The Rotterdam Study (RS) | Prospective population based study | 5974 | NA | Any samples with a call rate below 97.5%, excess autosomal heterozygosity >0.336 (~FDR <0.1%), mismatch between called and phenotypic gender, or if there were outliers identified by the IBS clustering analysis (see below) with >3 standard deviations from population mean or IBS probabilities >97% were excluded from the analysis | Serum calcium was measured at baseline visit in the Rotterdam Study with a colorimetric detection assay using the Hitachi 917 (Roche, Mannheim, Germany). | [^24^](#_ENREF_24)^,^[^25^](#_ENREF_25) |
| The Cardiovascular Health Study (CHS) | Prospective, population-based | 3,329 CHS Caucasian participants | A total of 1908 persons were excluded from the GWAS study sample due to the presence at study baseline of coronary heart disease, congestive heart failure, peripheral vascular disease, valvular heart disease, stroke or transient ischemic attack or lack of available DNA. | The present report is based upon genotyping results from 3,329 CHS Caucasian participants, who were free of clinical cardiovascular disease at baseline, consented to genetic testing, and had DNA available for genotyping. Genotypes were called using the Illumina BeadStudio software. Genotyping was successful in 3,291 persons. | Serum calcium has been measured in unit mg/dL | [^26^](#_ENREF_26) |
| **Replication cohorts** |  |  |  |  |  |  |
| Bus Santé study | Cross-sectional population-based study | 5622 | none | Of the 5,622 participants who underwent genotyping, genotyping was unsuccessfull for 2.7% (N=151). This resulted in a total of 5,471 genotyped individuals. Analyses were restriced to Caucasians. Caucasian was defined as self-reported citizenship corresponding to South/North America, Europe, and Australia regions | Serum calcium was measured using a colorimetric assay using Arsenazo-III reactive (Architect CI4100®, Abbott). Coefficient of variation (CV) = 5.4% | [^27-29^](#_ENREF_27) |
| INGI-Carlantino-Project | Isolated population | 679 | none | we made the following exclusions: sample call rate <97% | Serum calcium was measured using a colorimetric assay. |  |
| INGI-FVG-Project | Isolated population | 1471 | none | we made the following exclusions: sample call rate <97% | Serum calcium was measured using a colorimetric assay. |  |
| INGI-CILENTO | Cross-sectional population based | 1147 | none | none | Serum calcium was measured using a colorimetric assay. | [^30-41^](#_ENREF_30) |
| KORA F3 Study (Cooperative Health Research in the Region of Augsburg) | Population-based | 1643 | none | 3, because of no available information on serum calcium. This resulted in a total of 1640 individuals | Serum calcium was measured using a colorimetric assay. | [^42^](#_ENREF_42)^,^[^43^](#_ENREF_43) |
| KORA F4 Study | Population-based | 1814 | none | 5, because of no available  information on serum calcium. This resulted in a total of 1809 individuals | Serum calcium was measured using a colorimetric assay. | [^42^](#_ENREF_42)^,^[^43^](#_ENREF_43) |
| LURIC Study | Case-control | 3032 |  | sample call rate <95%, gender ambiguity, relatedness. This resulted in a total of 2927 genotyped individuals. | o-Kresolphthalein-complexon, CA/Hitachi 717, Roche, Germany | [^44^](#_ENREF_44) |
| PIVUS | Prospective cohort | 958 | none | Sample call rate <95%, genotype heterozygosity > +-3 standard deviations, gender discordance, and duplicates. | Reference method at Uppsala University Hospital | [^45^](#_ENREF_45) |
| SHIP-Trend | Prospective population-based study | 986 | none | Excluded arrays with CallRate < 94%, duplicate samples (by estimated IBD) and individuals with reported/genotyped gender mismatch | Blood samples in SHIP-Trend were taken, while subjects were on random salt diet and under regular medication, from fasting subjects in supine position. Serum calcium has been measured by photometric rocedure of bichromatic endpoint measurement on an Dimension Vista system (Dade Behring, Eschborn, Germany). The analytical measurement range was: 1.25-3.75 mmol/L. During the course of the study the inter-assay coefficient of variation was < 5%. | [^23^](#_ENREF_23) |
| TwinsUK | Twin Study | 3965 | none | Of the 5,654 participants who underwent genotyping, we made the following exclusions: >3 standard deviations and missing informations. This resulted in a total of 3965 genotyped individuals. | Assays for serum calcium was performed using standard laboratory procedures. The test was performed on a 950 Vitros analyser (Ortho-Clinical Diagnostics; Johnson and Johnson, Rochester, NY, U.S.A.). | [^46^](#_ENREF_46)^,^[^47^](#_ENREF_47) |
| The BRItish Genetics of HyperTension (BRIGHT) study | Hypertensive cases from the BRIGHT study resource. | 2000 | BMI>35  diabetes, secondary hypertension or a co-existing illness. | Of 2000 cases typed, we excluded individuals if they had >3% missing data or evidence of non-European ancestry under eigenstrat analysis, n=277. | Serum calcium measures were performed on non-fasting samples by the Clinical Biochemistry Unit at the University of Glasgow | [^48^](#_ENREF_48) |
| **Other studies** |  |  |  |  |  |  |
| Biobank Japan | DNA bank for multiple diseases | 4944 | none | sample call rate <0.99, >2 standard deviations, | Serum calcium was measured with Arsenazo III method. | [^49-51^](#_ENREF_49) |
| LOLIPOP IA P | Population based prospective cohort study | 1006 | none | excluded samples of duplicates, contaminated samples, call rate <%95, and the same samples apeared in IA610 | Serum calcium was measured using standard approach | [^16^](#_ENREF_16) |
| LOLIPOP IA317 | Population based prospective cohort study | 2694 | none | excluded samples of duplicates, call rate <%97, relatedness, gender discrepancy, ethnic outliers, and imcomplete clinical data, and the same samples apeared in IA610 | Serum calcium was measured using standard approach | [^52^](#_ENREF_52) |
| LOLIPOP IA610 | Population based prospective cohort study | 7032 | CHD case-control study | excluded samples of duplicates, call rate <%95, relatedness, gender discrepancy, ethnic outliers, and imcomplete clinical data | Serum calcium was measured using standard approach | [^53^](#_ENREF_53)^,^[^54^](#_ENREF_54) |

References

1. Harris, T.B. et al. Age, Gene/Environment Susceptibility-Reykjavik Study: multidisciplinary applied phenomics. *American journal of epidemiology* **165**, 1076-87 (2007).

2. The Atherosclerosis Risk in Communities (ARIC) Study: design and objectives. The ARIC investigators. *American journal of epidemiology* **129**, 687-702 (1989).

3. Shock, N. Normal Human Aging: The Baltimore Study of Aging. . (1984).

4. Firmann, M. et al. The CoLaus study: a population-based study to investigate the epidemiology and genetic determinants of cardiovascular risk factors and metabolic syndrome. *BMC cardiovascular disorders* **8**, 6 (2008).

5. Vitart, V. et al. SLC2A9 is a newly identified urate transporter influencing serum urate concentration, urate excretion and gout. *Nature genetics* **40**, 437-42 (2008).

6. Zemunik, T. et al. Genome-wide association study of biochemical traits in Korcula Island, Croatia. *Croatian medical journal* **50**, 23-33 (2009).

7. Rudan, I. et al. "10001 Dalmatians:" Croatia launches its national biobank. *Croatian medical journal* **50**, 4-6 (2009).

8. Feinleib, M., Kannel, W.B., Garrison, R.J., McNamara, P.M. & Castelli, W.P. The Framingham Offspring Study. Design and preliminary data. *Preventive medicine* **4**, 518-25 (1975).

9. Kannel, W.B., Feinleib, M., McNamara, P.M., Garrison, R.J. & Castelli, W.P. An investigation of coronary heart disease in families. The Framingham offspring study. *American journal of epidemiology* **110**, 281-90 (1979).

10. Splansky, G.L. et al. The Third Generation Cohort of the National Heart, Lung, and Blood Institute's Framingham Heart Study: design, recruitment, and initial examination. *American journal of epidemiology* **165**, 1328-35 (2007).

11. Cesari, M. et al. Inflammatory markers and cardiovascular disease (The Health, Aging and Body Composition [Health ABC] Study). *Am J Cardiol* **92**, 522-8 (2003).

12. Ferrucci, L. et al. Subsystems contributing to the decline in ability to walk: bridging the gap between epidemiology and geriatric practice in the InCHIANTI study. *Journal of the American Geriatrics Society* **48**, 1618-25 (2000).

13. Deary, I.J. et al. The Lothian Birth Cohort 1936: a study to examine influences on cognitive ageing from age 11 to age 70 and beyond. *BMC geriatrics* **7**, 28 (2007).

14. Houlihan, L.M. et al. Common variants of large effect in F12, KNG1, and HRG are associated with activated partial thromboplastin time. *American journal of human genetics* **86**, 626-31 (2010).

15. Yuan, X. et al. Population-based genome-wide association studies reveal six loci influencing plasma levels of liver enzymes. *American journal of human genetics* **83**, 520-8 (2008).

16. Kooner, J.S. et al. Genome-wide scan identifies variation in MLXIPL associated with plasma triglycerides. *Nature genetics* **40**, 149-51 (2008).

17. Wain, L.V. et al. Genome-wide association study identifies six new loci influencing pulse pressure and mean arterial pressure. *Nature genetics* **43**, 1005-11 (2011).

18. Ehret, G.B. et al. Genetic variants in novel pathways influence blood pressure and cardiovascular disease risk. *Nature* **478**, 103-9 (2011).

19. Portas, L. et al. History, geography and population structure influence the distribution and heritability of blood and anthropometric quantitative traits in nine Sardinian genetic isolates. *Genetics research* **92**, 199-208 (2010).

20. Biino, G. et al. Genetic architecture of hand quantitative ultrasound measures: a population-based study in a Sardinian genetic isolate. *Bone* **46**, 1197-203 (2010).

21. Pistis, G. et al. High differentiation among eight villages in a secluded area of Sardinia revealed by genome-wide high density SNPs analysis. *PloS one* **4**, e4654 (2009).

22. McQuillan, R. et al. Runs of homozygosity in European populations. *American journal of human genetics* **83**, 359-72 (2008).

23. Volzke, H. et al. Cohort profile: the study of health in Pomerania. *International journal of epidemiology* **40**, 294-307 (2011).

24. Hofman, A., Grobbee, D.E., de Jong, P.T. & van den Ouweland, F.A. Determinants of disease and disability in the elderly: the Rotterdam Elderly Study. *European journal of epidemiology* **7**, 403-22 (1991).

25. Ikram, M.A. et al. The Rotterdam Scan Study: design and update up to 2012. *European journal of epidemiology* **26**, 811-24 (2011).

26. Fried, L.P. et al. The Cardiovascular Health Study: design and rationale. *Ann Epidemiol* **1**, 263-76 (1991).

27. Morabia, A., Bernstein, M., Heritier, S. & Ylli, A. Community-based surveillance of cardiovascular risk factors in Geneva: methods, resulting distributions, and comparisons with other populations. *Preventive medicine* **26**, 311-9 (1997).

28. Beer-Borst, S., Costanza, M.C., Pechere-Bertschi, A. & Morabia, A. Twelve-year trends and correlates of dietary salt intakes for the general adult population of Geneva, Switzerland. *European journal of clinical nutrition* **63**, 155-64 (2009).

29. Morabia, A. & Costanza, M.C. The obesity epidemic as harbinger of a metabolic disorder epidemic: trends in overweight, hypercholesterolemia, and diabetes treatment in Geneva, Switzerland, 1993-2003. *American journal of public health* **95**, 632-5 (2005).

30. Ciullo, M. et al. New susceptibility locus for hypertension on chromosome 8q by efficient pedigree-breaking in an Italian isolate. *Human molecular genetics* **15**, 1735-43 (2006).

31. Colonna, V. et al. Campora: a young genetic isolate in South Italy. *Human heredity* **64**, 123-35 (2007).

32. Ciullo, M. et al. Identification and replication of a novel obesity locus on chromosome 1q24 in isolated populations of Cilento. *Diabetes* **57**, 783-90 (2008).

33. Heid, I.M. et al. Meta-analysis of the INSIG2 association with obesity including 74,345 individuals: does heterogeneity of estimates relate to study design? *PLoS genetics* **5**, e1000694 (2009).

34. Colonna, V. et al. Comparing population structure as inferred from genealogical versus genetic information. *European journal of human genetics : EJHG* **17**, 1635-41 (2009).

35. Siervo, M. et al. Angiogenesis and biomarkers of cardiovascular risk in adults with metabolic syndrome. *Journal of internal medicine* **268**, 338-47 (2010).

36. de Moor, M.H. et al. Meta-analysis of genome-wide association studies for personality. *Molecular psychiatry* **17**, 337-49 (2012).

37. Ruggiero, D. et al. Genetics of VEGF serum variation in human isolated populations of cilento: importance of VEGF polymorphisms. *PloS one* **6**, e16982 (2011).

38. Sorice, R. et al. Association of a variant in the CHRNA5-A3-B4 gene cluster region to heavy smoking in the Italian population. *European journal of human genetics : EJHG* **19**, 593-6 (2011).

39. Girotto, G. et al. Hearing function and thresholds: a genome-wide association study in European isolated populations identifies new loci and pathways. *Journal of medical genetics* **48**, 369-74 (2011).

40. Terracciano, A. et al. Meta-analysis of genome-wide association studies identifies common variants in CTNNA2 associated with excitement-seeking. *Translational psychiatry* **1**, e49 (2011).

41. Luciano, M. et al. Longevity candidate genes and their association with personality traits in the elderly. *American journal of medical genetics. Part B, Neuropsychiatric genetics : the official publication of the International Society of Psychiatric Genetics* **159B**, 192-200 (2012).

42. Holle, R., Happich, M., Lowel, H. & Wichmann, H.E. KORA--a research platform for population based health research. *Gesundheitswesen* **67 Suppl 1**, S19-25 (2005).

43. Wichmann, H.E., Gieger, C. & Illig, T. KORA-gen--resource for population genetics, controls and a broad spectrum of disease phenotypes. *Gesundheitswesen* **67 Suppl 1**, S26-30 (2005).

44. Winkelmann, B.R. et al. Rationale and design of the LURIC study--a resource for functional genomics, pharmacogenomics and long-term prognosis of cardiovascular disease. *Pharmacogenomics* **2**, S1-73 (2001).

45. Lind, L., Fors, N., Hall, J., Marttala, K. & Stenborg, A. A comparison of three different methods to evaluate endothelium-dependent vasodilation in the elderly: the Prospective Investigation of the Vasculature in Uppsala Seniors (PIVUS) study. *Arteriosclerosis, thrombosis, and vascular biology* **25**, 2368-75 (2005).

46. Richards, J.B. et al. Bone mineral density, osteoporosis, and osteoporotic fractures: a genome-wide association study. *Lancet* **371**, 1505-12 (2008).

47. Soranzo, N. et al. Meta-analysis of genome-wide scans for human adult stature identifies novel Loci and associations with measures of skeletal frame size. *PLoS genetics* **5**, e1000445 (2009).

48. Caulfield, M. et al. Genome-wide mapping of human loci for essential hypertension. *Lancet* **361**, 2118-23 (2003).

49. Urabe, Y. et al. A genome-wide association study of nephrolithiasis in the Japanese population identifies novel susceptible Loci at 5q35.3, 7p14.3, and 13q14.1. *PLoS genetics* **8**, e1002541 (2012).

50. Tanikawa, C. et al. A genome-wide association study identifies two susceptibility loci for duodenal ulcer in the Japanese population. *Nature genetics* **44**, 430-4, S1-2 (2012).

51. Kamatani, Y. et al. Genome-wide association study of hematological and biochemical traits in a Japanese population. *Nature genetics* **42**, 210-5 (2010).

52. Chambers, J.C. et al. Common genetic variation near MC4R is associated with waist circumference and insulin resistance. *Nature genetics* **40**, 716-8 (2008).

53. Chambers, J.C. et al. Genome-wide association study identifies variants in TMPRSS6 associated with hemoglobin levels. *Nature genetics* **41**, 1170-2 (2009).

54. Chambers, J.C. et al. Common genetic variation near melatonin receptor MTNR1B contributes to raised plasma glucose and increased risk of type 2 diabetes among Indian Asians and European Caucasians. *Diabetes* **58**, 2703-8 (2009).
