# Supplementary material for: Meta-Analysis of Genome-Wide Association Studies Identifies Six New Loci for Serum Calcium Concentrations
Source: PLoS Genet. 2013 Sep 19;9(9):e1003796. doi: 10.1371/journal.pgen.1003796 (PMC3778004; doi:10.1371/journal.pgen.1003796)
Supplement: Table S13 — Genotyping information for each cohort (discovery, replication and look-ups). (DOCX) [file pgen.1003796.s021.docx]

## Table S13: Genotyping information for each cohort (discovery, replication and look-ups)

|  | **Array type** | **Genotype calling** | **QC filters for genotyped SNPs used for imputation** | **No of SNPs used for imputation** | **Imputation** | **Imputation Backbone for phased CEU haplotypes (NCBI build)** | **Filtering of imputed genotypes** | **Data management and statistical analysis** | **Population stratification or Principal Components** |
| --- | --- | --- | --- | --- | --- | --- | --- | --- | --- |
| **Discovery cohorts** |  |  |  |  |  |  |  |  |  |
| **AGES** | Illumina 370CNV | BeadChip array | call rate < 97%, HWE deviation < 1 x 10-6, mishap (PLINK haplotype-based test for non-random missing genotype data[2]) p < 1 x 10-9, and mismatched positions between Illumina, dbSNP and/or HapMap | 325.094 | MACH version 1.0.16 | Hapmap CEU release 22 build 36 | None | ProABEL | No adjustment for population stratification, as no significant stratification exists within the cohort. |
| **ARIC** | Affy 6.0 | Birdseed | call rate <95%, MAF<1%, pHWE <10E-5 | 669'450 | MACH v1.0.16 | HapMap release 22 (build 36) | none | ProbABEL, PLINK, R | Significant association was obaserved between some of the top 10 principle components estimated using Eigenstrat. The appropriate PCs were therefore included in the respective stratum as covariates in the association analyses for eGFRcys, eGFRcrea, and CKD. |
| **BLSA** | Illumina 550K | Beadstudio | pHWE<10e-4, call rate < 99%, MAF < 1%, Mendellian error | 514'027 | MACH version 1.0.16 | HapMap release 22 (build 36) | none | MERLIN, R | Adjust for first 2 PCA generated from eigenstrat |
| **CoLaus Study** | Affymetrix 500K | BRLMM | pHWE<1e-7; individual call rate <90%; SNP call rate<70%; MAF<0.01 | 390'631 | IMPUTE v0.2 | HapMap release 21 (build 35) | none | QUICKTEST | We included the first 3 principal components as covariates |
| **CROATIA-Korcula** | Illumina HapMap370CNV | BeadStudio | pHWE<1e-6, call rate<98%, MAF<0.01, | 307'625 | MACH v 1.0.16 | HapMap release 22 (build 36) | none | R , GenABEL (mmscore to account for relationships), ProbABEL, | adjustment for first 3 PC |
| **CROATIA-Split** | Illumina HapMap370CNV | BeadStudio | pHWE<1e-6, call rate<98%, MAF<0.01, | 321'456 | MACH v 1.0.16 | HapMap release 22 (build 36) | none | R , GenABEL (mmscore to account for relationships), ProbABEL, | adjustment for first 3 PC |
| **CROATIA-Vis** | Illumina HapMap300v1 | BeadStudio | pHWE<1e-6, call rate<98%, MAF<0.01, | 289'827 | MACH v 1.0.16 | HapMap release 22 (build 36) | none | R , GenABEL (mmscore to account for relationships), ProbABEL, | adjustment for first 3 PC |
| **Framingham Heart Study** | Affymetrix 500K  Affymetrix 50K supplemental | Affymetrix | pHWE<1e-6, call rate<97%, mishap p<1e-9, MAF<0.01, Mendelian errors>100, SNPs not in Hapmap or strandedness issues merging with Hapmap | 378'163 | MACH version 1.0.15 | HapMap release 22 (build 36) | none | R, linear mixed effect models and GEE models, robust variance option to account for relatedness | We adjusted for the first PC estimated from Eigenstrat that was associated with calcium levels (P<0.05).[^1^](#_ENREF_1) |
| **HABC** | Illumina Human1M-Duo | Illumina BeadStudio | pHWE<1e-6, call rate<97%, MAF<0.01, | 914263 | MACH version 1.0.16 | Hapmap CEU release 22 build 36 | None | R | We adjusted for the first PC estimated from Eigenstrat that was associated with calcium levels (P<0.05). [^1^](#_ENREF_1) |
| **INCHIANTI** | Illumina 550K | Beadstudio | pHWE<10e-6, call rate < 99%, MAF < 1%, Mendellian error | 498'838 | MACH version 1.0.16 | HapMap release 22 (build 36) | none | MERLIN, R | Genomic control adjustment |
| **Lothian Birth Cohort 1936** | Illumina 610 quad v1 | Illumina | SNPs were included if : call rate ≥ 0.98, MAF ≥ 0.01, and HWE P ≥ 0.001. SNPs not in Hapmap and those with ambiguous for strand were excluded. | 535'709 | Mach v1.0.16 | HapMap release 22 (build 36) | none | Linear regression analysis for an additive genetic model was performed using MACH2QTL software, incorporating dosage information and including age and sex, and also albumin as covariates in the model. The files were prepared as requested using R and Perl. | None |
| **LOLIPOP EW610** | Illumina Human610 | BeadStudio | call rate <=90%, MAF<1%, pHWE <= 10E-6 | 544'620 | MACH | HapMap release 22 (build 36) | none | mach2qtl | Adjusted for the first 10 PCs |
| **LOLIPOP EW A** | Affymetrix 500K | BRLMN | call rate <= 90%, MAF < 1%, pHWE <= 10E-6 | 374'773 | MACH | HapMap release 21 (build 35) | none | mach2qtl | Adjusted for the first 10 PCs |
| **LOLIPOP EW P** | Perlegen custom | NA | call rate <= 90%, MAF < 1%, pHWE <= 10E-6 | 184'469 | MACH | HapMap release 21 (build 35) | none | mach2qtl | Adjusted for the first 10 PCs |
| **Ogliastra Genetic Park - Talana Study** | Affymetrix 500K | Affymetrix | pHWE<1e-6, call rate<95%, MAF<0.01 | 329'122 | MACH version 1.0.16 | HapMap release 22 (build 36) | Rsq < 0.3 | R, GenABEL,ProbABEL (mmscore function was used to account for relatedness) | none |
| **ORCADES** | Illumina HapMap300v2 | BeadStudio | pHWE<1e-6, call rate<98%, MAF<0.01, | 285'491 | MACH v 1.0.16 | HapMap release 22 (build 36) | none | R , GenABEL (mmscore to account for relationships), ProbABEL, | adjustment for first 3 PC |
| **SHIP** | Affymetrix SNP 6.0 | Birdseed2 | none | 869'224 | IMPUTE v0.5.0 | HapMap release 22 (build 36) | duplicate RSID but different positions | QUICKTEST version 0.95 (Params: --method-score), InforSense, InterSystems Caché | We observed no population stratification using principle components estimated using Eigenstrat.[^1^](#_ENREF_1) |
| **The Cardiovascular Health Study (CHS)** | Illumina 370CNV BeadChip system. | Illumina BeadStudio software | call rate < 97%, HWE P < 10-5, > 2 duplicate errors or Mendelian inconsistencies (for reference CEPH trios), heterozygote frequency = 0, SNP not found in HapMap. | 306'655 | BIMBAM v0.99 | HapMap release 22 (build 36) | SNPs were excluded for variance on the allele dosage ≤0.01 | R, robust SE estimates | No principal components were adjusted |
| **The Rotterdam Study** | Version 3 Illumina Infinium II HumanHap550 | BeadStudio | pHWE<1e-5, call rate<90%, MAF<0.01, Mendelian errors>100, SNPs not in Hapmap or strandedness issues merging with Hapmap | 530'683 | MACH | HapMap release 22 (build 36) | none | R, ProbABEL | NA |
| **Replication cohorts** |  |  |  |  |  |  |  |  |  |
| **BRIGHT** | Affymetrix 500K | CHIAMO | pHWE <1e-7, call rate <95%, MAF<0.01 | 387'666 | IMPUTE | HapMap release 22 (build 35) | none | PLINK | na |
| **Bus Santé study** | NA | De novo genotyping using KASPar v4.0 after whole genome amplification by primer extension pre-amplification (PEP) using thermostable DNA polymerases | NA | NA | NA | NA | NA | Stata 11.0 (Stata Corp, College Station, USA) , linear regression | NA |
| **INGI-Carlantino-Project** | Illumina 370K | Illumina | include SNPs with MAF >= 0.05, include only SNPs with a 90% genotyping rate, pHWE <= <1e-6 | 374'498 | MACH | HapMap release 22 (build 36) | Rsq=0.3 : Markers imputed with Rsq value < Rsq filtered; MAF=5: markers < than this value will be filtered. Accepted value are <=0.5 and >=1. if the number is >=1 then it indicates the number of chromosome which is calculated as MAF*2 chromosomes. | R, GenABEL, mmscore | Because of the presence of close relatives in our dataset, statistical analyses were performed by using kinship matrix, estimated through implemention in GenABEL |
| **INGI-FVG-Project** | Illumina 370K | Illumina | include SNPs with MAF >= 0.05, include only SNPs with a 90% genotyping rate, pHWE <= <1e-6 | 374'498 | MACH | HapMap release 22 (build 36) | Rsq=0.3 : Markers imputed with Rsq value < Rsq filtered; MAF=5: markers < than this value will be filtered. Accepted value are <=0.5 and >=1. if the number is >=1 then it indicates the number of chromosome which is calculated as MAF*2 chromosomes. | R, GenABEL, mmscore | Because of the presence of close relatives in our dataset, statistical analyses were performed by using kinship matrix, estimated through implemention in GenABEL |
| **INGI-CILENTO** | 370 K Illumina (859 individuals) 700 K Illumina (288 individuals) | Illumina | Imputation was performed in the two groups (859 and 288 individuals) separately, using the following filters: call rate<95%, MAF<1%, SNPs not in HapMap. For the directly typed SNPs, in common between the two groups, the real genotype was used in the association analysis, while the imputation dosage was considered for the other SNPs. | 306995 (for the 859 individuals genotyped with the 370K) 588083 (for the 288 individuals genotyped with the 700K) | MACH v1.0.16 | HapMap release 22 (build 36) | none | R, linear model, GenABEL and ProbABEL (mmscore function was used to account for relatedness) |  |
| **KORA F3** | Affymetrix 500K | BRLMM | pHWE<1e-6, individual call rate<93%, snp call rate<95%, MAF<0.01 | 379'392 | Impute v1.0.0 | HapMap release 22 (build 36) | none | R, linear models | none |
| **KORA F4** | Affymetrix 6.0 (1000K) | Birdseed2 | On chip level only subjects with overall genotyping efficiencies of at least 93% were included resulting in an average genotyping efficiency of 98% per chip. In addition the called sex had to agree with the sex in the KORA study database. | 909'622 | Impute v0.4.2 | HapMap release 22 (build 36) | none | R, linear models | none |
| **LURIC** | Affymetrix 6.0 | Birdseed | individual callrate <96% | 866'316 | MACH | HapMap release 22 (build 36) | none | SNPTEST v2.1.1 |  |
| **PIVUS** | Human Omni Express and Metabochip | Illumina | For SNPs with MAF >=0.05: pHWE<1e-6, call rate<95%; For SNPs with MAF <0.05: pHWE<1e-6, call rate<99%; MAF<0.01 | 738'879 | IMPUTE version 2.1.2 | HapMap release 22 (build 36) | none | SNPTEST | Adjusted for the first 2 PCs estimated from MDS using PLINK |
| **SHIP-Trend** | Illumina Human Omni 2.5 | GenomeStudio Genotyping Module v1.0 | excluded: pHWE <= 0.0001 or CallRate <= 0.9 or monomorphic SNPs | 1'782'967 | IMPUTE v2.1.2.3 | HapMap release 22 (build 36) | duplicate RSID but different positions | QUICKTEST version 0.95 (Params: --method-mean), InforSense, InterSystems Caché | We observed no population stratification using principle components estimated using Eigenstrat.[^1^](#_ENREF_1) |
| **TwinsUK** | HumanHap300  HumanHap610Q | Illluminus calling algorithm | pHWE<1e-6, MAF<0.01, SNP call rate <97% (SNPs with MAF>=5%) or < 99% (for 1% <= MAF < 5%) | up to 553,487 depending the dataset | IMPUTE V2.0 | HapMap release 22 (build 36) | none | MERLIN | we excluded all the samples with evidence of non-European ancestry as assessed by PCA comparison with HapMap3 populations |
| **Other ethnicities** |  |  |  |  |  |  |  |  |  |
| **Japan biobank** | Human610-Quad BeadChip | Illumina | pHWE<1e-6, call rate<97%, mishap p<1e-9, MAF<0.01, Mendelian errors>100, SNPs not in Hapmap or strandedness issues merging with Hapmap |  | MACH version 1.0.15 | HapMap release 22 (build 36) | none | P-link linear regression analysis adjusted with age and gender | no adjustment |
| **LOLIPOP IA P** | Perlegen custom | NA | call rate <= 90%, MAF < 1%, pHWE <= 10E-6 | 170'055 | MACH | HapMap release 21 (build 35) | none | mach2qtl | Adjusted for the first 10 PCs |
| **LOLIPOP IA317** | Illumna HumanHap300K | BeadStudio | call rate <= 90%, MAF < 1%, pHWE <= 10E-6 | 245'892 | MACH | HapMap release 21 (build 35) | none | mach2qtl | Adjusted for the first 10 PCs, CHD, and a recruitment factor |
| **LOLIPOP IA610** | Illumina Human610 | BeadStudio | call rate <= 90%, MAF < 1%, pHWE <= 10E-6 | 544'390 | MACH | HapMap release 21 (build 35) | none | mach2qtl | Adjusted for the first 10 PCs |

References

1. Price, A.L. et al. Principal components analysis corrects for stratification in genome-wide association studies. *Nature genetics* **38**, 904-9 (2006).
