# Supplementary material for: Meta-Analysis of Genome-Wide Association Studies Identifies Six New Loci for Serum Calcium Concentrations
Source: PLoS Genet. 2013 Sep 19;9(9):e1003796. doi: 10.1371/journal.pgen.1003796 (PMC3778004; doi:10.1371/journal.pgen.1003796)
Supplement: Text S1 — Study specific acknowledgements. (DOCX) [file pgen.1003796.s022.docx]

# Acknowledgments

For the CNV analysis, we thank Ben Voight for kindly providing the CtS list used by the GIANT consortium.

**Discovery cohorts**

**AGES:** This study has been funded by NIH contract N01-AG-1-2100, the NIA Intramural Research Program, Hjartavernd (the Icelandic Heart Association), and the Althingi (the Icelandic Parliament). The study is approved by the Icelandic National Bioethics Committee, VSN: 00-063. The researchers are indebted to the participants for their willingness to participate in the study.

**ARIC :** The Atherosclerosis Risk in Communities Study is carried out as a collaborative study supported by National Heart, Lung, and Blood Institute contracts (HHSN268201100005C, HHSN268201100006C, HHSN268201100007C, HHSN268201100008C, HHSN268201100009C, HHSN268201100010C, HHSN268201100011C, and HSN268201100012C), R01HL087641, R01HL59367 and R01HL086694; National Human Genome Research Institute contract U01HG004402; and National Institutes of Health contract HHSN268200625226C. AK was supported by the Emmy Noether Programme of the German Research Foundation (KO3598/2-1).The authors thank the staff and participants of the ARIC study for their important contributions. Infrastructure was partly supported by Grant Number UL1RR025005, a component of the National Institutes of Health and NIH Roadmap for Medical Research.

**BLSA :** The BLSA was supported in part by the Intramural Research Program of the NIH, National Institute on Aging. A portion of that support was through a R&D contract with MedStar Research Institute.

**Cardiovascular Health Study (CHS) :** This CHS research was supported by NHLBI contracts N01-HC-85239, N01-HC-85079 through N01-HC-85086; N01-HC-35129, N01 HC-15103, N01 HC-55222, N01-HC-75150, N01-HC-45133, HHSN268201200036C and NHLBI grants HL080295, HL087652, HL105756 with additional contribution from NINDS. Additional support was provided through AG-023629, AG-15928, AG-20098, and AG-027058 from the NIA. See also <http://www.chs-nhlbi.org/pi.htm>. DNA handling and genotyping at Cedars-Sinai Medical Center was supported in part by the National Center for Research Resources, grant UL1RR033176, and is now at the National Center for Advancing Translational Sciences, CTSI grant UL1TR000124; in addition to the National Institute of Diabetes and Digestive and Kidney Disease grant DK063491 to the Southern California Diabetes Endocrinology Research Center.

**CoLaus :** The CoLaus authors thank Yolande Barreau, Mathieu Firmann, Vladimir Mayor, Anne-Lise Bastian, Binasa Ramic, Martine Moranville, Martine Baumer, Marcy Sagette, Jeanne Ecoffey and Sylvie Mermoud for data collection. The CoLaus study received financial contributions from GlaxoSmithKline, the Faculty of Biology and Medicine of Lausanne, the Swiss National Science Foundation (33CSCO-122661, 3200BO-111361/2, 3100AO-116323/1,310000-112552). The computations for CoLaus imputation were performed in part at the Vital-IT center for high performance computing of the Swiss Institute of Bioinformatics. MB is supported by the Swiss School of Public Health Plus (SSPH+). We thank Vincent Mooser for his contribution to the CoLaus study.

**CROATIA-Korcula :** The CROATIA-Korcula study was funded by grants from the Medical Research Council (UK), European Commission Framework 6 project EUROSPAN (Contract No. LSHG-CT-2006-018947) and Republic of Croatia Ministry of Science, Education and Sports research grants to I.R. (108-1080315-0302). We would like to acknowledge the invaluable contributions of the recruitment team in Korcula, the administrative teams in Croatia and Edinburgh and the people of Korcula. The SNP genotyping for the CROATIA-Korcula cohort was performed in Helmholtz Zentrum München, Neuherberg, Germany.

**CROATIA-Split :** The CROATIA-Split study is funded by grants from the Medical Research Council (UK), European Commission Framework 6 project EUROSPAN (Contract No. LSHG-CT-2006-018947) and Republic of Croatia Ministry of Science, Education and Sports research grants to I.R. (108-1080315-0302). We would like to acknowledge the staff of several institutions in Croatia that supported the field work, including but not limited to The University of Split and Zagreb Medical Schools and the Croatian Institute for Public Health. The SNP genotyping for the CROATIA-Split cohort was performed by AROS Applied Biotechnology, Aarhus, Denmark.

**CROATIA-Vis :** The CROATIA-Vis study was funded by grants from the Medical Research Council (UK) and Republic of Croatia Ministry of Science, Education and Sports research grants to I.R. (108-1080315-0302). We would like to acknowledge the staff of several institutions in Croatia that supported the field work, including but not limited to The University of Split and Zagreb Medical Schools, the Institute for Anthropological Research in Zagreb and Croatian Institute for Public Health. The SNP genotyping for the CROATIA-Vis cohort was performed in the core genotyping laboratory of the Wellcome Trust Clinical Research Facility at the Western General Hospital, Edinburgh, Scotland.

**Framingham Heart Study :** This research was conducted in part using data and resources from the Framingham Heart Study of the National Heart Lung and Blood Institute of the National Institutes of Health and Boston University School of Medicine.   The analyses reflect intellectual input and resource development from the Framingham Heart Study investigators participating in the SNP Health Association Resource (SHARe) project. This work was partially supported by the National Heart, Lung and Blood Institute's Framingham Heart Study (Contract No. N01-HC-25195) and its contract with Affymetrix, Inc for genotyping services (Contract No. N02-HL-6-4278). A portion of this research utilized the Linux Cluster for Genetic Analysis (LinGA-II) funded by the Robert Dawson Evans Endowment of the Department of Medicine at Boston University School of Medicine and Boston Medical Center.

**HABC:** This research was supported by NIA contracts N01AG62101, N01AG62103, and N01AG62106. The genome-wide association study was funded by NIA grant 1R01AG032098-01A1 to Wake Forest University Health Sciences and genotyping services were provided by the Center for Inherited Disease Research (CIDR). CIDR is fully funded through a federal contract from the National Institutes of Health to The Johns Hopkins University, contract number HHSN268200782096C. This research was supported in part by the Intramural Research Program of the NIH, National Institute on Aging.

**InCHIANTI :** The InCHIANTI study baseline (1998-2000) was supported as a "targeted project" (ICS110.1/RF97.71) by the Italian Ministry of Health and in part by the U.S. National Institute on Aging (Contracts: 263 MD 9164 and 263 MD 821336).

**London Life Sciences Population (LOLIPOP) study:** LOLIPOP EW A: We thank GSK for supporting and genotyping of the data. LOLIPOP EW P : The LOLIPOP study was supported by the British Heart Foundation Grant SP/04/002. LOLIPOP EW610 : The LOLIPOP study was supported by the Wellcome Trust. We thank the participants and research teams involved in LOLIPOP.

**Lothian Birth Cohort 1936** : The LBC1936 research was supported by a programme grant from Research Into Ageing and continues with programme grants from Help the Aged/Research Into Ageing (Disconnected Mind). The work was undertaken by The University of Edinburgh Centre for Cognitive Ageing and Cognitive Epidemiology, part of the cross council Lifelong Health and Wellbeing Initiative (G0700704/84698). Funding from the Biotechnology and Biological Sciences Research Council (BBSRC), Engineering and Physical Sciences Research Council (EPSRC), Economic and Social Research Council (ESRC) and Medical Research Council (MRC) is gratefully acknowledged. We thank the LBC1936 participants. We thank Alan Gow, Janie Corley, Caroline Brett, Caroline Cameron, Michelle Taylor, and Alison Pattie for data collection and data entry. We thank the study secretary Paula Davies. We thank the nurses and staff at the Wellcome Trust Clinical Research Facility, where subjects were tested and the genotyping was performed. We thank the staff at the Lothian Health Board. This whole genome association study was funded by the Biotechnology and Biological Sciences Research Council (BBSRC).

**Ogliastra Genetic Park - Talana Study:** We thank the Ogliastra population and all the individuals who participated in this study. We are very grateful to the municipal administrators for their collaboration to the project and for economic and logistic support. This work was supported by grants from the Italian Ministry of Education, University and Research (MIUR) no.5571/DSPAR/2002 and (FIRB) D. M. no. 718/Ric/2005.

**ORCADES :** ORCADES was supported by the Chief Scientist Office of the Scottish Government, the Royal Society, the MRC Human Genetics Unit, Arthritis Research UK and the European Union framework program 6 EUROSPAN project (contract no. LSHG-CT-2006-018947). DNA extractions were performed at the Wellcome Trust Clinical Research Facility in Edinburgh. We would like to acknowledge the invaluable contributions of Lorraine Anderson and the research nurses in Orkney, the administrative team in Edinburgh and the people of Orkney.

**SHIP:** SHIP is part of the Community Medicine Research net of the University of Greifswald, Germany, which is funded by the Federal Ministry of Education and Research (grants no. 01ZZ9603, 01ZZ0103, and 01ZZ0403), the Ministry of Cultural Affairs as well as the Social Ministry of the Federal State of Mecklenburg-West Pomerania, and the network ‘Greifswald Approach to Individualized Medicine (GANI_MED)’ funded by the Federal Ministry of Education and Research (grant 03IS2061A). Genome-wide data have been supported by the Federal Ministry of Education and Research (grant no. 03ZIK012) and a joint grant from Siemens Healthcare, Erlangen, Germany and the Federal State of Mecklenburg- West Pomerania. The University of Greifswald is a member of the ‘Center of Knowledge Interchange’ program of the Siemens AG. The SHIP authors are grateful to Mario Stanke for the opportunity to use his Server Cluster for SNP Imputation.

**The Rotterdam Study :** The Rotterdam Study is supported by the Erasmus Medical Center and Erasmus University Rotterdam; the Netherlands Organization for Scientific Research (NWO); the Netherlands Organization for Health Research and Development (ZonMw); the Research Institute for Diseases in the Elderly (RIDE); the Netherlands Heart Foundation; the Ministry of Education, Culture and Science; the Ministry of Health Welfare and Sports; the European Commission; and the Municipality of Rotterdam.
Support for genotyping was provided by the Netherlands Organisation of Scientific Research NWO Investments (nr. 175.010.2005.011, 911-03-012), the Research Institute for Diseases in the Elderly (014-93-015; RIDE2), the Netherlands Genomics Initiative (NGI)/Netherlands Consortium for Healthy Aging (NCHA) project nr. 050-060-810. Jacqueline Witteman is supported by NWO grant (vici, 918-76-619). Abbas Dehghan is supported by NOW grant (veni, ) and the EUR Fellowship.

**Replication cohorts**

**Bus Santé study :** This research was conducted in part using data and resources from the Bus Santé study of the Geneva University Hospitals.   This work was partially supported by the Geneva University Hospitals, the Canton of Geneva (Switzerland), the General Directorate of Health (Canton of Geneva), the Swiss School of Public Health Plus (SSPH+) and the Swiss Foundation for Science (Contract No. 33CM30-124087). The investigators of the Bus Santé study thank all the collaborators of the Unit of Population Epidemiology, UNILABS SA (Geneva), and Abbott Diagnostics (Baar, Switzerland). We thank. Dr Olivier GOLAZ, Central laboratory of clinical chemistry, Geneva University Hospitals.

**INGI-Carlantino-Project :** We thank Laura Esposito and Angela D’Eustacchio for technical support. We are very grateful to the municipal administrators for their collaboration on the project and for logistic support. We would like to thank all participants to this study.

**INGI-CILENTO :** We thank the populations of Cilento for their participation in the study. This work was supported by grants from the EU (Vasoplus-037254), the Italian Ministry of Universities (FIRB -RBIN064YAT), the Assessorato Ricerca Regione Campania, the Ente Parco Nazionale del Cilento e Vallo di Diano and the Fondazione Banco di Napoli to MC.

**INGI-FVG-Project :** We thank Laura Esposito and Angela D’Eustacchio for technical support. We are very grateful to the municipal administrators for their collaboration on the project and for logistic support. We would like to thank all participants to this study.

**KORA F3/F4 Studies (Cooperative Health Research in the Region of Augsburg):** The KORA Augsburg studies were financed by the Helmholtz Zentrum München, German Research Center for Environmental Health, Neuherberg, Germany and supported by grants from the German Federal Ministry of Education and Research (BMBF). Part of this work was financed by the German National Genome Research Network (NGFN). Our research was supported within the Munich Center of Health Sciences (MC Health) as part of LMUinnovativ.

**Ludwigshafen Risk and Cardiovascular Health (LURIC) Study:** LURIC thanks their participants and researchers and acknowledges that it has received funding through the 6th Framework Program (integrated project Bloodomics, grant LSHM-CT-2004-503485) and 7th of Framework Program (integrated project Atheroremo, Grant Agreement number 201668) of the European Union.

**PIVUS :** LL acknowledge funding from the Swedish Research council. AM and APM acknowledge funding from the Wellcome Trust (WT064890, WT081682)

**SHIP-Trend :** SHIP is part of the Community Medicine Research net of the University of Greifswald, Germany, which is funded by the Federal Ministry of Education and Research (grants no. 01ZZ9603, 01ZZ0103, and 01ZZ0403), the Ministry of Cultural Affairs as well as the Social Ministry of the Federal State of Mecklenburg-West Pomerania, and the network ‘Greifswald Approach to Individualized Medicine (GANI_MED)’ funded by the Federal Ministry of Education and Research (grant 03IS2061A). Genome-wide data have been supported by the Federal Ministry of Education and Research (grant no. 03ZIK012) and a joint grant from Siemens Healthcare, Erlangen, Germany and the Federal State of Mecklenburg- West Pomerania. The University of Greifswald is a member of the ‘Center of Knowledge Interchange’ program of the Siemens AG. The SHIP authors are grateful to Mario Stanke for the opportunity to use his Server Cluster for SNP Imputation.

**TwinsUK :** Twins UK (TUK): The study was funded by the Wellcome Trust; European Community’s Seventh Framework Programme (FP7/2007-2013)/grant agreement HEALTH-F2-2008-201865-GEFOS and (FP7/2007-2013), ENGAGE project grant agreement HEALTH-F4-2007-201413 and the FP-5 GenomEUtwin Project (QLG2-CT-2002-01254). The study also receives support from the Dept of Health via the National Institute for Health Research (NIHR) comprehensive Biomedical Research Centre award to Guy's & St Thomas' NHS Foundation Trust in partnership with King's College London. TDS is an NIHR senior Investigator. The project also received support from a Biotechnology and Biological Sciences Research Council (BBSRC) project grant (G20234). The authors acknowledge the funding and support of the National Eye Institute via an NIH/CIDR genotyping project (PI: Terri Young). NS is supported by the Wellcome Trust (Core Grant Number 091746/Z/10/Z). Genotyping of TwinsUK samples: We thank the staff from the Genotyping Facilities and Variation Informatics at the Wellcome Trust Sanger Institute for sample preparation, Quality Control and Genotyping led by Leena Peltonen and Panos Deloukas; Le Centre National de Génotypage, France, led by Mark Lathrop, for genotyping; Duke University, North Carolina, USA, led by David Goldstein, for genotyping; and the Finnish Institute of Molecular Medicine, Finnish Genome Center, University of Helsinki, led by Aarno Palotie. Genotyping was also performed by CIDR as part of an NEI/NIH project grant.

**The BRItish Genetics of HyperTension (BRIGHT) study:** The BRIGHT study was supported by the Medical Research Council of Great Britain (G9521010D), by the British Heart Foundation (grant number PG/02/128) and The Wellcome Trust as part of the Wellcome Trust Case Control Consortium. The BRIGHT study is extremely grateful to all the patients who participated in the study and the BRIGHT nursing team. We would also like to thank the Barts Genome Centre staff for their assistance with this project. This work forms part of the research themes contributing to the translational research portfolio for Barts and the London Cardiovascular Biomedical Research Unit, which is supported and funded by the National Institute for Health Research.

**Other studies**

**Biobank Japan :** This work was conducted as a part of the BioBank Japan Project that was supported by the Ministry of Education, Culture, Sports, Science and Technology of the Japanese government.

**GEFOS Consortium:** The Genetic Factors for Osteoporosis (GEFOS) consortium was funded by the European Commission (HEALTH-F2-2008-201865-GEFOS).

**London Life Sciences Population (LOLIPOP) study:** We thank the participants and research teams involved in LOLIPOP. LOLIPOP IA P : The LOLIPOP study was supported by the British Heart Foundation Grant SP/04/002. LOLIPOP IA317 : The LOLIPOP study was supported by the British Heart Foundation Grant SP/04/002. LOLIPOP IA610 : The LOLIPOP study was supported by the Wellcome Trust.

**Animal studies**

OB is supported by a Swiss National Foundation grant #PP00P3-133648 and by a bridge grant from the Faculté de Biologie et Médecine de l'Université de Lausanne.

**GEFOS COLLABORATORS**

Karol Estrada^1,2,3,139^, Unnur Styrkarsdottir^4,139^, Evangelos Evangelou^5,139^, Yi-Hsiang Hsu^6,7,139^, Emma L Duncan^8,9,139^, Evangelia E Ntzani^5,139^, Ling Oei^1,2,3,139^, Omar M E Albagha^10^, Najaf Amin^2^, John P Kemp^11^, Daniel L Koller^12^, Guo Li^13^, Ching-Ti Liu^14^, Ryan L Minster^15^, Alireza Moayyeri^16,17^, Liesbeth Vandenput^18^, Dana Willner^8,19^, Su-Mei Xiao^20,21^, Laura M Yerges-Armstrong^22^, Hou-Feng Zheng^23^, Nerea Alonso^10^, Joel Eriksson^18^, Candace M Kammerer^15^, Stephen K Kaptoge^16^, Paul J Leo^8^, Gudmar Thorleifsson^4^, Scott G Wilson^17,24,25^, James F Wilson^26,27^, Ville Aalto^28,29^, Markku Alen^30^, Aaron K Aragaki^31^, Thor Aspelund^32,33^, Jacqueline R Center^34,35,36^, Zoe Dailiana^37^, David J Duggan^38^, Melissa Garcia^39^, Natàlia Garcia-Giralt^40^, Sylvie Giroux^41^, Göran Hallmans^42^, Lynne J Hocking^43^, Lise Bjerre Husted^44^, Karen A Jameson^45^, Rita Khusainova^46,47^, Ghi Su Kim^48^, Charles Kooperberg^31^, Theodora Koromila^49^, Marcin Kruk^50^, Marika Laaksonen^51^, Andrea Z Lacroix^31^, Seung Hun Lee^48^, Ping C Leung^52^, Joshua R Lewis^24,25^, Laura Masi^53^, Simona Mencej-Bedrac^54^, Tuan V Nguyen^34,35^, Xavier Nogues^40^, Millan S Patel^55^, Janez Prezelj^56^, Lynda M Rose^57^, Serena Scollen^58^, Kristin Siggeirsdottir^32^, Albert V Smith^32,33^, Olle Svensson^59^, Stella Trompet^60,61^, Olivia Trummer^62^, Natasja M van Schoor^63^, Jean Woo^64^, Kun Zhu^24,25^, Susana Balcells^65^, Maria Luisa Brandi^53^, Brendan M Buckley^66^, Sulin Cheng^67,68^, Claus Christiansen^69^, Cyrus Cooper^45^, George Dedoussis^70^, Ian Ford^71^, Morten Frost^72,73^, David Goltzman^74^, Jesús González-Macías^75,76^, Mika Kähönen^77,78^, Magnus Karlsson^79^, Elza Khusnutdinova^46,47^, Jung-Min Koh^48^, Panagoula Kollia^49^, Bente Lomholt Langdahl^44^, William D Leslie^80^, Paul Lips^81,82^, Östen Ljunggren^83^, Roman S Lorenc^50^, Janja Marc^54^, Dan Mellström^18^, Barbara Obermayer-Pietsch^62^, José M Olmos^75,76^, Ulrika Pettersson-Kymmer^84^, David M Reid^43^, José A Riancho^75,76^, Paul M Ridker^57,85^, François Rousseau^41,86,87^, P Eline Slagboom^88,3^, Nelson LS Tang^89,90^, Roser Urreizti^65^, Wim Van Hul^91^, Jorma Viikari^92,93^, María T Zarrabeitia^94^, Yurii S Aulchenko^2^, Martha Castano-Betancourt^1,2,3^, Elin Grundberg^95,96,97^, Lizbeth Herrera^1^, Thorvaldur Ingvarsson^98,99,33^, Hrefna Johannsdottir^4^, Tony Kwan^95,96^, Rui Li^100^, Robert Luben^16^, Carolina Medina-Gómez^1,2^, Stefan Th Palsson^4^, Sjur Reppe^101^, Jerome I Rotter^102^, Gunnar Sigurdsson^103,33^, Joyce B J van Meurs^1,2,3^, Dominique Verlaan^95,96^, Frances MK Williams^17^, Andrew R Wood^104^, Yanhua Zhou^14^, Kaare M Gautvik^101,105,106^, Tomi Pastinen^95,96,107^, Soumya Raychaudhuri^108,109^, Jane A Cauley^110^, Daniel I Chasman^57,85^, Graeme R Clark^8^, Steven R Cummings^111^, Patrick Danoy^8^, Elaine M Dennison^45^, Richard Eastell^112^, John A Eisman^34,35,36^, Vilmundur Gudnason^32,33^, Albert Hofman^2,3^, Rebecca D Jackson^113,114^, Graeme Jones^115^, J Wouter Jukema^60,116,117^, Kay-Tee Khaw^16^, Terho Lehtimäki^118,119^, Yongmei Liu^120^, Mattias Lorentzon^18^, Eugene McCloskey^112,121^, Braxton D Mitchell^22^, Kannabiran Nandakumar^6,7^, Geoffrey C Nicholson^122^, Ben A Oostra^123^, Munro Peacock^124^, Huibert A P Pols^1,2^, Richard L Prince^24,25^, Olli Raitakari^28,29^, Ian R Reid^125^, John Robbins^126^, Philip N Sambrook^127^, Pak Chung Sham^128,129^, Alan R Shuldiner ^22,130^, Frances A Tylavsky^131^, Cornelia M van Duijn^2^, Nick J Wareham^132^, L Adrienne Cupples^14,133^, Michael J Econs^124,12^, David M Evans^11^, Tamara B Harris^39^, Annie Wai Chee Kung^20,21^, Bruce M Psaty^134,135^, Jonathan Reeve^136^, Timothy D Spector^17^, Elizabeth A Streeten^22,130^, M Carola Zillikens^1^, Unnur Thorsteinsdottir^4,33,140^, Claes Ohlsson^18,140^, David Karasik^6,7,140^, J Brent Richards^137,17,140^, Matthew A Brown^8,140^, Kari Stefansson^4,33,140^, André G Uitterlinden^1,2,3,140^, Stuart H Ralston^10,140^, John P A Ioannidis^138,5,140^, Douglas P Kiel^6,7,140^, Fernando Rivadeneira^1,2,3,140^

Affiliations:

^1^Department of Internal Medicine, Erasmus Medical Center, Rotterdam, The Netherlands.  ^2^Department of Epidemiology, Erasmus Medical Center, Rotterdam, The Netherlands.  ^3^Netherlands Genomics Initiative (NGI)-sponsored Netherlands Consortium for Healthy Aging (NCHA), Leiden, The Netherlands.  ^4^deCODE Genetics, Reykjavik, Iceland.  ^5^Department of Hygiene and Epidemiology, University of Ioannina, Ioannina, Greece.  ^6^Institute for Aging Research, Hebrew SeniorLife, Boston, USA.  ^7^Department of Medicine, Harvard Medical School, Boston, USA.  ^8^Human Genetics Group, University of Queensland Diamantina Institute, Brisbane, Australia.  ^9^Department of Endocrinology, Royal Brisbane and Women's Hospital, Brisbane, Australia.  ^10^Rheumatic Diseases Unit, Institute of Genetics and Molecular Medicine, University of Edinburgh, Edinburgh, UK.  ^11^Medical Research Council (MRC) Centre for Causal Analyses in Translational Epidemiology, University of Bristol, Bristol, UK.  ^12^Department of Medical and Molecular Genetics, Indiana University School of Medicine, Indianapolis, USA.  ^13^Cardiovascular Health Research Unit, University of Washington, Seattle, USA.  ^14^Department of Biostatistics, Boston University School of Public Health, Boston, USA.  ^15^Department of Human Genetics, University of Pittsburgh, Pittsburgh, PA, USA.  ^16^Department of Public Health and Primary Care, University of Cambridge, Cambridge, UK.  ^17^Department of Twin Research and Genetic Epidemiology, King's College London, London, UK.  ^18^Centre for Bone and Arthritis Research, Institute of Medicine, Sahlgrenska Academy, University of Gothenburg, Gothenburg, Sweden.  ^19^Australian Centre for Ecogenomics, University of Queensland, Brisbane, Australia.  ^20^Department of Medicine, The University of Hong Kong, Hong Kong, China.  ^21^Research Centre of Heart, Brain, Hormone and Healthy Aging, The University of Hong Kong, Hong Kong, China.  ^22^Department of Medicine, Division of Endocrinology, Diabetes and Nutrition, University of Maryland School of Medicine, Baltimore, MD, USA.  ^23^Department of Human Genetics, Lady Davis Institute, McGill University, Montreal, Canada.  ^24^School of Medicine and Pharmacology, University of Western Australia, Perth, Australia.  ^25^Department of Endocrinology and Diabetes, Sir Charles Gairdner Hospital, Perth, Australia.  ^26^Centre for Population Health Sciences, University of Edinburgh, Edinburgh, UK.  ^27^MRC Human Genetics Unit, MRC Institute of Genetics and Molecular Medicine at the University of Edinburgh, Edinburgh, UK.  ^28^Department of Clinical Physiology, Turku University Hospital, Turku, Finland.  ^29^Research Centre of Applied and Preventive Cardiovascular Medicine, University of Turku, Turku, Finland.  ^30^Department of Medical Rehabilitation, Oulu University Hospital and Institute of Health Sciences, Oulu, Finland.  ^31^Division of Public Health Sciences, Fred Hutchinson Cancer Research Center, Seattle, USA.  ^32^Icelandic Heart Association, Kopavogur, Iceland.  ^33^Faculty of Medicine, University of Iceland, Reykjavik, Iceland.  ^34^Osteoporosis and Bone Biology Program, Garvan Institute of Medical Research, Sydney, Australia.  ^35^Department of Medicine, University of New South Wales, Sydney, Australia.  ^36^Department of Endocrinology, St Vincents Hospital, Sydney, Australia.  ^37^Department of Orthopaedic Surgery, Medical School University of Thessalia, Larissa, Greece.  ^38^Translational Genomics Research Institute, Phoenix, USA.  ^39^Laboratory of Epidemiology, Demography, and Biometry, National Institute on Aging, Bethesda, MD, USA.  ^40^Department of Internal Medicine, Hospital del Mar, Instituto Municipal de Investigación Médica (IMIM), Red Temática de Investigación Cooperativa en Envejecimiento y Fragilidad (RETICEF), Universitat Autònoma de Barcelona (UAB), Barcelone, Spain. ^41^Unité de recherche en génétique humaine et moléculaire, Centre de recherche du Centre hospitalier universitaire de Québec - Hôpital St-François-d'Assise (CHUQ/HSFA), Québec City, Canada.  ^42^Department of Public Health and Clinical Medicine, Umeå Unviersity, Umeå, Sweden.  ^43^Musculoskeletal Research Programme, Division of Applied Medicine, University of Aberdeen, Aberdeen, UK.  ^44^Department of Endocrinology and Internal Medicine, Aarhus University Hospital, Aarhus C, Denmark.  ^45^MRC Lifecourse Epidemiology Unit, University of Southampton, Southampton, UK.  ^46^Ufa Scientific Centre of Russian Academy of Sciences, Institute of Biochemistry and Genetics, Ufa, Russia.  ^47^Biological Department, Bashkir State University, Ufa, Russia.  ^48^Division of Endocrinology and Metabolism, Asan Medical Center, University of Ulsan College of Medicine, Seoul, South Korea.  ^49^Department of Genetics and Biotechnology, Faculty of Biology, University of Athens, Athens, Greece.  ^50^Department of Biochemistry and Experimental Medicine, The Children's Memorial Health Institute, Warsaw, Poland.  ^51^Department of Food and Environmental Sciences, University of Helsinki, Helsinki, Finland.  ^52^Jockey Club Centre for Osteoporosis Care and Control, The Chinese University of Hong Kong, Hong Kong SAR, China.  ^53^Department of Internal Medicine, University of Florence, Florence, Italy.  ^54^Department of Clinical Biochemistry, University of Ljubljana, Ljubljana, Slovenia.  ^55^Department of Medical Genetics, University of British Columbia, Vancouver, Canada.  ^56^Department of Endocrinology, University Medical Center, Ljubljana, Slovenia.  ^57^Division of Preventive Medicine, Brigham and Women's Hospital, Boston, USA.  ^58^Department of Medicine, University of Cambridge, Cambridge, UK.  ^59^Department of Surgical and Perioperative Sciences, Umeå Unviersity, Umeå, Sweden.  ^60^Department of Cardiology, Leiden University Medical Center, Leiden, The Netherlands.  ^61^Department of Gerontology and Geriatrics, Leiden University Medical Center, Leiden, The Netherlands.  ^62^Department of Internal Medicine, Division of Endocrinology and Metabolism, Medical University Graz, Graz, Austria.  ^63^Department of Epidemiology and Biostatistics, Extramuraal Geneeskundig Onderzoek (EMGO) Institute for Health and Care Research, Vrije Universiteit (VU) University Medical Center, Amsterdam, The Netherlands.  ^64^Department of Medicine and Therapeutics, The Chinese University of Hong Kong, Hong Kong SAR, China.  ^65^Department of Genetics, University of Barcelona, Centro de Investigación Biomédica en Red de Enfermedades Raras (CIBERER), Institut de Biomedicina de la Universitat de Barcelona (IBUB), Barcelone, Spain.  ^66^Department of Pharmacology and Therapeutics, University College Cork, Cork, Ireland.  ^67^Department of Health Sciences, University of Jyväskylä, Jyväskylä, Finland.  ^68^Department of Orthopaedics and Traumatology, Kuopio University Hospital, Kuopio, Finland.  ^69^Center for Clinical and Basic Research (CCBR)-Synarc, Ballerup, Denmark.  ^70^Department of Nutrition and Dietetics, Harokopio University, Athens, Greece.  ^71^Robertson Center for Biostatistics, University of Glasgow, Glasgow, United Kingdom.  ^72^Department of Endocrinology, Odense University Hospital, Odense, Denmark.  ^73^Clinical Institute, University of Southern Denmark, Odense, Denmark.  ^74^Department of Medicine, McGill University, Montreal, Canada.  ^75^Department of Medicine, University of Cantabria, Santander, Spain.  ^76^Department of Internal Medicine, Hospital Universitario Marqués de Valdecilla and Instituto de Formación e Investigación Marqués de Valdecilla (IFIMAV), Santander, Spain.  ^77^Department of Clinical Physiology, Tampere University Hospital, Tampere, Finland.  ^78^Department of Clinical Physiology, University of Tampere School of Medicine, Tampere, Finland.  ^79^Clinical and Molecular Osteoporosis Research Unit, Department of Clinical Sciences and Department of Orthopaedics, Lund University, Malmö, Sweden.  ^80^Department of Internal Medicine, University of Manitoba, Winnipeg, Canada.  ^81^Department of Endocrinology, Vrije Universiteit (VU) University Medical Center, Amsterdam, The Netherlands.  ^82^Extramuraal Geneeskundig Onderzoek (EMGO) Institute for Health and Care Research, Vrije Universiteit (VU) University Medical Center, Amsterdam, The Netherlands.  ^83^Department of Medical Sciences, University of Uppsala, Uppsala, Sweden.  ^84^Department of Pharmacology and Neuroscience, Umeå University, Umeå, Sweden.  ^85^Harvard Medical School, Boston, USA.  ^86^Department of Molecular Biology, Medical Biochemistry and Pathology, Université Laval, Québec City, Canada.  ^87^The APOGEE-Net/CanGèneTest Network on Genetic Health Services and Policy, Université Laval, Québec City, Canada.  ^88^Department of Molecular Epidemiology, Leiden University Medical Center, Leiden, The Netherlands.  ^89^Department of Chemical Pathology, The Chinese University of Hong Kong, Hong Kong SAR, China.  ^90^Li Ka Shing Institute of Health Sciences, The Chinese University of Hong Kong, Hong Kong SAR, China.  ^91^Department of Medical Genetics, University of Antwerp, Antwerp, Belgium.  ^92^Department of Medicine, Turku University Hospital, Turku, Finland.  ^93^Department of Medicine, University of Turku, Turku, Finland.  ^94^Department of Legal Medicine, University of Cantabria, Santander, Spain.  ^95^Department of Human Genetics, McGill University, Montreal, Canada.  ^96^McGill University and Genome Québec Innovation Centre, Montreal, Canada.  ^97^Wellcome Trust Sanger Institute, Hinxton, UK.  ^98^Department of Orthopedic Surgery, Akureyri Hospital, Akureyri, Iceland.  ^99^Institution of Health Science, University Of Akureyri, Akureyri, Iceland.  ^100^Department of Epidemiology and Biostatistics, Lady Davis Institute, McGill University, Montreal, Canada.  ^101^Department of Medical Biochemistry, Oslo University Hospital, Oslo, Norway.  ^102^Medical Genetics Institute, Cedars-Sinai Medical Center, Los Angeles, USA.  ^103^Department of Endocrinology and Metabolism, University Hospital, Reykjavik, Iceland.  ^104^Genetics of Complex Traits, Peninsula College of Medicine and Dentistry, University of Exeter, Exeter, England.  ^105^Department of Clinical Biochemistry, Lovisenberg Deacon Hospital, Oslo, Norway.  ^106^Institute of Basic Medical Sciences, University of Oslo, Oslo, Norway.  ^107^Department of Medical Genetics, McGill University Health Centre, Montreal, Canada.  ^108^Division of Genetics and Rheumatology, Brigham and Women's Hospital, Harvard Medical School, Boston, United States.  ^109^Program in Medical and Population Genetics, Broad Institute, Cambridge, United States.  ^110^Department of Epidemiology, University of Pittsburgh, Pittsburgh, USA.  ^111^California Pacific Medical Center, San Francisco, CA, USA.  ^112^National Institute for Health and Research (NIHR) Musculoskeletal Biomedical Research Unit, University of Sheffield, Sheffield, UK.  ^113^Department of Internal Medicine, The Ohio State University, Columbus, USA.  ^114^Center for Clinical and Translational Science, The Ohio State University, Columbus, USA.  ^115^Menzies Research Institute, University of Tasmania, Hobart, Australia.  ^116^Durrer Center for Cardiogenetic Research, Amsterdam, The Netherlands.  ^117^Interuniversity Cardiology Institute of the Netherlands, Utrecht, The Netherlands.  ^118^Department of Clinical Chemistry, Tampere University Hospital, Tampere, Finland.  ^119^Department of Clinical Chemistry, University of Tampere School of Medicine, Tampere, Finland.  ^120^Center for Human Genomics, Wake Forest University School of Medicine, Winston-Salem, NC, USA.  ^121^Academic Unit of Bone Metabolism, Metabolic Bone Centre, University of Sheffield, Sheffield, UK.  ^122^Rural Clinical School, The University of Queensland, Toowoomba, Australia.  ^123^Department of Clinical Genetics, Erasmus Medical Center, Rotterdam, The Netherlands.  ^124^Department of Medicine, Indiana University School of Medicine, Indianapolis, USA.  ^125^Department of Medicine, University of Auckland, Auckland, New Zealand.  ^126^Department of Medicine, University of Davis, Sacramento, CA, USA.  ^127^Kolling Institute, Royal North Shore Hospital, University of Sydney, Sydney, Australia.  ^128^Department of Psychiatry, The University of Hong Kong, Hong Kong, China.  ^129^Centre for Reproduction, Development and Growth, The University of Hong Kong, Hong Kong, China.  ^130^Geriatric Research and Education Clinical Center (GRECC), Veterans Administration Medical Center, Baltimore, MD, USA.  ^131^Department of Preventive Medicine, University of Tennessee College of Medicine, Memphis, TN, USA.  ^132^MRC Epidemiology Unit Box 285, Medical Research Council, Cambridge, UK.  ^133^Framingham Heart Study, Framingham, USA.  ^134^Departments of Medicine, Epidemiology and Health Services, University of Washington, Seattle, USA.  ^135^Group Health Research Institute, Group Health Cooperative, Seattle, USA.  ^136^Medicine box 157, University of Cambridge, Cambridge, UK.  ^137^Departments of Medicine, Human Genetics, Epidemiology and Biostatistics, Lady Davis Institute, McGill University, Montreal, Canada.  ^138^Stanford Prevention Research Center, Stanford University, Stanford, USA.

^139^These authors contributed equally to this work.

^140^These authors jointly directed this work.
